# Adherence thresholds for emtricitabine-tenofovir disoproxil fumarate preexposure prophylaxis against HIV acquisition in cisgender women: A randomized directly observed dosing study

**DOI:** 10.1371/journal.pmed.1004732

**Published:** 2025-09-09

**Authors:** Kenneth K. Mugwanya, Matilda Saina, Nelly R. Mugo, Samantha MaWhinney, Mary Morrow, Torin T. Schaafsma, Deborah Donnell, David V. Glidden, Kenneth Ngure, Clare E. Brown, Elena A. Rechkina, Bhavna H. Chohan, Linxuan Wu, Elex Hill, Eric Koome, Nina Akelo, Sarah Mbaire, Susan A. Morrison, Mary Kibatha, Irene Njeru, Marianne Muriithi, Corwin Coppinger, Lane Bushman, Jared M. Baeten, Peter L. Anderson, for the Women Benchmark Study Team

**Affiliations:** 1 Department of Global Health, University of Washington, Seattle, Washington, United States of America; 2 Department of Epidemiology, University of Washington, Seattle, Washington, United States of America; 3 Kenya Medical Research Institute, Nairobi, Kenya; 4 University of Colorado Anschutz Medical Campus, Aurora, Colorado, United States of America; 5 Fred Hutchinson Center, Seattle, Washington, United States of America; 6 University of California San Francisco, San Francisco, California, United States of America; 7 Jomo Kenyatta University of Agriculture and Technology, Nairobi, Kenya; 8 Department of Medicine, University of Washington, Seattle, Washington, United States of America; University of Southampton, UNITED KINGDOM OF GREAT BRITAIN AND NORTHERN IRELAND

## Abstract

**Background:**

Oral emtricitabine/tenofovir disoproxil fumarate (F/TDF) preexposure prophylaxis (PrEP) effectiveness against HIV acquisition highly depends on adherence. For men who have sex with men, a dosing study in the United States (US) population defined clinically meaningful tenofovir diphosphate (TFV-DP) thresholds in dried blood spots (DBS) based on the rounded 25th percentile for 2, 4, and 7 doses/week as 350, 700, and 1,250 fmol/punch. However, divergent efficacy results in the first generation randomized clinical trials of F/TDF PrEP among African women led to several hypotheses to question whether the pharmacology and adherence requirement for oral F/TDF PrEP may be different in cisgender women compared to what is already established for men.

**Methods and findings:**

We conducted an open-label, parallel, randomized directly observed dosing (DOD) study of oral F/TDF PrEP among women without HIV who were not pregnant or breastfeeding in Kenya. Participants were randomly assigned to 2, 4, or 7 DOD F/TDF doses/week for 8 weeks. Blood was collected weekly, and TFV-DP and emtricitabine triphosphate (FTC-TP) concentrations in DBS and peripheral blood mononuclear cells (PBMCs) were quantified using validated liquid chromatography-tandem mass spectrometry. For DBS, concentrations were quantified from a 3-mm punch using the 70% methanol/30% water (70:30) extraction method as the primary process—the same method used for the original TFV-DP benchmarks derived in US adults, and additionally with 50% methanol/50% water (50:50) extraction using punches from the same DBS spot to compare the extraction performance of 70:30 versus 50:50 methods. The primary outcome was the steady-state fitted concentrations of TFV-DP and dose proportionality in DBS and the observed PBMC TFV-DP levels by study dosing groups. Secondary outcomes included the quantitative concentrations of FTC-TP in DBS, TFV-DP half-life in DBS, and the relative TFV-DP recovery from DBS using the 70:30 versus 50:50 extraction method. One-compartment population pharmacokinetic models were fit to estimate steady-state DBS concentrations. Descriptive statistics were summarized as range, means, and medians with interquartile range (IQR) for continuous outcomes and proportions for categorical variables. Fifty-four women were enrolled and randomized. Median age was 22 (IQR, 20–25) years. The observed median (IQR) week 8 TFV-DP concentrations in DBS were 359 (266–464), 749 (596–923), and 1,389 (1,151–1,551) fmol/punch after 2, 4, and 7 doses/week, respectively. At week 8, FTC-TP was quantifiable in 71%, 19%, and 0% DBS samples for 7, 4, and 2 doses/week groups, respectively. Fitted median (IQR) steady-state DBS TFV-DP concentrations were 416 (316, 516), 832 (631, 1,033), and 1,457 (1,106, 1,808) fmol/punch for 2, 4, and 7 doses/week, respectively, similar to previous estimates in US adults. TFV-DP exhibited a mean half-life of 17.5 days (95%CI: 16.7, 18.4) in DBS and steady-state TFV-DP concentrations varied in direct proportion to the dosing frequency [slope: 1.02 90% confidence interval 0.84, 1.20]. The 50:50 DBS extraction method yielded 1.27 (95% CI 1.25, 1.28) higher TFV-DP concentrations compared to the 70:30 method. When the 1.27 conversion factor was applied to the original 70:30 method-derived TFV-DP thresholds, the updated TFV-DP adherence interpretation benchmarks based on the 50:50 extraction were: <450 for <2 dose/week, 450–899 for 2–3 doses/week, 900–1,599 for 4–6 doses/week, and ≥1,600 fmol/punch for 7 doses/week. The observed mean (standard deviation) steady-state PBMC TFV-DP concentrations was 11.99 ± 8.47, 31.81 ± 15.66, and 63.1 ± 28.97 fmol/10^6^ cells after 2, 4, and 7 doses/week, respectively. Overall, oral F/TDF PrEP was well tolerated. No grade 3 or higher adverse events were observed during the dosing phase. The primary study limitation was dosing for 8 weeks, but population pharmacokinetic modeling enabled steady-state estimates.

**Conclusions:**

Steady-state DBS TFV-DP concentrations from directly observed F/TDF PrEP dosing in African cisgender women participants are similar to previous estimates defined from US-based participants. These data demonstrate that cisgender women achieve similar DBS and PBMC TFV-DP concentrations as men for the same adherence level and validate the original TFV-DP benchmarks to interpret F/TDF adherence in HIV prevention studies and PrEP programs among cisgender women.

**Trial registration:**

Clinicaltrials.gov: NCT05057858

## Author summary

**Why was this study done?:**

- Adherence is key for the effectiveness of oral preexposure prophylaxis (PrEP) against HIV acquisition. Pharmacological measures of cumulative adherence, such as tenofovir diphosphate (TFV-DP) in dried blood spots (DBS), have become key to accurate interpretation of HIV prevention trials and programs globally.
- For men who have sex with men, directly observed studies of PrEP with oral emtricitabine/tenofovir disoproxil fumarate (F/TDF) conducted among the United States (US) adults helped to establish the adherence benchmarks for this population.
- However, very low TFV-DP in DBS was observed in the first-generation F/TDF PrEP studies among African women. This led to scientific and public health uncertainty about whether the pharmacology of TFV-DP in DBS was different in African women, resulting in a need for different adherence thresholds compared to those derived in the US-based participants.

**What did the researchers do and find?:**

- We conducted a directly observed dosing study of oral F/TDF PrEP among cisgender women without HIV in Kenya to define the relationship between F/TDF PrEP dosing and TFV-DP concentrations in DBS and peripheral blood mononuclear cells (PBMCs) in women.
- We demonstrate that consistent with previous studies, TFV-DP concentrations varied in direct proportion to adherence, and the resulting thresholds are similar to the original adherence estimates defined for men from studies conducted in the US.
- Contrary to our expectation, the steady-state TFV-DP estimates from Kenyan women more closely overlapped and were nearly identical to estimates reported for the subgroup of US men than they were to concentrations from the US females in the previous DOTDBS study among US-based participants.
- Extracting DBS with 50% methanol:50% water process resulted in 1.27 (95% CI 1.25, 1.28) higher TFV-DP recovery compared to the original 70% methanol:30% water extraction process.
- Similarly, TFV-DP concentrations in PBMCs were also within range of levels observed from other studies among men and women.

**What do these findings mean?:**

- These data validate the original adherence benchmarks initially defined for men to interpret adherence to F/TDF PrEP in cisgender women.
- These findings also mean that for more than 15 years, cisgender women have been disenfranchised with the wrong hypothesis and counseling messages requiring them to be super adherers to oral F/TDF PrEP compared to men.
- Extraction method-dependent differences in TFV-DP yields underline that adherence interpretations should be based on TFV-DP benchmark thresholds that are consistent with the appropriate DBS extraction method reported by the testing laboratory.
- The primary limitation of this study was dosing for 8 weeks, which is about 90% of TFV-DP steady-state in DBS. However, population pharmacokinetic modeling enabled steady-state estimates.

## Abstract 3

Using a directly observed dosing study of oral emtricitabine/tenofovir disoproxil fumarate (F/TDF) pre-exposure prophylaxis (PrEP) for the prevention of HIV, Kenneth Mugwanya and colleagues show that cisgender women achieve PrEP drug concentrations in blood similar to those reported in men, dispelling any perceived need for different adherence thresholds in African women.

## Background

Preexposure prophylaxis (PrEP) with oral emtricitabine/tenofovir disoproxil fumarate (F/TDF) is an effective and recommended HIV prevention strategy in diverse populations, but its effectiveness is highly dependent on adherence [1–3]. Long half-life pharmacologic measures of adherence, such as intracellular tenofovir diphosphate (TFV-DP) in dried blood spots (DBS) [4], have become key to accurate interpretation of HIV prevention trials and programs. These measures, however, require a clear understanding of the relationship between consistency of dosing and the resulting drug concentrations in diverse populations. The DOT-DBS study defined the expected concentration of TFV-DP in DBS after directly observed F/TDF in the United States (US) men and women [4]. From this study, the median (interquartile range, [IQR]) TFV-DP values were 416 (347, 498), 856 (714, 1,026), and 1,534 (1,280, 1,837) fmol/punch for 2, 4, and 7 doses per week, respectively. The rounded 25th percentiles of TFV-DP were then used to define adherence benchmarks representing 2, 4, and 7 TDF/FTC doses/week which were 350, 700, and 1,250 fmol/punch [4]. These benchmarks have been used globally to assess PrEP adherence and HIV prevention outcomes across multiple clinical trials and programs of F/TDF PrEP [5–9]. When these thresholds were applied to the clinical cohort from the iPrEx study, they helped to define drug concentrations associated with HIV protection for men [10]. Specifically, a value of ≥700 fmol/punch (i.e., 4 doses/week on average) has been associated with high PrEP efficacy across multiple studies among men who have sex with men [10].

The relationship between drug dosing and the resultant TFV-DP in DBS can differ according to biological differences among diverse populations. For instance, the DOT-DBS study observed 17.6% lower TFV-DP in men compared with women and 14% lower in African–Americans than Whites [4]. Nevertheless, subgroups by site, race, and sex were all within <15% of adherence benchmarks noted above, supporting these interpretations across populations. However, for African women, large PrEP studies found that only 62% and 44% of individuals taking ≥4 doses/week and ≥6 oral PrEP doses/week were correctly identified based on daily medication electronic adherence monitoring using US-derived TFV-DP thresholds [11]. This raised the question as to whether these US-derived TFV-DP benchmarks can be applied to African women. Correct interpretation of adherence is critical in women because tissue pharmacology studies show lower active drug concentrations in vaginal tissue than in rectal tissue, which has led to some suggestions that women may require a high level of adherence to achieve the same protection benefits as men [12]. Whether the thresholds derived from US-based participants accurately apply to cisgender women, particularly those in Africa, is unclear. The overall goal of this work was to conduct a comprehensive dosing study to define TFV-DP adherence thresholds for oral F/TDF PrEP in a population of African women.

## Methods

### Ethical considerations

The study protocol is registered at Clinicaltrials.gov: NCT05057858. The study was reviewed and approved by NIH DAIDS Prevention Science Review Committee (DAIDS-ES ID 38807), the University of Washington Human Subjects Ethical Committee (STUDY00011136), and the Scientific and Ethics Review Committee of the Kenya Medical Research Institute (protocol number 4137). All participants provided written informed consent prior to participation and received IRB-approved compensation equivalent to $5 per visit for their time. The study was monitored by a protocol safety team comprised of the Protocol Co-Chairs, NIH DAIDS Medical Officer and Program Officer, site clinician, and the study safety monitor, that reviewed study progress every 6 months.

### Study design and population

We conducted an open-label, 8-week parallel, randomized, pharmacokinetic study of directly observed dosing (DOD) of oral F/TDF at frequencies of 2, 4, and 7 doses per week to define the expected concentration thresholds of TFV-DP in DBS for African women at the Kenya Medical Research Institute Thika site in Kenya. The study product was brand Truvada (Gilead Sciences) tablet, formulated as emtricitabine 200 mg-tenofovir disoproxil fumarate 300 mg, purchased from an accredited wholesale pharmacy in Kenya. This trial was registered with ClinicalTrials.gov (https://clinicaltrials.gov/study/NCT05057858) as NCT05057858 on September 16, 2021. The study is reported as per the Consolidated Standards of Reporting Trials (S1 File).

Study participants were healthy volunteer women without HIV. Eligible participants were 18–30 years, with estimated creatinine clearance ≥60 ml/min, no current and past use of HIV PrEP within the previous 12 months, not pregnant or breastfeeding, and at low risk for acquiring HIV given the possibly being assigned to non-daily F/TDF dosing schedules. HIV risk was assessed based on self-reported behavior and epidemiological factors defined by the Kenya national PrEP guidelines to indicate a substantial ongoing risk of acquiring HIV [13]. These factors include self-report of any of a) inconsistent condom use; b) having a sex partner(s) who is high risk and whose HIV status is unknown; c) engaging in transactional sex; d) ongoing intimate partner violence and gender-based violence; e) recent bacterial sexually transmitted infection; f) recurrent use of post-exposure prophylaxis; g) recurrent sex under the influence of alcohol/recreational drugs; and h) injection drug use with shared needles and/or syringes. Major exclusion criteria included, Hepatitis B virus surface antigen positivity screening, hemoglobin <10 mg/dL, or taking any investigational agents, aminoglycosides, or products with the same or similar active ingredients or close analogs of FTC and tenofovir, and no significant medical conditions that would interfere with study participation in the opinion of the investigators.

### Randomization and masking

Participants were randomly assigned 1:1:1 to a single tablet of oral F/TDF for 2 days, 4 days, or 7 (daily) days per week. Randomization was implemented via the REDCap randomization module. A pre-created randomization schedule was generated with blocked randomization stratified by age group (<25 versus ≥25) and uploaded to the REDCap randomization module by the study statistician. For each eligible and consenting participant, the site study coordinator accessed the password-protected randomization page to execute the randomization, and randomization occurred by pressing the “Randomize” button. Participants and study staff were not masked to the participants’ assigned study arm, but the random allocation lists used randomly varying block sizes to ensure allocations were unpredictable.

### Procedures

Participant demographic, sexual behavior, and medical history, including weight, height, and body mass index, were obtained at screening, which occurred within 2 weeks of randomization. Testing for complete blood count, hepatitis B virus surface antigen, and creatinine clearance estimated by the Cockcroft–Gault equation was conducted at baseline and week 8 visit. HIV rapid testing and urine testing for beta human chorionic gonadotropin for pregnancy were performed at baseline, week 4, and week 8. All women received a full package of HIV prevention services, including risk reduction counseling, condoms, and, as needed, etiological STI testing and treatment at each visit. Per study protocol, participants were deemed not fully evaluable for purpose of subsequent follow-up if they missed any two consecutive directly observed doses or if they failed to provide any expected pharmacologic sample. After participants were determined to be unevaluable, they were not expected to have subsequent follow-up, but they contributed data up to the visit they were censored in the intent-to-treat analysis.

Dosing started on the day of randomization, and doses could be taken anytime in the 24-h period of a dosing day without regard to mealtimes. Participants assigned to the 2 doses/week arm took doses on Monday and Tuesday, and the 4 doses/week arm took doses on Monday, Tuesday, Thursday, and Friday (S1 Fig). All doses were directly observed in person or by live video streaming on a smart cell phone via WhatsApp. Participants were provided internet data bundles to support video streaming. For doses taken via video streaming, participants were asked to open their mouth to confirm the dose was swallowed, and study personnel recorded the time of dosing. After 8 weeks of DOD, clients had a post-DOD phase, with two post-DOD visits staggered any time between 1 and 8 weeks after the last DOD dose.

All samples were collected, processed, and stored according to standard operating procedures developed by the University of Colorado Antiviral Pharmacology laboratory [4], the same processes and laboratory used for the DOT-DBS study that generated the US population-derived TFV-DP benchmarks [4]. The laboratory participates in the NIH-supported Clinical Pharmacology Quality Assurance program of assay method external review and approval and periodic proficiency testing [14]. Blood was collected in an EDTA tube at baseline, day 4, and then weekly throughout the 8-week DOD PrEP period for whole blood, DBS, and peripheral blood mononuclear cells (PBMCs) (S1 Fig). For the post-DOD phase, each participant had at least two post-dosing sampling visits, with the first visit occurring within 48 hours of last dose, and the second visit was staggered any time between 2 and 8 weeks after the last DOD. For DBS, 50 µl of whole blood from EDTA tube was spotted 5 times onto a Whatman 903 card using a pipette. Cards were air-dried for at least 3 hours or overnight at room temperature and then stored in plastic bags at −80 °C until analysis. During processing we identified seven defective DBS cards (two in the two doses study arm, three in the four doses study arm and one in daily dosing arm). Visual inspection of the cards showed the color looked different from the rest of the study DBS cards. For these seven DBS cards, the analytic lab used paired whole blood collected from the respective participant at the same visit to spot replacement DBS cards that were used for analysis. For PBMCs, blood from the same EDTA tube was centrifuged with lymphocyte separation medium. The PBMC buffy layer was removed into a separate tube, followed by washes, red cell lysis, and PBMC cells were manually counted, and stored at −80 °C until analysis. All samples were shipped on dried ice for centralized testing at the University of Colorado.

TFV-DP and emtricitabine triphosphate (FTC-TP) concentrations were quantified in DBS and PBMCs using validated liquid chromatography tandem mass spectrometry methods at the Colorado Antiviral Pharmacology Laboratory (CLIA 06D1094710) [15,16]. Briefly, TFV-DP and FTC-TP concentrations from DBS were quantified from a 3-mm punch using a previously validated methodology [15,16]. The 3-mm punch was extracted with 500 µl of 70% methanol/30% water (70:30) solution, creating a lysed cellular matrix that was subjected to sample clean-up and application to the liquid chromatography tandem mass spectrometry system. The assay was linear from 25 to 6,000 fmol/sample for TFV-DP and 0.1 to 200 pmol/sample or (100–200,000 fmol) for FTC-TP.

The original TFV-DP adherence benchmarks [17] were generated using the same 70:30 extraction method, but this extraction was revalidated using a 50% methanol and 50% water extraction method (50:50) for DBS, which yields higher drug and more reproducible drug recoveries. We performed paired extractions of DBS from the same spot to compare TFV-DP recoveries from DBS between the 70:30 versus 50:50 extraction processes to generate updated TFV-DP interpretations based on the 50:50 extraction procedure.

For PBMCs, cells were lysed and suspended in 500 μl cold 70% methanol/30% water (70:30) solution and stored at ‒80 °C until analysis. The PBMC assay quantifiable linear range was 5–6,000 fmol/sample for TFV-DP and 0.1–200 pmol/ sample or (100–200,000 fmol) for FTC-TP. Two million cells were typically assayed. Typically, 2 million cells were assayed (i.e., the sample) and PBMC TFV-DP and FTC-TP concentrations were reported as fmol (TFV-DP) or pmol (FTC-TP) per 10^6^cells.

### Outcomes

The primary outcome was the steady-state fitted concentrations of TFV-DP and dose proportionality in DBS and steady-state observed PBMC TFV-DP by study dosing groups. Secondary outcomes included the quantitative concentrations of FTC-TP in DBS and PBMCs, TFV-DP half-life in DBS, and the relative proportions of extracting TFV-DP and FTC-TP in DBS using the 70:30 compared to 50:50 extraction process. Drug levels in vaginal tissue and during pregnancy are not presented in the current report. Detailed definitions of outcomes are provided in the Protocol (S2 File).

### Statistical analysis

The sample size for drug concentration parameters was based on ensuring precision in the estimates to accurately describe the concentration kinetics of TFV-DP metabolite in African women, informed by previous DOD PrEP studies [4,18]. The precision desired for the mean TFV-DP in DBS at steady-state was within ±15% of the true population mean. The mean (± standard deviation [SD]) TFV-DP at steady-state in US adults without HIV following directly observed doses of 300 mg TDF/200 mg FTC once daily for 12 weeks was 1,605 ± 405 fmol/punch (coefficient of variation of 25.2%) [4]. Based on this variability, to be 90% confident that the DBS TFV-DP sample mean was within 15% of the true mean, a target fully evaluable sample size (i.e., participants with no more than two expected DOD missed and all pharmacokinetic samples collected) of 15 per group was required. We enrolled an additional 3 per group for a total of 18 participants per group to account for attrition. The study was not designed or powered to assess safety or efficacy outcomes.

Descriptive statistics were summarized as range, means, and medians with interquartile range (IQR) for continuous outcomes and proportions for categorical variables. Outcomes are presented as overall and by F/TDF dosing regimens. For quantitative summary estimates, TFV-DP and FTC-TP concentrations below the limit of quantification were included in analyses as half of the assay’s lower limit of quantification. Graphical plots were used to depict drug concentration-time profile by study arm. Observed drug concentrations were summarized as medians with IQR. To characterize the observed steady-state concentrations in PBMCs, we used mixed-effect models accounting for within-person correlation using all concentrations at weeks 2, 3, 4, 5, 6, 7, and 8 of dosing.

Mixed-effect models were used to assess the influence of key biologic variables, including age, BMI, estimated creatinine clearance, and hematocrit, on TFV-DP concentrations. Dose proportionality was determined based on the power model [ln(TFV-DP) = *µ* + beta*ln(dosing study arm) + error] using observed week 8 TFV-DP concentrations. Dose proportionality was defined as achieving a 90% confidence interval for the log(dose) coefficient within the limits (0.8, 1.25) [19]. Steady-state TFV-DP concentrations for varying dosing frequency and half-lives for TFV-DP in DBS were estimated by a one-compartment nonlinear mixed effects population pharmacokinetic model using all available concentrations across all time points. TFV-DP concentrations at steady-state were summarized as median, IQR, mean, SD, and 95% CI by study arms. The TFV-DP half-life in DBS was estimated based on the first-order kinetics via the exponential decay fit to the post-DOD washout period.

To compare the performance of 70:30 versus 50:50 DBS extraction process, a linear regression on the logarithmic scale was used to compare the quantitative concentration yield from the two extraction methods from paired 3-mm punch samples from the same DBS spot. The model fold difference in concentration between the extraction methods was then applied to the original 70:30 TFV-DP adherence interpretations from the DOT-DBS study, which were <350 (<2 dose/week), 350–699 (2–3 doses/week), 700–1,249 (4–6 doses/week), and ≥1,250 fmol/punch (7 doses/week) to generate updated adherence thresholds for the 50:50 extraction process. Safety was assessed as the frequency of any grade 3 or higher adverse events using the Division of AIDS toxicity tables [20], overall and by study arm. Analyses were done using R software 4.4.2. The study protocol (S2 File) and statistical analysis plan (S3 File) are provided as supporting files.

## Results

**Fig 1:**
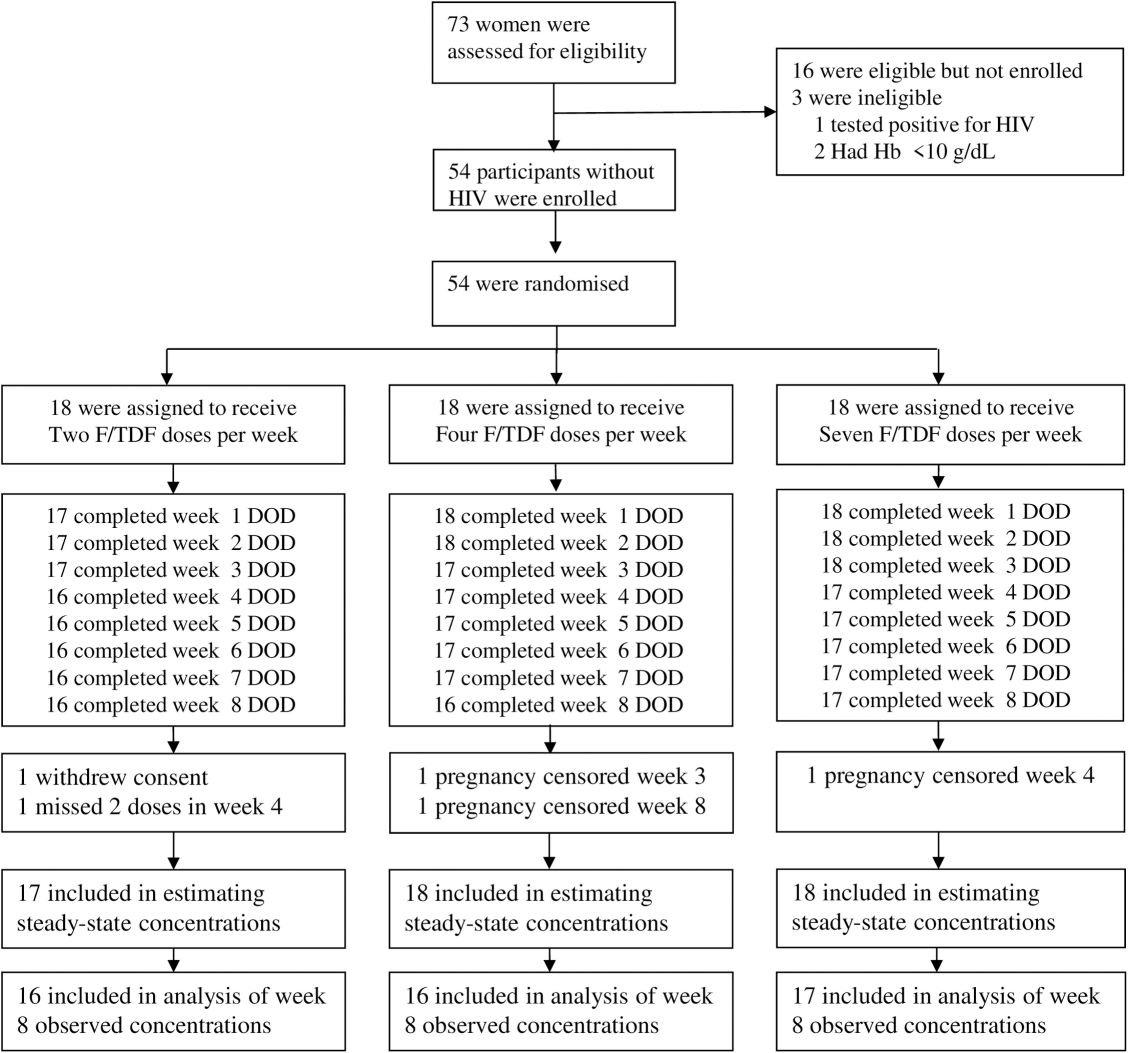
Trial profile. Consort diagram for the trial profile for enrollment and follow-up by the directly observed dosing (DOD) study arms with emtricitabine/tenofovir disoproxil fumarate (F/TDF) preexposure prophylaxis. Per study protocol, participants were deemed not fully evaluable for purpose of subsequent follow-up if they missed any two consecutive DOD or if they failed to provide any expected pharmacologic sample. When participants were determined to be unevaluable, they were not expected to have subsequent follow-up, but they contributed data up to visit they were censored in the intent-to-treat analysis. Urine βHCG for pregnancy was done at baseline and every 4 weeks (i.e., week 4 and 8).

From May 09, 2022, to January 20, 2023, 73 women were screened, of whom 54 were enrolled and randomized (Fig 1), and followed up until May 12, 2023. Participant baseline characteristics by study group are summarized in Table 1. The median (IQR) age was 22 (20–25) years, and creatinine clearance estimated by the Cockcroft–Gault equation was 132 (119–150) mL/min. Of 54 randomized, 49 were fully evaluable (i.e., provided the full set of all expected pharmacologic samples and did not miss more than one DOD). Five participants were not fully evaluable per protocol: two participants in the 2 doses per week arm (one withdrew consent in week 1 and the second missed two consecutive doses during week 4 dosing), two participants in the 4 doses per week arm (pregnancy-censored, one during week 3 and the other during week 8 dosing), and one participant in the daily dosing arm (pregnancy-censored during week 4 dosing). All five participants who were not fully evaluable per protocol contributed data to the visit when they were censored.

**Table 1:** Participant characteristics by study arm.

	Overall (*N* = 54)	2 doses/week (*N* = 18)	4 doses/week (*N* = 18)	7 doses/week (*N* = 18)
	*n*/*N* (%) or median (IQR)	*n*/*N* (%) or Median (IQR)	*n*/*N* (%) or Median (IQR)	*n*/*N* (%) or Median (IQR)
Age (years)	22 (20–25)	24 (19–25)	22 (19–24)	23 (20–25)
Weight (kg)	57 (53.0–65.5)	57.2 (53.9–63.6)	54.4 (52.4–67.2)	57.7 (53.4–63.4)
Body Mass Index	21.9 (20.5–24.5)	221.9 (20.9–25.9)	20.9 (19.8–23.0)	22.6 (20.5–26.1)
Hemoglobin (g/dL)	14 (13.4–14.6)	14.1 (13.6–15.0)	14 (13.5–14.5)	14.1 (13.3–14.6)
Hematocrit (%)	41 (39.0–43.8)	41.6 (40.1–44.8)	40.7 (39.9–43.5)	41 (38.9–43.5)
Creatinine clearance (mL/min)	132 (119–150)	137 (124–160)	130 (120–148)	131 (113–146)
Contraceptive method				
None	22/54 (41%)	7/18 (39%)	8/18 (44%)	7/18 (39%)
Implant	16/54 (30%)	4/18 (22%)	5/18 (28%)	7/18 (39%)
Condoms	7/54 (13%)	3/18 (17%)	2/18 (11%)	2/18 (11%)
OCP	4/54 (7%)	1/18 (6%)	2/18 (11%)	1/18 (6%)
Injectable	2/54 (4%)	1/18 (6%)	1/18 (6%)	0/18 (0%)
Other	3/54 (6%)	2/18 (11%)	0/18 (0%)	1/18 (6%)
Marital status				
Never married	44/54 (83%)	17/18 (94%)	15/18 (83%)	13/18 (72%)
Cohabiting	1/54 (2%)	0/18 (0%)	0/18 (0%)	1/18 (6%)
Married, monogamous	8/54 (15%)	1/16 (6%)	3/18 (17%)	4/18 (22%)
Education level				
Primary level, not complete	0/54 (0%)	0/18 (0%)	0/18 (0%)	0/18 (0%)
Primary level, complete	8/54 (15%)	1/18 (6%)	3/18 (17%)	4/18 (22%)
Secondary level, not complete	4/54 (7%)	0/18 (0%)	2/18 (11%)	2/18 (11%)
Secondary level, complete	24/54 (44%)	7/18 (39%)	8/18 (44%)	9/18 (50%)
Attended post-secondary	18/54 (33%)	10/18 (56%)	5/18 (28%)	3/18 (17%)
% doses observed in person	1 (0.94–1.00)	1 (1.00–1.00)	1 (0.94–1.00)	0.99 (0.93–1.00)

Continuous variables summarized as median with interquartile range (IQR), and categorical variables as frequencies and proportions. Oral contraception pill (OCP), Other—contraception methods include intrauterine contraception device, and emergency pill. Creatinine clearance was estimated using the Cockcroft–Gault equation.

Overall, oral F/TDF PrEP was well tolerated by participants across all study groups. There was no grade 3 or higher adverse event observed during the DOD phase of the study. However, one grade 3 adverse event was reported in a participant 4 days after they had completed the study drug dosing phase (i.e., 4 days post-DOD). This adverse event was due to hospitalization with a laboratory-confirmed hepatitis A infection but was deemed to be unrelated to the study product. There were two grade 2 adverse events reported during the dosing period, one in the 4 doses/week arm, and the other in the 2 doses/week arm. Overall, 73 grade 1 adverse events occurred, 50% in daily dosing and about 25% for each in the non-daily dosing groups. Grade 1 adverse events most frequently reported included nausea, vomiting, and stomach pain. No HIV infection occurred during the study period.

### TFV-DP dose proportionality

**Fig 2:**
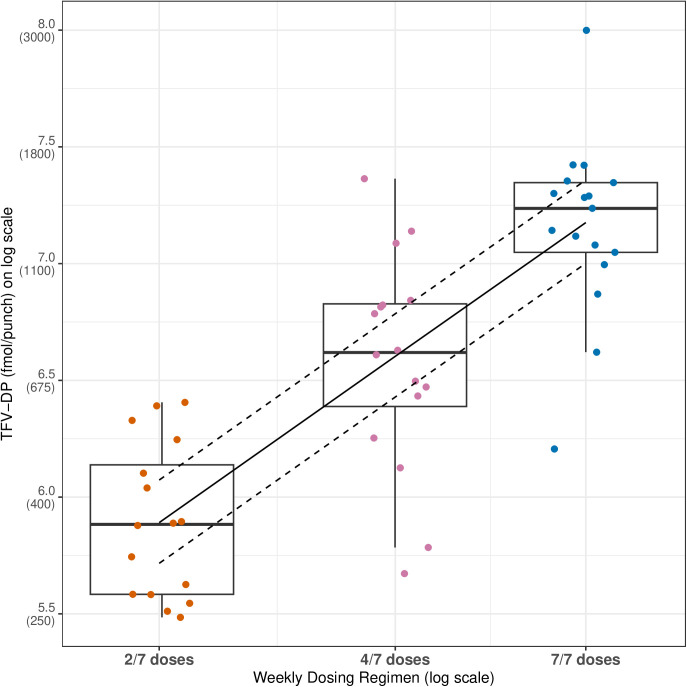
Tenofovir diphosphate (TFV-DP) dose proportionality in dried blood spots. Dose proportionality was determined based on the power model using observed week eight TFV-DP concentrations. Dose proportionality was assumed if the 90% confidence interval for the log (dose) coefficient was contained within the limits (0.8, 1.25). On the y-axis, natural TFV-DP levels are shown in parentheses below the logarithmic scale. The central box line represents the median, the box represents the interquartile range, and the whiskers represent 1.5 times the interquartile range from the quartiles. Longer whiskers indicate greater variability in the data, while shorter whiskers indicate less variability.

Overall, 1790/1792 (99.9%) of all expected doses were directly observed: 1,656 in-person, 134 by real-time video streaming. The proportion of doses observed via real-time video streaming by study arm was 5.3%, 5.1%, and 9.4% for 2, 4, and 7 doses/week, respectively. The two missed doses were from two separate individuals in the daily dosing arm; one individual missed the dose during week 6 dosing, and the second individual missed the dose during week 7 dosing. Dose proportionality was assessed using week 8 observed DBS TFV-DP concentrations. Overall, DBS TFV-DP concentrations varied in direct proportion to the dosing frequency with an estimated slope (90%CI) of 1.02 (0.84, 1.20) (Fig 2).

### Observed TFV-DP and FTC-TP concentrations in DBS and PBMCs

**Fig 3:**
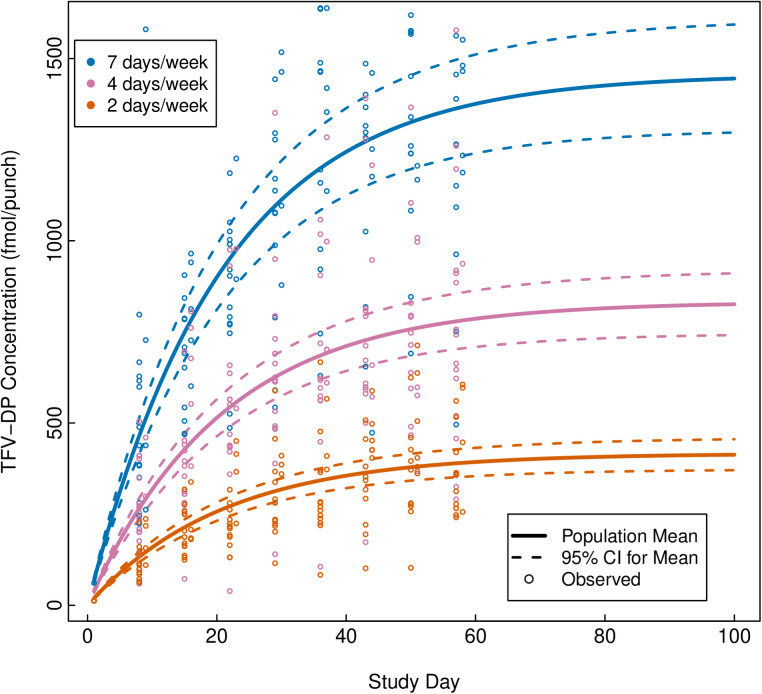
Observed and fitted tenofovir diphosphate (TFV-DP) concentrations in dried blood spots (DBS) by dosing week. Steady-state TFV-DP concentrations in DBS for varying dosing frequency were estimated using one-compartment nonlinear mixed effects population pharmacokinetic models using all available concentrations across all dosing time points. Solid lines represent fitted means for each group, while dotted lines represent 95% confidence intervals around the mean by dosing arms. Circles represent observed data by dosing arm.

**Fig 4:**
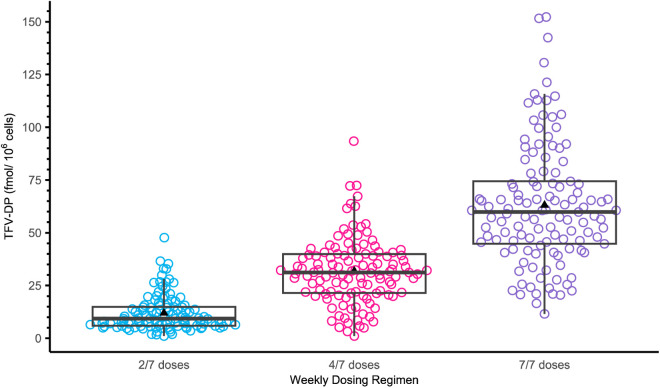
Observed steady-state tenofovir diphosphate (TFV-DP) concentrations in peripheral blood mononuclear cells (PBMCs) by dosing arm. Observed quantitative concentrations of TFV-DP in PBMCs by dosing arm. Steady-state samples were collected after 2, 3, 4, 5, 6, 7, and 8 weeks of directly observed dosing. A total of 17 participants in 2 doses/week, 18 participants in 4 doses per week, and 18 participants contributed 113, 118, and 121 steady-state samples, respectively. Individuals could contribute multiple steady-state samples. The central box line represents the median, the box represents the interquartile range, and the whiskers represent 1.5 times the interquartile range from the quartiles. Longer whiskers indicate greater variability in the data, while shorter whiskers indicate less variability. The black triangle shape represents the mean.

The observed DBS TFV-DP concentrations-time profile are presented in Fig 3 and PBMCs TFV-DP concentrations in Fig 4, by study group. Overall, the observed median (IQR) week 8 TFV-DP concentrations in DBS were 359 (266–464), 749 (596–923), and 1,389 (1,151–1,551) fmol/punch after 2, 4, and 7 doses/week, respectively (Table 2). At week 8, DBS FTC-TP was quantifiable in 71%, 19% and 0% of the samples in daily, 4, and 2 doses/week groups, respectively. Similarly, the observed mean (SD) steady-state TFV-DP concentrations in PBMCs were 11.99 ± 8.47, 31.81 ± 15.66, and 63.1 ± 28.97 fmol/10^6^ cells after 2, 4, and 7 doses/week, respectively (Table 3 and Fig 4). Mean (SD) FTC-TP in PBMC was 1306.19 ± 2079.11, 2601.6 ± 1774.33, 5628.35 ± 2427.34 fmol/10^6^ cells after 2, 4, and 7 doses per week, respectively (Table 3). Of note, the observed mean (SD) TFV-DP concentrations in PBMCs after three daily DOT dosing were 48.03 ± 47.27 fmol/10^6^ cells and 56.12 ± 34.96 fmol/10^6^ cells, respectively.

**Table 2:** Observed TFV-DP and FTC-TP concentration in DBS (70:30) at week 8, by study arm.

	2 doses/week	4 doses/week	7 doses/week
**TFV-DP (fmol/punch)**			
*N*	16	16	17
Range	241–605	291–1,578	496–2,979
Mean ± SD	381 ± 129	794 ± 345	1,375 ± 523
Median (IQR)	359 (266–464)	749 (596–923)	1,389 (1,151–1,551)
Coefficient of variation	34%	43%	38%
FTC-TP (fmol/punch)*			
*N*	16	16	17
*n* (%) with quantifiable levels	0 (0%)	3 (19%)	12(71%)

Observed quantitative concentrations of tenofovir diphosphate (TFV-DP) and emtricitabine triphosphate (FTC-TP) in dried blood spots (DBS) for samples collected after 8 weeks of dosing. Point estimates are summarized as means, median, and data dispersion as ranges, standard deviation (SD), and interquartile range (IQR). *Since most of FTC-TP was not quantifiable in DBS, only the proportion of samples with quantifiable FTC-TP levels is report.

**Table 3:** Observed steady-state TFV-DP and FTC-TP concentrations in PBMCs.

	2 Doses/week	4 Doses/week	7 Doses/week
**TFV-DP (fmol/10**^**6**^ **cells)**			
Number of participants	17	18	18
Number of steady-state samples	113	118	121
*N* (%) samples unquantifiable levels	8 (7.1%)	3 (2.5%)	0 (0%)
Range	1.06–47.70	1.07–93.42	11.55–152.25
Mean ± SD	11.99 ± 8.47	31.81 ± 15.66	63.10 ± 28.97
Median (IQR)	9.27 (5.97–14.85)	31.21 (21.48–39.85)	59.83 (44.74–74.39)
**FTC-TP (fmol/10**^**6**^ **cells)**			
Number of participants	17	18	18
Number of steady-state samples	113	118	121
*N* (%) samples unquantifiable levels	4 (3.5%)	2 (1.7%)	0 (0%)
Range	19.2–12027.9	12.8–8912.4	421.2–13629.7
Mean ± SD	1306.2 ± 2079.1	2601.6 ± 1774.3	5628.6 ± 2427.3
Median (IQR)	483.0 (325.2–812.6)	2098.9 (1444.1–3279.7)	5431.3 (3976.7–7002.7)

Observed quantitative concentrations of tenofovir diphosphate (TFV-DP) and emtricitabine triphosphate (FTC-TP) in peripheral blood mononuclear cells (PBMCs) by dosing arm. Summary estimates were conducted on steady-state samples collected after 2, 3, 4, 5, 6, 7, and 8 weeks of directly observed dosing. Estimates are summarized as means, median, and data dispersion as ranges, standard deviation (SD), and interquartile range (IQR). Unquantifiable drug levels were replaced with half of the lower limit of quantification. The PBMC assay quantifiable linear range was 5–6,000 fmol/sample for TFV-DP and 0.1–200 pmol/ sample (100–200,000 fmol) for FTC-TP.

### Fitted steady-state TFV-DP concentrations in DBS and adherence threshold interpretations

**Fig 5:**
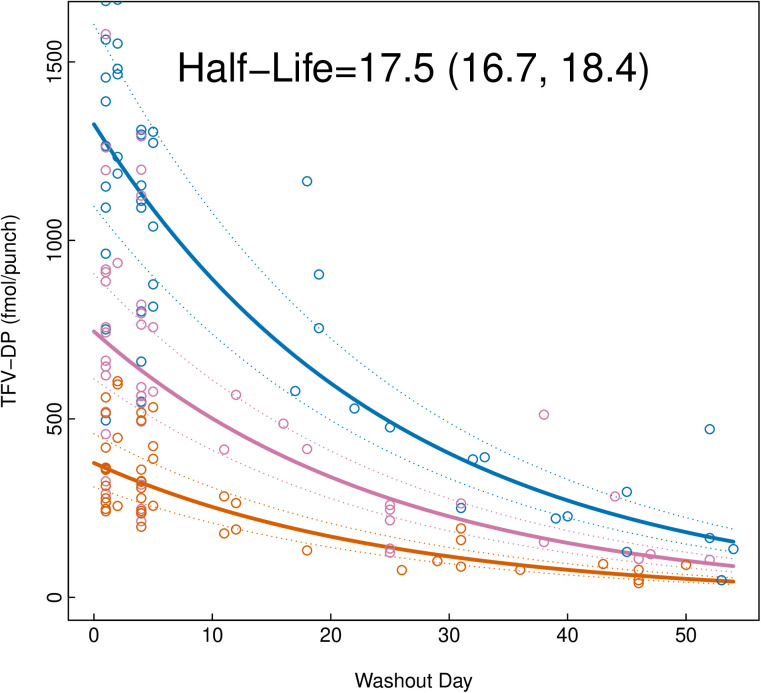
Tenofovir diphosphate exponential decay curve in dried blood spots (DBS). Mean half-life in days and 95% confidence interval for TFV-DP in DBS was estimated using one-compartment nonlinear mixed effects population pharmacokinetic models based on first-order kinetics using all available concentrations collected during the post-directly observed dosing decay period (wash out).

The fitted TFV-DP concentration-time profile in DBS by dosing group is presented in Fig 3. TFV-DP exhibited exponential decay kinetics in DBS, defined by mean half-life of 17.5 days (95%CI: 16.7, 18.4), consistent with previous studies (Fig 5). Overall, the fitted median (IQR) steady-state TFV-DP concentration in DBS was 416 fmol/punch (316, 516), 832 fmol/punch (631, 1,033), and 1,457 fmol/punch (1,106, 1,808) for 2, 4, and 7 doses/week, respectively (Table 4). Sensitivity analysis, which excluded TFV-DP concentrations derived from the seven replacement DBS cards, did not meaningfully affect the final steady-state estimates. Overall, age, weight, hematocrit, and estimated creatinine clearance, when assessed separately, were not statistically meaningfully associated with TFV-DP concentrations in DBS following F/TDF dosing (S1 Table).

**Table 4:** Fitted steady-state TFV-DP concentration thresholds in DBS for African cisgender women (fmol/punch).

Parameter	Dosing arm	Median	25th Percentile	75th Percentile
Asymptote	7 doses/week	1,457	1,106	1,808
Asymptote	4 doses/week	832	631	1,033
Asymptote	2 doses/week	416	316	516

Fifty-three participants contributed data to this analysis. Estimates are based on nonlinear mixed effects one-compartment model using all available concentrations in the dosing phase. The model assumes normal distribution for parameter estimates and ignores within (residual) for Asymptotes. Median and interquartile range were estimated using fitted values for each group with random effects.

### TFV-DP adherence interpretations according to DBS extraction method

**Fig 6:**
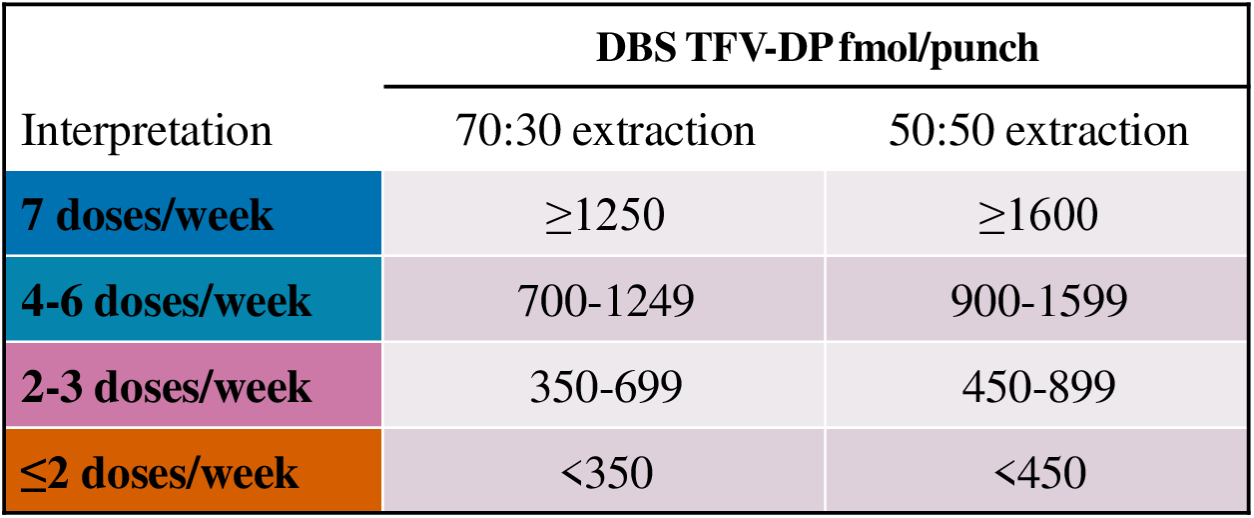
Updated tenofovir diphosphate (TFV-DP) adherence benchmarks after oral F/TDF PrEP according to the dried blood spots (DBS) extraction method for nonpregnant persons. TFV-DP adherence benchmarks following F/TDF PrEP in DBS based on the rounded 25th percentile of fitted steady-state concentrations for nonpregnant populations according to the DBS extraction method. The original adherence benchmarks were generated by the Colorado Antiviral Pharmacology Laboratory using the 70% methanol and 30% water (70:30) extraction process for DBS, but this extraction was revalidated by the same laboratory using a 50% methanol and 50% water (50:50) extraction method which yields 1.27-fold higher (95% CI 1.25, 1.28) drug recoveries compared to the 70:30 extraction process. We recommend that future studies and interpretations use the updated adherence benchmark thresholds based on the respective extraction reported by the testing laboratory. No sex-specific interpretation is recommended.

A total of 396 DBS samples were included in the parallel analysis to assess the relative efficiency of extracting TFV-DP and FTC-TP from DBS using 50:50 versus 70:30 extraction process. Overall, the 50:50 extraction process resulted in 1.27 (95% CI 1.25, 1.28) higher TFV-DP concentrations compared to the 70:30 extraction process (S2 Fig). The conversion factor of 1.27 was then applied to the previous 70:30 TFV-DP benchmarks to produce the following adherence interpretation benchmarks based on the 50:50 extraction: <450 for <2 dose/week, 450–899 for 2–3 doses/week, 900–1,599 for 4–6 doses/week, and ≥1,600 fmol/punch for 7 doses/week (Fig 6).

## Disscusion

This study investigated the relationship between directly observed oral F/TDF dosing and the resulting intracellular TFV-DP concentrations in DBS and PBMCs in African women. We have demonstrated that the TFV-DP thresholds derived from African women are similar to the original TFV-DP estimates derived from US-based participants. For example, 2, 4, and 7 doses/week from this study yielded DBS TFV_DP concentrations of 416 fmol/punch (316, 516), 832 fmol/punch (631, 1,033), and 1,457 fmol/punch (1,106, 1,808), respectively, whereas values from the US-based study yielded 416 fmol/punch (347, 498), 856 fmol/punch (714, 1,026), and 1,534 fmol/punch (1,280, 1,837). These data validate the use of the original adherence benchmarks to interpret adherence to F/TDF PrEP for cisgender women (Fig 6).

When used with sufficient adherence, oral F/TDF PrEP is a highly effective biomedical strategy to prevent against HIV acquisition in diverse populations, including women [21]. However, due to negative results in two of the first-generation randomized clinical trials of oral PrEP among women [22,23], several hypotheses questioned whether the pharmacology of oral F/TDF PrEP may be different in cisgender women, especially among young women in Africa, compared to what is already established in men [24–26]. At the root of this controversy has been the lack of robust pharmacologic data on dosing thresholds and expected TFV-DP concentrations in DBS for cisgender women. Our data demonstrate that the expected TFV-DP concentrations attained in African women following F/TDF PrEP are not meaningfully different from those previously defined for US-based participants that included both men and women. Specifically, the medians and IQRs were all within 15% of the original TFV-DP adherence estimates based on the DOT-DBS study [4]. Additionally, the decay half-life was 17.5 days in this study compared to 17 days in the DOT-DBS study.

The DOT-DBS benchmarks were an average estimate from both males and females from the US, but subgroup analysis indicated that TFV-DP concentrations in DBS were ~17.6% lower in males than in females [4]. Contrary to our expectation, we found that the steady-state TFV-DP estimates among women from our study more closely overlapped and were nearly identical to estimates reported for the subgroup of US men participants than they were to concentrations from the U.S female participants in the DOT-DBS study (i.e., median TFV-DP concentrations for 2, 4, and 7 doses per week, 375, 774, and 1,389 fmol/punch for males in DOT-DBS versus 416, 832, and 1,457 fmol/punch for women in our study; and 455, 939, and 1,685 fmol/punch for females from the DOT-DBS study). Taken together, even with these small differences, concentration in subgroups by sex were within 14% of the estimates in our study, indicating that the original benchmarks can be applied to appropriately interpret adherence and oral F/TDF PrEP implementation in cisgender women. Of note, the referenced DBS TFV-DP benchmark thresholds were generated based on the rounded 25th percentile cutoffs, which means that in a sample of individuals using a particular dosing frequency, concentrations from ~25% of the sample population will inherently be below the respective benchmark threshold. For example, approximately 25% of steady-state DBS TFV-DP concentrations from a sample of individuals using an average of 4 doses/week in the preceding 8–12 weeks would be expected to be below the 700 fmol/punches threshold, and those individuals would typically be misclassified as not having optimal adherence based on this cutoff threshold. In this study, we found that the coefficient of variation in the observed TFV-DP concentrations in DBS ranged between 34% and 43% across dosing frequencies, and in general, a given concentration could be generated by different adherence (or nonadherence) patterns, which may not be fully captured by the defined cutoff thresholds. Taken together, this implies that prevention benchmark thresholds are useful and critical in clinical and programtic PrEP research to help interpret the average population-level adherence, but individualized interpretation and client-level adherence feedback should be done with caution and within the broader clinical and program context.

For HIV prevention, drug concentrations in PBMCs, the pharmacologically active site, provide the closest surrogate for the levels associated with HIV protection. PBMC levels from the STRAND study, when applied to the iPrEx clinical cohort, helped to define the effective TFV-DP concentration associated with HIV protection for men who have sex with men [5,10]. For women, there has been considerable scientific and public health debate on the adherence levels required to attain sufficient HIV protection [27–31]. On one hand, some clinical and pharmacodynamics modeling studies have suggested that women may need higher level of adherence to achieve the same HIV protection compared to men [27,31]. However, many of the reported estimates have been imprecise, with overlapping 95% confidence bound estimates for efficacy estimates between men and women [27,30,31]. On the other hand, recent clinical studies among African women have reported important levels of HIV protection with reasonable-but-imperfect PrEP adherence [32,33]. For instance, in the HPTN 082 study among young African women at substantial HIV risk [33,34], the median TFV-DP concentration was 485 fmol/punch representing an average dosing of 2–3 doses/week and only 25% had DBS TFV-DP levels that suggest “optimal adherence” based on the ≥700 fmol/punch threshold defined in the original benchmarks (70:30 extraction), which we now know were appropriate. Despite this low to moderate adherence in a population with high HIV risk, as defined in that study by a VOICE risk score of 7 (scores ≥5 have been associated with ≥6% HIV incidence in prior cohorts), the annual HIV incidence was 1% [33]. These findings have recently been validated by a large pooled analysis involving more than 6,000 women across 11 real-world studies, suggesting that women can achieve effective HIV protection if they maintain, on average, at least four oral F/TDF PrEP dosing per week [9]. Reassuringly, in our study, we found that TFV-DP concentrations in PBMCs were within range of levels observed from other studies among men and women [35]. Taken together, although our study does not provide direct evidence on the concentration–protection relationship in women, the data on TFV-DP concentration levels in PBMCs among women from our study provide important additional cross-validation of the clinical and pharmacodynamic modeling studies that have suggested no meaningful difference in the pharmacology of TFV-DP for HIV prevention in women versus men. Importantly, the recent clinical trials of PrEP in women [8] provide a tremendous opportunity to pool additional clinical data that link TFV-DP concentrations to HIV infections among women who used F/TDF PrEP in the trials that can help improve the precision in estimates of the intrinsic and achievable HIV protective efficacy.

Recently, the Colorado Antiviral Pharmacology Laboratory, the same laboratory that supported the testing for DOT-DBS study, validated a new 50% methanol and 50% water extraction process (50:50), which yields better TFV-DP recoveries. Our study provided a perfect opportunity to compare the extraction performances of 70:30 versus 50:50 using punches from the same DBS spot arising from a DOD study. We show that the two methods of DBS extraction are highly correlated and robust to varying dosing frequency and concentrations, with 1.27-fold higher drug recovery using the 50:50 process compared to the original 70:30 process. These data enabled us to establish a new TFV-DP interpretation table based on the 50:50 extraction method. We recommend that future studies and interpretations of adherence to oral F/TDF PrEP use the updated adherence benchmark thresholds (Fig 6) based on the respective extraction method and that the testing laboratories should report DBS extraction used.

The study has several strengths, including DOD, concentration assessment in both DBS and PBMCs, and using the same laboratory and assays that produced the original benchmarks from US-based studies. There are also limitations. First, dosing was up to 8 weeks, which is about 90% of TFV-DP steady-state in DBS. However, pharmacokinetic modeling enabled steady-state estimates. The one-compartment model assumed constant dose and estimated the range of steady-state TFV-DP concentrations associated with that dose. Second, given that assessment of drug concentrations in PBMCs is highly dependent on cell processing and count protocols, it is difficult to compare PBMC results across laboratories and studies. Third, the study focused on the adherence–concentration thresholds but did not explicitly evaluate the concentration–efficacy relationship. However, clear knowledge of the dosing–concentration relationship drawn from these data will help to inform more precise estimates for the intrinsic concentration and HIV protection relationship for cisgender women. Fourth, the study was among healthy volunteers, and no information on comorbidities and concomitant medication was assessed, which may limit generalizability to such individuals. However, based on the known pharmacology TFV-DP, the dominant factor that influences effective drug exposure is adherence, and typical PrEP users are young, healthy individuals with lower risk for comorbid conditions.

In summary, we have defined the expected TFV-DP concentrations from oral F/TDF PrEP dosing for cisgender women. We demonstrate that TFV-DP concentrations in DBS varied in direct proportion with adherence, and the resultant thresholds are similar to the original benchmark estimates derived from the US populations. These data validate the application of the original TFV-DP benchmarks to interpret adherence to oral F/TDF PrEP in HIV clinical and PrEP programs among women.

## Supporting information

S1 Fig Study schema for directly observed dosing and sampling schedule. Participants assigned the two-dose-per-week arm were dosed on Mondays and Tuesdays. Participants assigned to the four dosing arms were dose on Mondays, Tuesdays, Thursdays, and Fridays. Participants assigned 7 doses per week were dosed daily. Doses were directly observed in person or via live video streaming on WhatsApp. Blood for whole blood (WB), dried blood spots (DBS), and peripheral blood mononuclear cells (PBMCs) was collected in EDTA tube at day 4 and day 7 after the first directly observed dose and then weekly thereafter during 8 weeks of dosing. For the washout phase, each participant had at least two post-dose visits, with the first visit occurring within 48 hours and the second visit was staggered any time between 2 and 8 weeks after the last DOD. All sampling visits were scheduled by convenience without regard to time or day since the last dose.
(TIFF)

S2 Fig Comparison of the 70:30 and 50:50 DBS extraction methods. Tenofovir diphosphate (TFV-DP) quantitative concentration yield from dried blood spots (DBS) using the original 70% methanol and 30% water (70:30) extraction process and the validated new 50% methanol and 50% water extraction process (50:50) by dosing arm (color). Linear regression on the logarithmic scale was used to compare the drug recovery performance of the two extraction methods from paired 3-mm punch samples from the same DBS spot.
(TIF)

S1 Table Baseline predictors of tenofovir diphosphate (TFV-DP) levels in dried blood spots for non-pregnant persons. (DOCX)

S1 File CONSORT checklist. This checklist is licensed under the Creative Commons Attribution 4.0 International License (CC BY 4.0; https://creativecommons.org/licenses/by/4.0/).
(DOCX)

S2 File Study protocol. (PDF)

S3 File Statistical analysis plan. (PDF)

## Abbreviations

DBS: dried blood spots
DOD: directly observed dosing
F/TDF: emtricitabine/tenofovir disoproxil fumarate
FTC-TP: emtricitabine triphosphate
IQR: interquartile range
PBMC: peripheral blood mononuclear cell
PrEP: preexposure prophylaxis
TFV-DP: tenofovir diphosphate
