# Supplementary figures and images for: Adherence thresholds for emtricitabine-tenofovir disoproxil fumarate preexposure prophylaxis against HIV acquisition in cisgender women: A randomized directly observed dosing study
Source: PLoS Med. 2025 Sep 9;22(9):e1004732. doi: 10.1371/journal.pmed.1004732 (PMC12435667; doi:10.1371/journal.pmed.1004732)

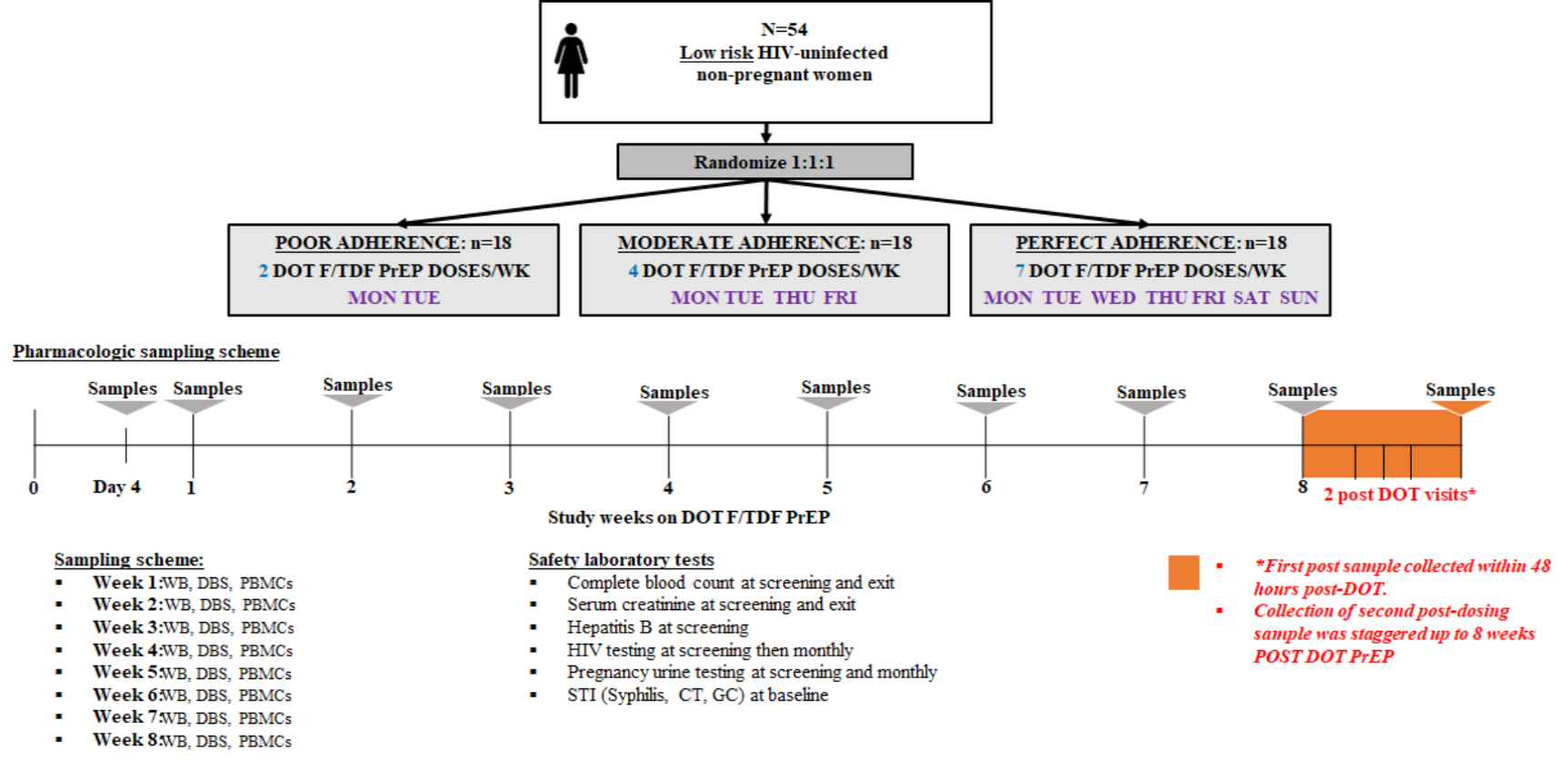

Supplement: S1 Fig — Participants assigned the two-dose-per-week arm were dosed on Mondays and Tuesdays. Participants assigned to the four dosing arms were dose on Mondays, Tuesdays, Thursdays, and Fridays. Participants assigned 7 doses per week were dosed daily. Doses were directly observed in person or via live video streaming on WhatsApp. Blood for whole blood (WB), dried blood spots (DBS), and peripheral blood mononuclear cells (PBMCs) was collected in EDTA tube at day 4 and day 7 after the first directly observed dose and then weekly thereafter during 8 weeks of dosing. For the washout phase, each participant had at least two post-dose visits, with the first visit occurring within 48 hours and the second visit was staggered any time between 2 and 8 weeks after the last DOD. All sampling visits were scheduled by convenience without regard to time or day since the last dose. (TIFF) [file pmed.1004732.s001.tiff]

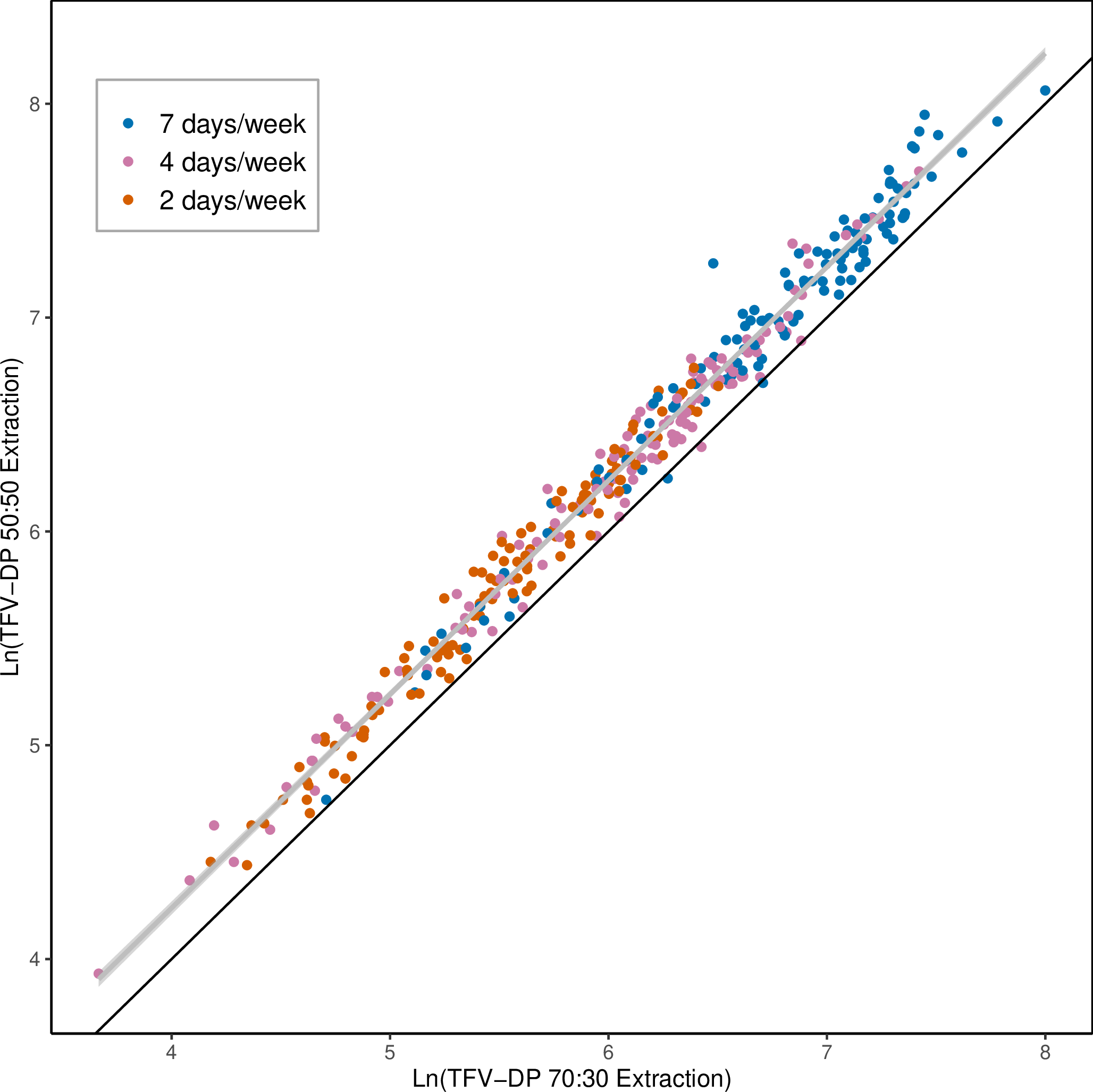

Supplement: S2 Fig — Tenofovir diphosphate (TFV-DP) quantitative concentration yield from dried blood spots (DBS) using the original 70% methanol and 30% water (70:30) extraction process and the validated new 50% methanol and 50% water extraction process (50:50) by dosing arm (color). Linear regression on the logarithmic scale was used to compare the drug recovery performance of the two extraction methods from paired 3-mm punch samples from the same DBS spot. (TIF) [file pmed.1004732.s002.tif]
