# Supplementary material for: Adherence thresholds for emtricitabine-tenofovir disoproxil fumarate preexposure prophylaxis against HIV acquisition in cisgender women: A randomized directly observed dosing study
Source: PLoS Med. 2025 Sep 9;22(9):e1004732. doi: 10.1371/journal.pmed.1004732 (PMC12435667; doi:10.1371/journal.pmed.1004732)
Supplement: S1 Table — (DOCX) [file pmed.1004732.s003.docx]

**Table S1: Baseline predictors of tenofovir diphosphate (TFV-DP) levels in dried blood spots for non-pregnant persons.**

|  | **Estimate** | **Lower CI** | **Upper CI** | **p-value** |
| --- | --- | --- | --- | --- |
| Weight | -0.75% | -1.58% | 0.10% | 0.087 |
| Age | -1.92% | -4.72% | 0.97% | 0.1966 |
| Hematocrit | 0.98% | -1.91% | 3.96% | 0.5131 |
| Creatinine Clearance | 0.05% | -0.09% | 0.19% | 0.4952 |

Fit using a mixed-effects model with b-spline transformation of study day and dosing regimen. The model included all available concentration including from post-dosing visits. Outcome was fit on a long scale and the back-transformed estimates above represent a percent change in TFV-DP concentrations for a one unit change in the predictor. Example after adjusting for age, hematocrit and creatinine clearance, TFV-DP concentrations, on average, decreased by 0.75% for every 1 kg increase in weight. This difference was not significantly different than zero (p=0.087). Creatinine clearance was estimated with Cockroft Gualt equation.
