# Supplementary material for: Adherence thresholds for emtricitabine-tenofovir disoproxil fumarate preexposure prophylaxis against HIV acquisition in cisgender women: A randomized directly observed dosing study
Source: PLoS Med. 2025 Sep 9;22(9):e1004732. doi: 10.1371/journal.pmed.1004732 (PMC12435667; doi:10.1371/journal.pmed.1004732)
Supplement: S2 File — (PDF) [file pmed.1004732.s005.pdf]

STUDY PROTOCOL

Pharmacology of TDF-FTC Pre-exposure Prophylaxis in Kenyan Cisgender women

The Women TDF-FTC Benchmark Study

Funded by:
Division of AIDS, National Institute of Allergy and Infectious Diseases

(R01AI155086)

Protocol Chair:
Kenneth K. Mugwanya, MBChB, MS, PhD.

University of Washington
Seattle, USA

Protocol Co-Chair:
Peter L. Anderson, Pharm.D.

University of Colorado, Denver, USA

DAIDS-ES ID: 38807

ClinicalTrials.gov: *Pending*

IND Status: IND Exempt #155505

Version 1.0

20May, 2021

The Women TDF-FTC Benchmark Study

Pharmacology of TDF-FTC Pre-exposure Prophylaxis in Kenyan Cisgender women

TABLE OF CONTENTS

LIST OF ACRONYMS 6

PROTOCOL TEAM ROSTER 8

PROTOCOL SIGNATURE PAGE 10

PROTOCOL SCHEMA 11

STUDY SUMMARY 13

PROTOCOL ABSTRACT/SUMMARY 13

LAY SUMMARY 14

CONSORT DIAGRAM 15

1 INTRODUCTION 16

1.1 Background and prior studies 16

1.1.1 Disproportionate risk for HIV among African cisgender women 16

1.1.2 Evidence of TDF/FTC PrEP efficacy and relationship to adherence 16

1.1.3 Safety of TDF/FTC PrEP in pregnancy 17

1.2 Rationale 17

1.2.1 Adherence-concentration- thresholds for TDF/FTC PrEP 17

1.2.2 Pharmacology of TFV and TFV-DP in African cisgender women. 18

1.2.3 Pharmacokinetic TDF/FTC PrEP Adherence in Pregnancy 19

1.3 Problem statement 20

1.4 Justification 20

2 STUDY OBJECTIVE AND DESIGN 20

2.1 Overall Hypothesis 20

2.2 Overall Objective 20

2.3 Primary Objectives 21

2.4 Secondary Objectives 21

2.5 Study Design 21

2.5.1 Study design for non-pregnant cisgender women 21

2.5.2 Study design for pregnant cisgender women 22

2.6 Enrollment Targets 22

3 STUDY POPULATION 23

3.1 Inclusion Criteria 23

3.2 Exclusion Criteria 24

3.3 Study Setting 25
3.4 Recruitment and Prescreening Process 25
3.5 Co-Enrollment Guidelines 25
3.6 Retention of Study Participants 26
3.7 Participant Withdrawal 26
3.8 Replacement of Study Participants 27
4 STUDY PRODUCT 27
4.1 Study Product Formulation 27
4.1.1 Study product storage 27
4.2 Source of Study Product and Accountability 27
4.2.1 Study product dispensation 27
4.2.2 Return and Destruction of Study Product 28
4.3 Assessment of Participant Adherence with Study Product/Intervention 28
4.4 Concomitant Medications 28
4.5 Prohibited Medications 28
4.6 Precautionary Medications 28
4.7 Toxicity Management 28
4.8 HIV Seroconversion 28
4.9 Dose Modification 29
5 STUDY PROCEDURES 29
5.1 Screening Procedures 29
5.2 Enrollment and Follow up Procedures 30
5.2.1 Dosing schedule and assessment of compliance 30
5.2.2 Protocol pharmacology sampling visit schedule 31
5.2.3 Clinical Evaluations/Procedures 34
5.3 Procedures for participants identified as HIV infected 35
5.4 Procedures for follow up and care for pregnant cisgender women after DOT PrEP 36
6 SAFETY MONITORING AND ADVERSE EVENT REPORTING 36
6.1 Safety Monitoring 36
6.2 Clinical Data Review 37
6.3 Adverse Event Definition and Reporting 37
6.4 Relationship to Study Product 37
6.5 Grading Severity of Events 38
6.6 Serious Adverse Event (SAE) 38
6.7 Discontinuation of Study Medication 38

6.8 Discontinuation of study drug with toxicity 38
6.8.1 AE Grades 1 or 2 39
6.8.2 AE Grade 3 39
6.8.3 Grade 4 39
6.8.4 Creatinine Clearance 39
6.9 Expedited Adverse Event (EAE) Reporting to DAIDS 39
6.10 Reporting Requirements for this Study 39
6.11 Social Impact Reporting 40
7 STATISTICAL CONSIDERATIONS 40
7.1 Review of Study Design 40
7.2 Study endpoints 40
7.3 Data Analysis 40
7.3.1 Primary outcomes 40
7.3.2 Secondary outcomes 41
7.4 Sample Size Considerations 42
7.5 Randomization and Blinding Procedures 42
7.6 Planned Interim Analyses 42
8 HUMAN SUBJECTS CONSIDERATIONS 43
8.1 Ethical Review 43
8.2 Informed Consent 43
8.3 Voluntary Participation 44
8.4 Potential Risks to Human Subjects 44
8.5 Approaches to Minimize Risks 46
8.6 Benefits. 47
8.7 Treatment for Injury 48
8.8 Study Records 48
8.9 Confidentiality 48
8.10 Reimbursement 48
8.11 Communicable Disease Reporting Requirements 49
8.12 Reports of Sexual Abuse 49
8.13 Study Discontinuation 49
9 LABORATORY SPECIMENS AND BIOHAZARD CONTAINMENT 49
9.1 Laboratory evaluations and specimen collections 49
9.2 Specimen preparation, handling, and shipping 50
9.3 Specimen Storage and Possible Future Research Testing 50

9.4 Quantification of drug concentrations ... 50
9.5 Biohazard Containment ... 50
10 ADMINISTRATIVE PROCEDURES ... 51
10.1 Protocol Registration ... 51
10.2 Study activation ... 51
10.3 Study coordination ... 51
10.3.1 Data storage and management responsibilities ... 51
10.3.2 Source Documents and Access to Source Data/Documents ... 52
10.3.3 Data monitoring ... 52
10.3.4 Quality control and Quality assurance ... 52
10.4 Study monitoring ... 52
10.5 Protocol Compliance ... 53
10.6 Investigator's Records ... 53
10.7 Use of Information and Publications ... 54
10.8 Resource sharing and dissemination plan ... 54
10.8.1 Resource Sharing: ... 54
10.8.2 Data Sharing Plan ... 54
10.8.3 Collaborative research ... 55
10.8.4 Dissemination plan ... 55
10.8.5 Presentations ... 55
10.9 Intellectual property consideration ... 55
11 TIMELINES ... 56
12 EXPECTED APPLICATION OF RESULTS ... 56
13 STUDY LIMITATIONS ... 56
14 REFERENCES ... 57
15 APPENDIXES— STUDY CONSENT FORM TEMPLATES ... 64
Study Screening Informed Consent Form ... 64
Study Enrollment Informed Consent Form — Non-Pregnant Cisgender Women ... 70
Enrollment Informed Consent Form — Pregnant Cisgender Women ... 80

LIST OF ACRONYMS

AE	Adverse Event
ART	Anti-Retroviral Therapy
BMI	Body Mass Index
CAB	Community Advisory Board
CBC	Complete Blood Count
CDC	Centers for Disease Control and Prevention
CLIA	Clinical Laboratory Improvement Amendments
CrCl	Creatinine Clearance
CRFs	Case Report Forms
Css	Drug concentration at steady state
CV	Coefficient of Variation (SD/Mean)
DAIDS	Division of Acquired Immunodeficiency Syndrome
DBS	Dried Blood Spots
DNA	Deoxyribonucleic acid
DOT	Directly Observed Therapy
EAE	Expedited Adverse Event
FDA	Food and Drug Administration
FTC	Emtricitabine
FTC-DP	Emtricitabine-diphosphate
GCP	Good Clinical Practices
GEMS	Global Evaluation of Microbicide Sensitivity
GFR	Glomerular Filtration Rate
GT	Gonorrhea Trichomoniasis
HBAg	Hepatitis B Antigen
HBV	Hepatitis B Virus
HCT	Hematocrit
hDNA	Human Deoxyribonucleic acid
HIV	Human Immunodeficiency Virus
IEC	Information, Education and Communication
IoR	Investigator of Record
IRB	Institution Review Board

KEMRI	Kenya Medical Research Institute
MEMS	Medication Event Monitoring System
MOH	Ministry of Health
MSM	Men Who have Sex with Men
NASCOP	National AIDS & STI Control Programme
NIAID	National Institute of Allergy and Infectious Diseases
NIH	National Institutes of Health
OHRP	Office for Human Research Protections
PBMCs	Peripheral Blood Mononuclear Cells
PI	Principal Investigator
PK	Pharmacokinetics
PMTCT	Prevention of mother-to-child transmission
PPB	Pharmacy and Poisons Board
PrEP	Pre-exposure prophylaxis
PSRT	Protocol Safety Review Team
RBC	Red Blood Cells
RSC	Regulatory Support Center
SAE	Serious Adverse Event
SOP	Standard Operating Procedure
SSP	Study Specific Procedures
STI	Sexually Transmitted Infection
SUSAR	Suspected Unexpected Serious Adverse Reaction
TAF	Tenofovir Alafenamide Fumarate
TDF	Tenofovir Disoproxil Fumarate
TDF/FTC	Truvada® or Tenofovir Disoproxil Fumarate/Emtricitabine
TFV	Tenofovir
TFV-DP	Tenofovir-diphosphate
TP	Triphosphate
UC	University of Colorado
UW	University of Washington
WB	Whole blood
WHO	World Health Organization
βHCG	Beta-Human chorionic gonadotropin

The Women TDF-FTC Benchmark Study

Pharmacology of TDF-FTC Pre-exposure Prophylaxis in Kenyan Cisgender women

PROTOCOL TEAM ROSTER

Chair:
Kenneth Mugwanya, MBChB, MS, PhD
Assistant Professor, Global Health
University of Washington
325 Ninth Avenue; Box 359927
Seattle, WA 98104, USA
Phone:206-520-3886
Fax: 206 520 3831
Email: mugwanya@uw.edu

Co-Chair:
Peter L. Anderson, PharmD
Professor, Pharmaceutical Science
University of Colorado Denver
12850 E. Montview Blvd., V20-C238
Aurora, CO 80045, USA
Phone: 303 724 6128
Fax: 303 724 6135
Email: peter.anderson@ucdenver.edu

Adeola Adeyeye MD, MPA, LCDR
US Public Health Service
Deputy Branch Chief,
Clinical Prevention Research Branch,
Prevention Science Program, DAIDS/NIAID/NIH.
Room 8B36 MSC 9831,
5601 Fishers Lane, Rockville, MD 20852-
Phone:240.669.5005
Email: adeyeyeao@niaid.nih.gov
Role: NIAID Medical Officer

Usha Sharma, PhD, MPH
Prevention Sciences Program
DAIDS/NIAID/NIH
5601 Fishers Lane
Rockville, MD 20852
Phone: 240-292-4809
Phone: 301-219-8972 (cell)
Email- usharma@niaid.nih.gov
Role: NIAID Program Officer

Nelly R. Mugo, MBChB, M.Med, MPH.
Senior Principal Clinical Scientist, Kenya Medical Research Institute, Nairobi, Kenya
Research Associate Professor, University of Washington, Seattle, WA, USA
Phone:254 067 2222561;
Email: rwamba@uw.edu
Role: Site Principal Investigator and Protocol Obstetrician

Elizabeth Irungu, MBChB, MPH.
Research Scientist
Kenya Medical Research Institute, Nairobi, Kenya
Phone:2540672222561;
Email: eirungu@uw.edu
Role: Co-investigator

Deborah Donnell, PhD
Member, Fred Hutchinson Cancer Research Center, Seattle, WA, USA
Affiliate Professor, Department of Global Health, University of Washington, Seattle, WA, USA
Email: deborah@fredhutch.org
Role: Protocol Biostatistician

David Glidden, PhD
Professor, Epidemiology & Biostatistics
University of California San Francisco, San Francisco, CA, USA
Email: David.Glidden@ucsf.edu
Role: Protocol Biostatistician

Catherine Kiptinness, B. Pharm, MPH
Kenya Medical Research Institute, Nairobi, Kenya
Phone:254 067 2222561
Email: catherine@pipsthika.org
Role: Pharmacist of Record and Site Study Coordinator.

David Chege, BS. MLS, Dip. MLS
Clinical Trials Research Laboratory Kenyatta National Hospital/Univ. of Nairobi
Ground Floor Rm 7 and 8, Nairobi, Kenya
Phone: 254725206216
Email: dchege@pipsthika.org
Role: Laboratory Manager

Chohan, Bhavna MS, PhD
Clinical Assistant Professor, Global Health
University of Washington, Seattle, WA, USA
Clinical Trials Research Laboratory Kenyatta
National Hospital/Univ. of Nairobi
Ground Floor Rm 7 and 8, Nairobi, Kenya
Phone: 206-773-9901
Email: bchohan@u.washington.edu
Role: Laboratory Director

Justice Quame-Amaglo, MA
University of Washington
325 Ninth Avenue; Box 359927
Seattle, WA 98104, USA
Phone:206-520-3886
Fax: 206 520 3831
Email: quamaglo@uw.edu
Role: Research Manager

The Women TDF-FTC Benchmark Study

Pharmacology of TDF-FTC Pre-exposure Prophylaxis in Kenyan Cisgender women

Version 1.0 **May 20, 2021**

DAIDS-ES ID: 38807

ClinicalTrials.gov: *Pending*

IND Status: IND Exempt #155505

PROTOCOL SIGNATURE PAGE

Funded by:
Division of AIDS (DAIDS),
United States (US) National Institute of Allergy and Infectious Diseases (NIAID)
US National Institutes of Health (NIH)

I will conduct the study in accordance with the provisions of this protocol and all applicable protocol-related documents. I agree to conduct this study in compliance with United States (US) Health and Human Service regulations (45 CFR 46); applicable U.S. Food and Drug Administration regulations; standards of the International Conference on Harmonization Guideline for Good Clinical Practice (E6); Institutional Review Board/Ethics Committee determinations; all applicable in-country, state, and local laws and regulations; and other applicable requirements (e.g., US National Institutes of Health, Division of AIDS) and institutional policies.

____

Name of Site Investigator of Record

___________________________________ ___________________________________

Signature of Site Investigator of Record Date

The Women TDF-FTC Benchmark Study

Pharmacology of TDF-FTC Pre-exposure Prophylaxis in Kenyan Cisgender women

DAIDS-ES ID: 38807

PROTOCOL SCHEMA

Purpose: To define blood and tissue benchmark concentrations of tenofovir (TFV) and tenofovir diphosphate (TFV-DP) in Cisgender women using directly observed (DOT) oral Tenofovir Disoproxil Fumarate (TDF) /Emtricitabine (FTC) pre-exposure prophylaxis (PrEP). Data from this study will subsequently be applied to testing from archived samples from the Partners PrEP clinical trial to estimate the TFV-DP concentrations associated with HIV protection in cisgender women and the required adherence to achieve these concentrations. These findings will help in the interpretation of results obtained in HIV prevention trials and programs in cisgender women.

Study design: An open-label, randomized, three-arm, directly observed therapy, phase 2b pharmacologic study. HIV-uninfected non-pregnant cisgender women will be randomly assigned 1:1:1 to 1 of 3 dosing frequencies of DOT TDF/FTC PrEP: 2, 4, or 7 doses/week to help differentiate poor and modest from perfect dosing. An additional contemporaneous cohort of pregnant to receive daily dosing will also be recruited to evaluate the impact of pregnancy on blood and cellular drug levels. We will measure drug concentrations in blood, vaginal fluid, and tissue during the study.

Study population: The study will enroll two distinct but complementary cohorts of 18-30 years old HIV uninfected Kenyan cisgender women: 1) Up to 54 non-pregnant cisgender women at low risk of HIV and 2) Up to 18 pregnant cisgender women at elevated risk of HIV and willing to use PrEP.

Study site: The Kenya Medical Research Institute-Thika Research Site, Thika, Kenya

Study regimen: Volunteers will receive 200mg emtricitabine and 300mg of tenofovir disoproxil fumarate according to the dosing regimens outlined in the study design.

Study duration: The study will last for approximately 16 weeks (8 weeks of DOT PrEP and up to 8 weeks of post DOT PrEP monitoring) for each participant. Recruitment, enrollment, and follow up will continue for approximately 2 years.

Hypothesis: TDF/FTC dose-response thresholds from DOT PrEP dosing for cisgender women will be defined but will differ from thresholds defined for MSM populations; pregnant cisgender women will have lower benchmark concentrations in peripheral blood mononuclear cells (PBMCs).

Primary objectives:

1. To define the cisgender women-specific expected blood concentrations and dose-proportionality for TFV-DP in dried blood spots (DBS) and PBMCs using directly observed TDF/FTC therapy at 2, 4, 7 doses per week.
2. To establish a model to predict adherence rate to TDF/FTC by level of TFV-DP in DBS for cisgender women.

Secondary objectives:

1. To define the expected concentrations and dose-proportionally for TFV-DP in PBMCs and vaginal tissue

2. To quantify the effect of pregnancy on the benchmark concentrations of TFV, TFV-DP, FTC, and FTC-TP in pregnant cisgender women using daily DOT TDF/FTC PrEP
3. Examine relationships among drug concentrations in plasma, whole blood (WB), DBS, and PBMCs.
4. To determine the influence of biological variables (e.g. mean corpuscular volume and hematocrit, age, weight, sex) on drug concentrations.
5. Compare drug concentrations in DBS from fingerstick versus transferring blood from blood tubes.

Primary outcomes:

Benchmark concentrations of TFV-DP in DBS and PBMCs, and the dosing frequency (i.e., doses per week) required to generate them.

The Women TDF-FTC Benchmark Study

Pharmacology of TDF-FTC Pre-exposure Prophylaxis in Kenyan Cisgender women

STUDY SUMMARY

PROTOCOL ABSTRACT/SUMMARY

Pre-exposure prophylaxis (PrEP) using co-formulated emtricitabine (FTC) and tenofovir disoproxil fumarate (TDF) is a potent HIV prevention method for men and cisgender women, with its efficacy highly dependent on adherence. Pivotal studies that combined clinical epidemiology and pharmacology defined thresholds for PrEP protection in men who have sex with men (MSM) that have been key to PrEP promotion and development of new PrEP agents. For African cisgender women at risk for HIV, a priority group due to disproportionately high incident HIV infections, variable adherence in PrEP clinical trials and limited pharmacologic data have resulted in lack of clarity about levels of PrEP use required for HIV protection. Single-dose tissue pharmacology studies suggest oral TDF/FTC PrEP dosing delivers 20- to 100-fold higher TFV-DP in rectal tissue compared with vaginal tissue, suggesting cisgender women may need extraordinary perfection for HIV protection; on the other hand, in clinical studies in cisgender women with reasonable-but-imperfect PrEP adherence, suggest important levels of HIV protection. Thus, understanding the pharmacological-HIV protection relationship for cisgender women is essential. We will conduct an open-label, randomized, three-arm, directly observed pharmacological study of TDF/FTC PrEP in African cisgender women. The primary objectives: 1) To define the cisgender women-specific expected blood concentrations and dose-proportionality for TFV-DP in DBS and PBMCs using directly observed TDF/FTC therapy at 2, 4, 7 doses per week. 2) To establish a model to predict adherence rate to TDF/FTC by level of TFV-DP in DBS for cisgender women. HIV-uninfected non-pregnant cisgender women will be randomly assigned to 1 of 3 dosing frequencies of directly observed therapy (DOT) TDF/FTC PrEP: 2, 4, or 7 doses/week to help differentiate poor and modest from perfect adherence. An additional contemporaneous cohort of pregnant cisgender women to receive daily dosing will also be recruited to evaluate the impact of pregnancy on blood and cellular drug levels. The study will enroll two cohorts of 18-30 years old HIV uninfected cisgender women at Thika site in Kenya: 1) Up to 54 non-pregnant cisgender women at low risk of HIV and 2) Up to 18 pregnant cisgender women at elevated risk of HIV and willing to use PrEP. The proposed study will be the first to define TDF-PrEP adherence-blood concentration thresholds for African cisgender women, a priority population for HIV prevention. The findings will guide accurate interpretation of adherence and success of PrEP programs in cisgender women. This data will also help guide decisions on optimal PrEP dosing for HIV at-risk pregnant cisgender women in Africa.

The Women TDF-FTC Benchmark Study

Pharmacology of TDF-FTC Pre-exposure Prophylaxis in Kenya Cisgender women

LAY SUMMARY

Title: Pharmacology of TDF-FTC Pre-exposure Prophylaxis in Kenyan Cisgender women

Lay title: A project to define the expected blood levels of PrEP medications in cisgender women derived from varying number of PrEP doses taken per week.

Background:

African cisgender women are disproportionately affected with HIV and have elevated risk of acquiring HIV in pregnancy. Pre-exposure prophylaxis (PrEP) is an effective HIV prevention strategy, but variable adherence in PrEP clinical trials among African cisgender women and limited pharmacologic data have resulted in lack of clarity about the degree of PrEP use required for HIV protection in cisgender women. For men who have sex with men, the STRAND study defined the adherence levels to PrEP medication and expected drug concentrations arising from varying directly observed therapy (DOT) doses per week. Those thresholds are today being applied to studies of African cisgender women taking PrEP, and cisgender women-specific levels associated with HIV prevention have never been defined. However, recent data from large PrEP studies we have done among African cisgender women ─including Partners PrEP Study and Partners Demonstration Projects conducted at Thika site─, suggest those these levels may not reflect the pharmacology of cisgender women in African settings, in general and particularly in pregnancy. In these studies in African populations, PrEP dosing was not directly observed therapy and no study has established expected PrEP concentrations in African cisgender women with varying frequency of PrEP adherence. Thus, there is an urgent need to define the frequency of adherence and expected blood concentrations of PrEP medications and their relationship to HIV protection in non-pregnant and pregnant cisgender women.

What questions are we trying to answer?

We seek to define the expected blood levels of PrEP medications (tenofovir) for cisgender women taking directly observed oral PrEP therapy to understand the frequency of PrEP dosing associated with HIV protection in cisgender women. Cisgender women will be randomly assigned to receive varying frequency of weekly PrEP doses and followed for up to 16 weeks. We will also investigate how pregnancy affects the expected blood levels to help define optimal dosing of PrEP for HIV prevention during pregnancy. We will measure concentrations of PrEP medications (tenofovir and emtricitabine) in blood, urine, hair, and in vaginal fluid and tissue.

Where is the project taking place?

The study will take place in Thika, Kenya.

How will the project benefit society?

We propose to conduct the first study of oral PrEP adherence-concentration thresholds in Kenyan cisgender women, a population facing significant burden of incident HIV infection. Knowledge gained from the proposed study will include key blood concentrations of PrEP medications and the required frequency of pill taking to achieve those concentrations for cisgender women, and optimal dosing for PrEP in at-risk pregnant cisgender women. The results of this work will have immediate implications for PrEP programs and HIV prevention and global HIV prevention field.

CONSORT DIAGRAM

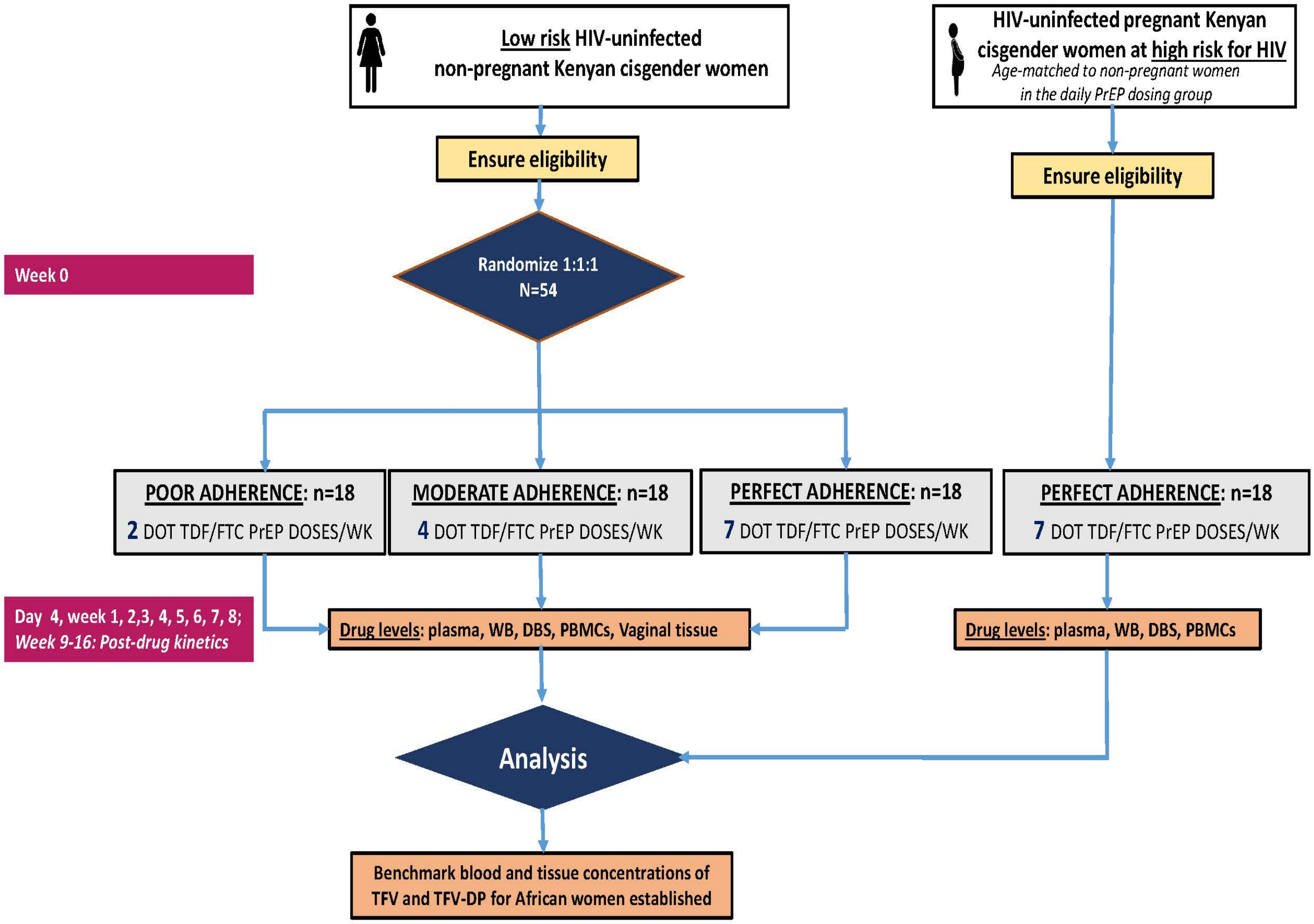

1 INTRODUCTION

1.1 Background and prior studies

Adherence is critical to therapeutic success for HIV treatment and pre-exposure prophylaxis of new HIV infections (PrEP). This section describes HIV risk in African cisgender women, TDF/FTC effectiveness and the importance of adherence, the lack of progress in quantifying adherence clinically, the high promise of TFV-DP in DBS as a novel adherence measure, and considerations for implementing adherence monitoring into clinical care.

1.1.1 Disproportionate risk for HIV among African cisgender women

More than 600 000 new infections occur annually among young African cisgender women[1], and the incidence rates are often double or more than their male age-mates[2-4]. At the same time, African cisgender women also face elevated risk of acquiring HIV in pregnancy and postpartum[5] with the risk of female HIV acquisition per condomless sex act as high as 3- and 4-fold during late pregnancy and in the postpartum[6], respectively, than non-pregnant period. A recent clinical trial in eastern and southern Africa (the ECHO Study)[7] that evaluated the risk of HIV acquisition in HIV-negative cisgender women who used three common contraceptive methods found that HIV incidence among sexually active cisgender women was alarmingly high ─ an average nearly 4% ─ with higher rates of HIV infection for cisgender women <25 years irrespective of the contraceptive method. Thus, these recent results have reminded us that HIV remains a significant personal risk and public health challenge for many cisgender women in African countries.

1.1.2 Evidence of TDF/FTC PrEP efficacy and relationship to adherence

PrEP is a highly efficacious and safe strategy for reducing HIV risk among men who have sex with men [8], heterosexual men and women[9, 10], and injection drug users[11] and now recommended as a prevention option for all persons at high risk for acquiring HIV[12-14]. Across first generation PrEP trials, there was variations in HIV protection (**Figure 1**)[15]. In the Partners PrEP Study, which included 1,785 East African women with a mutually disclosed HIV-infected partner, PrEP efficacy among cisgender women was 66% for TDF and 71% TDF/FTC PrEP vs placebo[9] and the protective effect was consistent in subgroups of cisgender women at high risk for HIV acquisition[16]. In the TDF2 study in Botswana, PrEP efficacy among cisgender women was 49% but was limited by the small sample size[10]. However, the enthusiasm for PrEP use in cisgender women faltered after FEM PrEP and VOICE trials failed to demonstrate efficacy[17, 18]. While there were initially several potential explanations for the failure of PrEP in these two trials including behavior and biologic factors, the consensus settled on adherence[19]. Beyond that, however, the absence of robust pharmacologic data for African cisgender women, particularly studies linking pharmacokinetics to pharmacodynamics, has resulted in lack of clarity about the degree of PrEP use and tenofovir concentrations required for HIV protection for cisgender women.

Figure 1. Graphical presentation how PrEP efficacy varied by adherence across PrEP trials (credit AVAC)

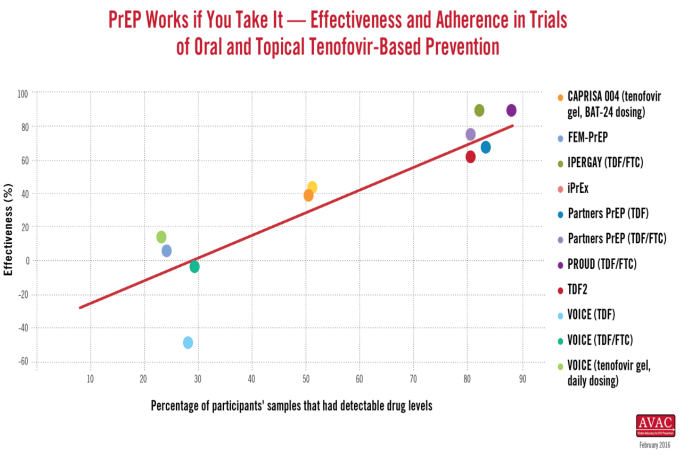

1.1.3 Safety of TDF/FTC PrEP in pregnancy

Data on PrEP safety can be extrapolated a number of studies including prevention of mother-to-child HIV transmission (PMTCT) and ART research, which includes TDF and FTC use in pregnancy [20-25]. In infants of HIV-infected women receiving TDF and FTC there has been limited signal of fetal risk – the lack of increased risk in this group with concurrent exposure to other ARVs and maternal HIV is reassuring [23]. The Antiretroviral Pregnancy Registry database includes data from 1982 infants exposed to TDF in first trimester in the US, without evidence of congenital anomalies [26]. There has been evidence of small growth deficits and decreased neonatal bone mineral content (5.3 gm lower) among infants receiving PMTCT-TDF, but clinical relevance of these findings is unknown [25, 27]. There is also a growing body of evidence from studies of conception among serodiscordant couples and PrEP RCTs that show no adverse events or difference in outcomes among infants born to mothers using PrEP at conception of during pregnancy [28-32].

In the Partners PrEP trial, women who became pregnant on PrEP had no evidence of adverse infant outcomes or long-term growth [28]. Among 431 women who became pregnant, pregnancy outcomes were similar between placebo and PrEP arms. Among 167 infants with serial growth assessment, there were no differences in weight or height z-scores or head circumference during infancy between infants within utero PrEP vs. placebo, However, women discontinued PrEP when pregnancy was diagnosed resulting in limited pregnancy time on PrEP. Additional data from the Partners Demonstration Project from 30 women who became pregnant ant elected to continue PrEP through pregnancy have been reassuring – with no increases in pregnancy loss, preterm birth, or congenital anomalies[33]. Finally, use of TDF in the last trimester of pregnancy to reduce hepatitis B vertical transmission is increasingly common, with no reports of an effect on pregnancy outcome to date. Encouragingly, in a randomized study in Thailand of tenofovir use among HIV-uninfected, hepatitis B-positive pregnant women (ClinicalTrials #NCT01745822) did not raise significant safety concerns[34, 35]. While safety data are generally reassuring, there is need for continued accrual of data on growth and pregnancy outcomes in PrEP research studies and demonstration projects. Breastfeeding is not a contraindication for maternal PrEP as minimal drug is likely to pass to infants, however, there are scant data from infants exposed to PrEP during lactation [36-38]. The level of safety evidence for PrEP (no animal fetal toxicity, thousands of infants exposed to TDF/FTC PMTCT, hundreds to PrEP in early pregnancy) led WHO, CDC, and the Kenyan Ministry of Health (MoH) to permit PrEP use in pregnancy [36, 39, 40]. WHO guidelines recommend PrEP for anyone at 'substantial HIV risk', and noted need for research on PrEP in pregnancy and lactation[40], and updated WHO guidance from 2017 recommended the use of PrEP in pregnancy and lactation, although important safety and implementation gaps remain. Implementation science studies can provide a framework to increase accrual of safety and effectiveness data of PrEP in pregnancy while refining programmatic strategies.

1.2 Rationale

1.2.1 Adherence-concentration- thresholds for TDF/FTC PrEP

For men who have sex with men (MSM), the STRAND study of directly observed PrEP (DOT) and DOT PrEP study[41] in DBS defined benchmark blood concentrations of TFV-DP arising from 2, 4, and 7 DOT doses/week (i.e., 25th percentiles of 350, 700, and 1,250 fmol/punch for 2, 4, and 7 doses/week, respectively (**Table 1**)[8, 41, 42]. When these STRAND data were then applied to the iPrEx trial clinical

cohort[43], they helped quantify TFV-DP concentrations and the required adherence frequency associated with HIV protection for MSM[43, 44], results that have shaped global PrEP guidelines and messaging. These STRAND-iPrEx adherence-efficacy thresholds are being applied to studies of cisgender women taking PrEP currently. However, the protective drug concentrations in the blood and the number of tablets per week required to achieve those levels may differ depending on the route and frequency of exposure to HIV[45].

Table 1: Adherence-concentration threshold for interpreting adherence to PrEP

	US population-derived thresholds for men	African cisgender women-specific thresholds
Adherence Interpretation	DBS TFV-DP Concentration	TFV-DP concentrations
7 doses/wk	≥1250	??
4-6 doses/wk	700-1249	??
2–3 doses/wk	350–699	??
<2 doses/wk	<350	??

Robust data to understand the pharmacology of TDF and FTC in the female genital tissue, and its relationship with PrEP adherence levels needed to achieve HIV protection are limited; the DOT-DBS[41] and HPTN 066 studies included men and cisgender women but were all from the US and blood concentration-HIV protection relationships for cisgender women have never been defined. Indeed, the FDA withheld approval of Descovy (TAF/FTC) PrEP for cisgender women due to lack of data on pharmacologic-HIV protection relationships for Truvada in cisgender women which were needed to complete efficacy extrapolation for Descovy. Single-dose tissue pharmacology studies of oral TDF/FTC PrEP dosing suggest cisgender women may need extraordinary adherence perfection for HIV protection than their MSM counterparts – at least 6 doses of oral TDF/FTC per week to achieve protective concentrations in vaginal tissue. Several hypotheses have questioned whether PrEP benefits may be compromised in younger cisgender women, including immature genital mucosa, higher proportion of exposed vaginal tissues and elevated pro-inflammatory cytokines in genital secretions[46-48]. On the other hand, in recent clinical studies in African cisgender women with reasonable-but-imperfect PrEP adherence, there is evidence to suggest important levels of HIV protection. For example, in the HPTN 082 study of 16-25 year old cisgender women[49], 84% had detectable TFV-DP in DBS (median 485 fmol/punch = 2-3 doses/week) but only 25% had DBS TFV-DP levels that suggest "high adherence" based on current STRAND-derived thresholds (TFV-DP >700 fmol/punch). Notably, in this cohort with high risk for HIV (median VOICE risk score was 7; score ≥5 associated with >6% HIV incidence in prior cohorts) and moderate adherence, the annual HIV incidence was only 1%. Given the current lack of equipoise, defining cisgender women-specific adherence-concentration thresholds for PrEP is not only essential for the field but long overdue.

1.2.2 Pharmacology of TFV and TFV-DP in African cisgender women.

Work we have done using data from large PrEP studies[9, 50] in African populations suggests the STRAND adherence-blood concentration thresholds derived from US individuals may not accurately reflect the pharmacology of African cisgender women,[51, 52] in general and particularly in pregnancy when TFV-DP concentrations even after adjusting for objective measures of adherence may be 45%-58% lower.[52] Among cisgender women with objective evidence of 100% adherence (by MEMS), median TFV-DP levels in DBS for both non-pregnant and pregnant periods were all below 1250 fmol/punch, the US derived TFV-DP concentration threshold for 100% adherence (7 doses per week) (**Table 2**). Moreover, only 62% and 44% of individuals taking ≥4 doses/weeks and ≥6 doses/weeks,

Table 2. Sensitivity of US-derived TFV-DP threshold to detect consistent doses taken over prior 4 Weeks in African persons. *Source*: Pyra et al. HIVR4P 2018

≥700 fmol/punch (≥4 Doses per Week)	≥1050 fmol/punch (≥6 Doses per Week)	≥1250 fmol/punch (7 Doses per Week)
Sensitivity (95% CI)	Sensitivity (95% CI)	Sensitivity (95% CI)
**62%** (52%, 72%)	**44%** (31%, 56%)	**19%** (2%, 36%)

respectively, based on MEMS data were also correctly identified in STRAND-derived categories[51]. We previously showed that some nucleoside (i.e., acyclovir) PK in African cisgender women differs from US cisgender women[53]. This may also differ for TDF/FTC PrEP due to effects of race, sex, anemia (common in populations in Africa setting), physiologic changes of pregnancy (e.g., increased clearance[54]), drug metabolism between male and female, or pharmacogenomics factors as well that may be specific to African cisgender women. Given that MEMS may not be a reliable objective adherence measure[55] and absence of robust clinical data on the relationship between TFV-DP concentrations with HIV protection for African cisgender women, only controlled DOT pharmacologic studies can definitively discern adherence from true pharmacologic effects as well as the clinical significance of these observation.

1.2.3 Pharmacokinetic TDF/FTC PrEP Adherence in Pregnancy

Work we have done recently suggest that TFV and TFV-DP concentrations may be lower in pregnant than non-pregnant periods[52]; concentrations adjusted for adherence were 45%-58% lower during pregnancy, with the reduction more pronounced in the 3rd trimester **(Figure 2)**. Indeed, our pregnancy results have now been replicated by recent IMPAACT 2009[56], a PK study among 40 16-24 years old HIV-uninfected pregnant and postpartum women and their infants (n=20 singleton pregnancy at 14-24 weeks' gestation and n=20 postpartum within 6-12 weeks after delivery) to establish the plasma and DBS drug concentrations associated with daily directly observed oral TDF/FTC PrEP during pregnancy and postpartum conducted in Uganda, Zimbabwe, and South Africa. These data showed that DBS TFV-DP level in pregnant women were nearly 30-40% lower than in postpartum women[56]. However, IMPAACT 2009 study did not measure levels in PBMCs needed to link cumulative dosing thresholds and TFV-DP concentrations with PrEP efficacy in cisgender women. Thus, the proposed is complementary and will provide new data on drug levels in PBMC to address this knowledge gap. Changes in pregnancy may include: 1) increased gastrointestinal transit time that can alter the rate and extent of drug absorption; 2) changes in total body water and fat, increasing drug distribution volume; and 3) increased renal elimination. These results, as well as evidence that women may require higher adherence levels for similar PrEP efficacy than MSM because of lower active drug concentrations in genital versus rectal compartments[57], warrant controlled studies with DOT PrEP in pregnant women.

Figure 2: Concentrations among Cisgender women with 100% Adherence by MEMS (Pyra, Anderson et al AIDS 2018)

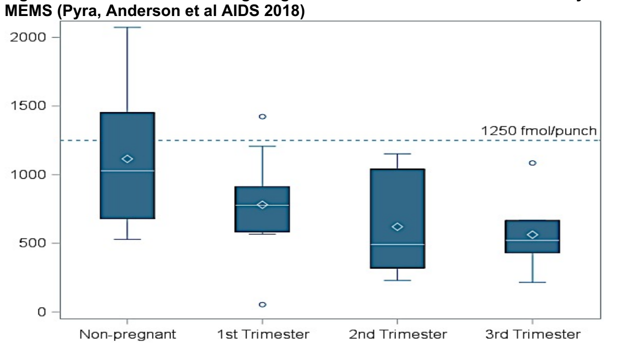

1250 fmol/punch: US male–derived TFV-DP concentration threshold for 100% adherence (7 doses/week)

Experience with DOT PK study of TDF/FTC PrEP in African cisgender women: We have strong field experience in conducting intensive PK[58, 59] and antiviral medication studies in Africa[9, 50, 60-62], including in understudied populations[58]. We conducted the only PK study of maternal and infant exposure to TDF/FTC PrEP via breastmilk in Uganda and Kenya (ClinicalTrials.gov: NCT02776748)[58], achieving 100% retention and demonstrating limited breast milk excretion and low infant absorption. The data from that study directly informed WHO guidelines on the use of PrEP in breastfeeding (**Figure 3**)[58, 63]. We will utilize our experience from this study to refine our approach in this proposal.

Figure 3. Maternal and infant TFV level when PrEP is used by breastfeeding cisgender women.

Mugwanya et al PLOS Med 2016

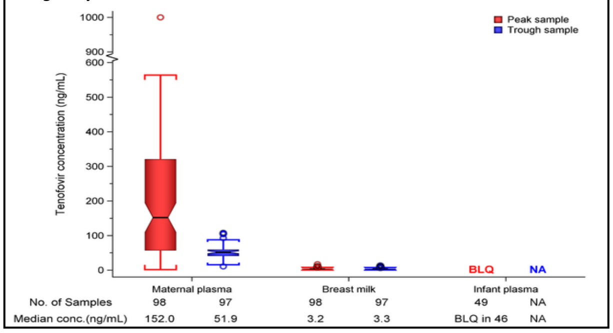

1.3 Problem statement

Defining the optimum dosing strategy for a population, subgroup, or individual patient requires resolution of the variability issues. Pharmacologic measures of drug exposure are critical to the interpretation of results of PrEP studies and implementation programs. Benchmark concentrations for TFV-DP in DBS and, PBMCs with DOT dosing have been established for US individuals. Because many antiretroviral drugs including TDF-based PrEP may exhibit variable pharmacokinetics due either demographics, genetic variation in drug transporter physiological changes in pregnancy, or pathological changes, context-specific studies are needed. No controlled studies have been conducted in African cisgender women including pregnant cisgender women.

1.4 Justification

African cisgender women, particularly those ≤24 years, face disproportionate HIV risk, accounting for more than half of new infections on that continent, and with incidence rates that are often double or more than their male age-mates. Thus, African cisgender women at risk for HIV are a priority group for HIV prevention, including PrEP, but variable adherence in PrEP clinical trials and limited pharmacologic data have resulted in lack of clarity about the degree of PrEP use required for HIV protection. For MSM, the STRAND and DOT-DBS studies of DOT of TDF/FTC PrEP defined blood TFV-DP concentrations arising from 2, 4, and 7 DOT doses per week. When STRAND data were applied to the iPrEx trial cohort they helped define robust adherence-efficacy thresholds for MSM. Those thresholds are today being applied to studies of African cisgender women taking PrEP, and cisgender women-specific HIV protective levels have never been defined. However, recent data from large PrEP studies we have done among African cisgender women suggest those STRAND levels may not reflect the pharmacology of cisgender women in African settings, in general and particularly in pregnancy when TFV-DP concentrations may be 45%-58% lower. Single-dose tissue pharmacology studies suggest oral TDF/FTC PrEP dosing delivers 20- to 100-fold higher TFV-DP in rectal tissue compared with vaginal tissue, suggesting cisgender women may need extraordinary perfection for HIV protection; on the other hand, in clinical studies in cisgender women with reasonable-but-imperfect PrEP adherence, suggest important levels of HIV protection. Furthermore, pregnant cisgender women have been excluded from many early studies of novel therapeutics, including PrEP, resulting in delays for pregnant cisgender women to gain access to PrEP and benefit from normative recommendations about the safety of PrEP in pregnancy and breastfeeding[64, 65]. Recent data from the IMPAACT 2009 study of DOT PrEP in pregnant and postpartum cisgender women showed that DBS TFV-DP concentrations were 30-40% lower in pregnant vs postpartum cisgender women[56]. However, this study did not assess concentrations in PBMCs which are relevant to address the critical question of whether the observed reductions are of clinical significance. Thus, there is need to understand blood concentrations of PrEP medications and their relationship to HIV protection in non-pregnant and pregnant cisgender women. A DOT study of PrEP in cisgender women to define adherence-concentration thresholds is overdue and completely necessary for the field.

2 STUDY OBJECTIVE AND DESIGN

2.1 Overall Hypothesis

TDF/FTC dose-response thresholds from DOT PrEP dosing for cisgender women will be defined but will differ from thresholds defined for MSM populations; pregnant cisgender women will have lower benchmark concentrations in PBMC.

2.2 Overall Objective

The central objective of this proposal is to describe blood and tissue adherence-concentration benchmarks for DOT TDF/FTC PrEP for Kenyan cisgender women and quantify the impact of pregnancy on benchmark thresholds.

2.3 Primary Objectives

1) To define the cisgender women-specific expected blood concentrations and dose-proportionality for TFV-DP in DBS and PBMCs in cisgender women using directly observed TDF/FTC therapy at 2, 4, 7 doses per week.

2) To establish a model to predict adherence rate to TDF/FTC by level of TFV-DP in DBS for cisgender women.

2.4 Secondary Objectives

1) To define the expected concentrations and dose-proportionally for TFV-DP in PBMC and vaginal tissue

2) To quantify the effect of pregnancy on the benchmark concentrations of TFV, TFV-DP, FTC, and FTC-TP in pregnant cisgender women using daily DOT FTC-TDF PrEP

3) Examine relationships among drug concentrations in plasma, WB, DBS, and PBMC.

4) To determine the influence of biological variables (e.g. mean corpuscular volume and HCT, age, weight, sex, metabolites from metabolomics, and genetic variability in drug transporter/clearance proteins) on drug concentrations.

5) Compare drug concentrations in DBS from fingerstick versus transferring blood from blood tubes.

2.5 Study Design

We will implement two distinct but complementary study designs: 1) An open-label, randomized, three-arm, directly observed therapy, pharmacologic study among low-risk HIV-uninfected non-pregnant cisgender women, and 2) A contemporaneous cohort of pregnant cisgender women at high risk for HIV to receive daily directly observed therapy PrEP dosing.

2.5.1 Study design for non-pregnant cisgender women

This is an open-label, randomized, three-arm, directly observed therapy, pharmacologic study. HIV-uninfected non-pregnant cisgender women at low risk for HIV will be randomly assigned to 1 of 3 dosing frequencies of DOT TDF/FTC PrEP **(Figure 4)**: **2, 4, or 7** doses per week to help differentiate poor and modest from perfect dosing (**Figure 4**).

Figure 4: Schema for directly observed TDF/FTC PrEP dosing and pharmacologic sampling

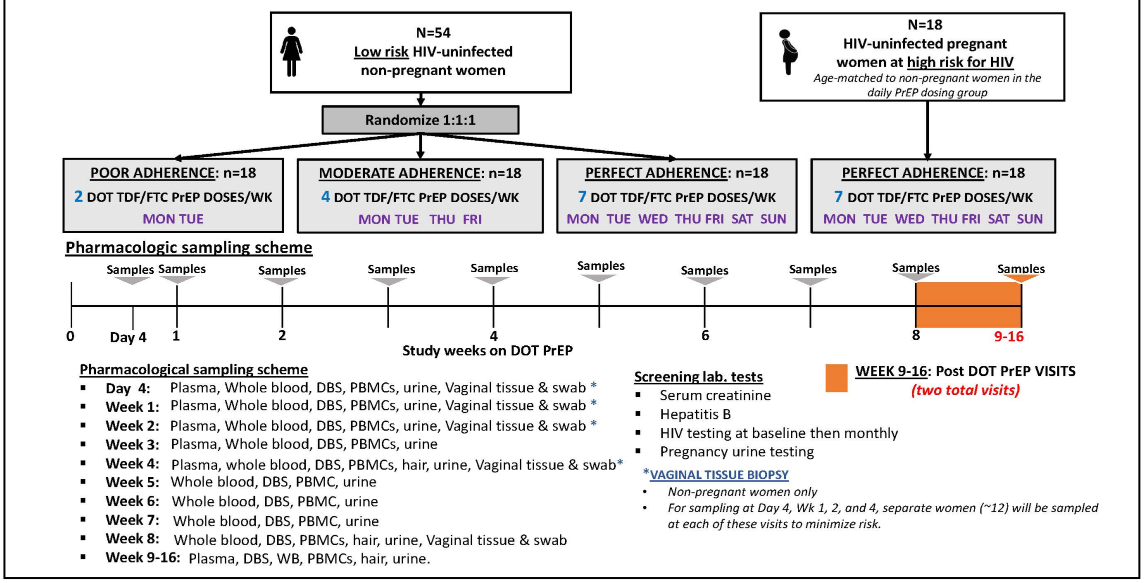

Enrollment will continue until 54 for non-pregnant evaluable participants (i.e., participant with completed data on dosing and PK samples) are enrolled.

– **Group #1**: "7 doses per week or Perfect Adherence arm"— cisgender women will receive a single tablet of co-formulated 300 mg TDF/ 200mg FTC once daily (7 doses per week).
– **Group #2**: "4 doses per week or Moderate Adherence arm" — cisgender women will receive a single tablet of co-formulated 300 mg TDF/ 200mg FTC tablet on Monday, Tuesday, Thursday, Friday.
– **Group #3**: **"**2 doses per week or Poor Adherence arm" — cisgender women will receive a single tablet of co-formulated 300 mg TDF/ 200mg FTC tablet on Monday and Tuesday.

Justification for non-daily dosing of PrEP groups:

In this study we propose to randomize cisgender women (non-pregnant cohort) to non-daily dosing of PrEP (2, or 4 doses per week). The non-daily dosing is needed to help investigate and demonstrate dose proportionality of TFV and TFV-DP concentrations. The adherence-concentration thresholds derived from this dosing frequency will help guide clinical decisions on drug levels associated with HIV protection in cisgender women and adherence level required to achieve those concentrations. Only daily PrEP is recommended by WHO and the Kenya national guidelines for protection from vaginal HIV exposure and we have carefully considered the risks associated with non-daily in planning this proposal. As detailed in eligibility section, only non-pregnant cisgender women at low HIV risk based on Kenya guidelines will be enrolled and cisgender women will receive full package of HIV prevention, including risk reduction counseling, condoms and STI treatment at each visit. We will also perform monthly HIV testing.

2.5.2 Study design for pregnant cisgender women

This will be an open-label, non-randomized, single-arm, pharmacologic study of daily directly observed therapy. In addition to the cohort of non-pregnant cisgender women describe above, a contemporaneous cohort of up to 18 pregnant cisgender women will be recruited to evaluate the impact of pregnancy on blood and cellular drug benchmark concentrations. Cisgender women must be **at 13-26 weeks gestation** to give sufficient time for 8 weeks of DOT follow-up. This gestation age was chosen to balance feasibility and risk-benefit and is informed by preliminary work we have done that shows greater reduction in TFV and TFV-DP concentrations in the 2nd and 3rd trimester's compared to the 1st trimester[52]. We will make all effort to have cisgender women across the entirety of this gestation age spectrum to allow analyses that look across the 2nd and 3rd trimester. To ensure an ethical approach for provision of PrEP in pregnancy (i.e., only exposing PrEP to those who want and might benefit from it) all pregnant cisgender women will be at high risk for HIV and willing to use PrEP. HIV risk and eligibility will be determined according to the Kenya guidelines for PrEP, as described in Table 3. Pregnant cisgender women will receive only daily PrEP dosing (7 doses per week) and matched on age to non-pregnant cisgender women in daily dosing arm. After 8 weeks of DOT PrEP, pregnant cisgender women will be linked to continue PrEP at the clinic of their choice, including at the Thika site clinic itself, permitting seamless access to PrEP and continuation after the study.

Table 3: Kenya national guideline indicators of substantial risk for HIV and indication for PrEP:

Partner of HIV-infected person not on ART or on ART for <6 months
Has >1 partner of unknown status
Engages in transactional sex
History recent STI (past 6 months)
Recurrent PEP use
Inconsistent or no condom use
Injection drug use.

2.6 Enrollment Targets

A cohort of up to 72 cisgender women who meet the inclusion and exclusion criteria described in Section 3 below will be enrolled at the KEMRI Thika Research Site, in Thika, Kenya. The target enrollment is to have at least 60 evaluable participants (both pregnant and non-pregnant) at the end of the study (i.e. have complete set of all protocol pharmacological samples). As described in Section 3.8, women with incomplete set of pharmacological samples will be replaced but no more 20% or up to 72 total cisgender women who agree to enroll. i.e., 54 low risk non-pregnant cisgender women and 18 pregnant cisgender

women at elevated risk of HIV. The cohort of 54 cisgender women who accept PrEP will be randomized 1:1:1: **2, 4, or 7** DOT doses per week to help differentiate poor and modest from perfect dosing. Pregnant cisgender women will receive only daily DOT dosing.

3 STUDY POPULATION

We will enroll a novel cohort of up to 54 healthy, HIV-uninfected non-pregnant Kenyan cisgender women volunteers 18-30 years to establish benchmark adherence-concentration thresholds for TDF-based PrEP, plus a contemporaneous cohort of up to 18 pregnant Kenyan cisgender women at elevated HIV risk to quantify the impact of pregnancy periods on benchmark adherence-concentration thresholds. Participants will be selected for the study according to the criteria in Sections 3.1 and 3.2. They will be recruited, screened, and enrolled as described in Section 3.3. Issues related to participant retention and withdrawal from the study are described in Sections 3.5 and 3.6, respectively.

3.1 Inclusion Criteria

This study will enroll young Kenyan cisgender women (aged 18-30 years old). Cisgender women bear the greatest burden for HIV infection; thus, the study will focus solely on cisgender women. HIV incidence is greatest in younger cisgender women, as is PrEP interest in accumulating data from Kenya, and the age limitation reflects that clinical and epidemiologic need. Enrollment criteria will be finalized at the time of protocol development to account for any updates to Kenyan national guidelines, but the central clinical criteria are as follows:

For all cisgender women

- Age ≥18 and ≤30 years old
- Willing to undergo urine pregnancy tests
- Has understood the information provided and has provided written informed consent before any study-related procedures are performed.
- HIV uninfected based on negative HIV rapid tests, according to Kenyan national algorithm
- Normal renal function (estimated glomerular filtration rate >60 mL/min)
- Hepatitis B surface Ag negative
- No active clinically significant medical or psychiatric conditions that would interfere with study participation
- Lack of severe anemia
- Willing to use DOT and come to clinic frequently for DOT PrEP for at least 8 weeks
- Willing to have home visits for follow up
- Has an active smartphone to allow off-site observation of dosing
- Intention to stay within the study site's catchment area for at least 8 weeks.
- Resides or works in catchment area with high speed internet coverage to permit video streaming

Specific for non-pregnant cisgender women cohort

- Not pregnant or breast feeding
- At low risk for HIV. *In Kenya, national guidelines define substantial risk for HIV and recommend PrEP be an option for individuals reporting: partner of HIV-infected person not on ART or on ART for <6 months, >1 partner of unknown status, transactional sex, recent STI, recurrent PEP use, inconsistent condom use, or injection drug use. So, non-pregnant cisgender women reporting any of these factors will not be eligible for the study but will be linked for PrEP at clinic of choice including at Thika Site itself.*
- Willing to be randomized to non-daily PrEP and come to clinic frequently for DOT PrEP
- Willingness and ability to be abstinent for at least 7 days after each vaginal biopsy visit.

Specific for pregnant cisgender women only

- At screening, evidence of a viable pregnancy with gestational age of 13-26 weeks after the date of conception with sonographic confirmation. *If adequate sonographic results are not available from medical records at screening, an ultrasound must be performed in the interim so that the result is available at study entry.*
- At elevated risk for acquiring HIV according to Kenya guideline for PrEP. This is to ensure an ethical approach for provision of PrEP in pregnancy (i.e., only exposing PrEP to those who want and might benefit from it). *Kenya national guidelines define substantial risk for HIV and recommend PrEP be an option for individuals reporting partner of HIV-infected person not on ART or on ART for <6 months, >1 partner of unknown status, transactional sex, recent STI, recurrent PEP use, no or inconsistent condom use*
- At study entry, willing to use PrEP during pregnancy for HIV prevention

3.2 Exclusion Criteria

For all cisgender women

- Inability to give informed consent
- Positive screening HIV+ as determined by standard rapid serologic assays or suspected acute HIV infection in the opinion of the clinician. (example signs and symptoms of acute HIV infection include combinations of fever, headache, fatigue, arthralgia, vomiting, myalgia, diarrhea, pharyngitis, rash, night sweats, and adenopathy cervical or inguinal)
- Positive HBV surface antigen test at screening
- Calculated creatinine clearance < 60 ml/min.
- Any laboratory value or uncontrolled medical conditions that, in the opinion of the investigators, would interfere with the study conditions such as, heart disease and/or cancer.
- Prohibited concomitant medications are: investigational agents (within 30 days of enrollment), aminoglycosides, ganciclovir/valganciclovir, chronic high-dose acyclovir/valacyclovir (>800mg acyclovir or > 500mg valacyclovir for >7 days), cyclosporine, amphotericin B, foscarnet, and cidofovir, and products with same or similar active ingredients as the study medications including TAF®, ATRIPLA®, COMPLERA®, EMTRIVA®, VIREAD®; or drugs containing lamivudine or adefovir, which are close analogs of FTC and tenofovir, respectively.
- Current or past use of PrEP (pre-exposure prophylaxis)
- Not willing to have home visits

Specific for non-pregnant cisgender women cohort

- Pregnancy or plan to become pregnant in the next 6 months or unwillingness to use birth control
- Current breastfeeding
- High risk of HIV infection (for example: sexually active with an HIV infected partner; engages in condomless intercourse with HIV-infected partners or partner of unknown status during the study; females who exchange sex for money, shelter, or gifts; active injection drug use or during the last 12 months; newly diagnosed sexually transmitted infections in last 6 months.

Specific for pregnant cisgender women cohort

- Mother has a known history of any of the following, as determined by the site investigator or designee based on maternal report and available medical records:
- Sickle cell anemia (excluding sickle cell trait), chronic bleeding, blood transfusion within the past 120 days (excluding for chronic illness) or other blood dyscrasias
- Fetus has a known or suspected major congenital anomaly, from chart review of prior data, defined ultrasound.
- Complications in prior pregnancies that would be considered exclusionary

3.3 Study Setting

The proposed work will take place in Thika, Kenya. The site has extensive experience conducting large biomedical and PK studies[9, 58, 60, 62, 66-72] and has a long-standing relationship with local public health authorities, HIV prevention advocates, and community leaders. Over the past decade, the Thika site has established a multi-disciplinary team focused on HIV prevention research (more than a dozen projects currently) and on provision of clinical care, including clinical studies involving follow up of pregnant cisgender women**.** The Thika site is a center of excellence for PrEP in Kenya and leads PrEP technical assistance to other clinics as part of Kenya national PrEP scale-up. As stated in the background section, the Thika site conducted the DOT PrEP PK study in breastfeeding cisgender women (**see Figure 3**)[58], achieving 100% retention.

3.4 Recruitment and Prescreening Process

We will enroll a novel cohort of up to evaluable 72 (Up to 54 non-pregnant cisgender women, and up to 18 pregnant cisgender women) healthy, HIV-uninfected adult cisgender women volunteers. The experienced community outreach team at the study site in Thika employs community-based mobilization strategies, including working with the neighboring Thika District Hospital to identify pregnant cisgender women, engaging community health volunteers, gate keepers, peer to peer mobilization, youth peer providers and ambassador models, use of printed and electronica IEC materials, social media (WhatsApp and Facebook), radio talk shows in English and Kiswahili, active participation in local social events for information sharing, and partnering with learning institutions to give health talks and to provide study related information. The methods for recruitment proposed in this study are similar to those that were successfully used to conduct the pharmacokinetic study of PrEP in Breastfeeding cisgender women at the Thika site. From these strategies, we will identify those that are highest yielding and shift our focus to primarily rely on them. The outreach team at PHRD is well-suited to develop innovative recruitment methods and collaborate with local partners to ensure that we achieve the accrual rate needed and enroll cisgender women including pregnant cisgender women who are highly dedicated to research procedures. The Thika team has >10 years of experience of conducting clinical trials, including in populations of cisgender women and pregnancy cisgender women (**Table 4**). These studies involving pregnant cisgender women at Thika site are summarized briefly in the Table below further demonstrate our capacity and scale to do this work in pregnant cisgender women. In many of the studies we have conducted, the Thika site has achieved the highest recruitment and data quality site compared to others in multisite/multinational studies – for example, in the Partners PrEP Study clinical trial of PrEP, and the MPYA Study. We will replicate and extend all of those previously proven recruitment strategies for this study.

Table 4: Studies involving pregnant cisgender women at Thika

Study and funding	***Number of cisgender women enrolled***	***Number of cisgender women who got pregnant during study***
Partners PrEP *(BMGF)*	*112*	*21*
Partner Demo Project *(R01MH0559907)*	*332*	*41*
SCIP *(R00HD076679)*	*74*	*40*
MPYA *(R01MH109309)*	*175*	*55*
NuvaRing (*R01HD077872)*	*122*	*1*
GHS *(CDC U48DP005013)*	*400*	*143*

3.5 Co-Enrollment Guidelines

Participants in this study should not take part in any concurrent research studies that use drugs or medical devices while on follow up. Co-enrollment in observational or other studies may be allowable with approval of the site IoR or designee. Previous participation in the placebo arm of an HIV vaccine trial may be allowable with approval of Co-Chairs.

3.6 Retention of Study Participants

For this DOT pharmacological study, high retention begins with the exclusion of participants who indicate a high likelihood of travel outside of the study area, who appear to lack dedication to the research process, or who may experience personal challenges with compliance with study visits. We will follow usual Kenyan clinical practices for notifying participants of their study follow-up visits, which include sending participants reminders using SMS messaging and telephone calls. In addition, an extensive tracing record at the time of study entry will be completed by a trained research staff member and will be used to trace participants who miss visits; the experienced community outreach team has established methods for successful participant tracing. As detailed in the Introduction Section, the Thika Site successfully recruited lactating cisgender women (and their infants, half <13 weeks of age) into a similar DOT PrEP PK study previously, achieving 100% retention for breastfeeding-month infant pairs and sample collection. The following approaches will be used to promote compliance and achieving planned evaluable with pharmacological sample collection.

Medication calendar: At study enrollment, subjects will be given a calendar for study visit appointments, dosing schedules, and to record unsolicited adverse events. For those interested and willing, visit schedule and reminders will be added to the calendar on their smartphones.

Video streaming and phone reminders: Flexible clinic scheduling will be available to promote high rates of follow-up and compliance and minimize participant's burden. Video live streaming services via social media (e.g., WhatsApp, Skype, etc.); only cisgender women with an active smart phone will be enrolled in this pharmacological study to permit offsite observation dose taking required DOT PrEP dose. Enrolled participants will receive a reminder message (sent via text message or phone call) every morning of each scheduled study visit. A one-month supply of back-up medication will be provided to participants at study enrollment to be used if participant is unable to complete visit at the clinic; all these doses taken offsite will still be directly observed by study staff by live video streaming on smart cell phone or computer via WhatsApp. For DOT doses taken via video streaming, participants will be asked to open their mouth afterwards to confirm swallowing.

Only cisgender women with an active smart phone will be enrolled and will be supported with weekly data/internet bundles consistent with the local IRB. The remote DOT via video streaming is intended to increase compliance as well as minimize participant burden, especially pregnant cisgender women.

Home visits: We will conduct complimentary home visits for patients unable to attend their scheduled clinic visits or procedure to promote compliance to study procedures. During home visits, study staff may conduct study procedures e.g., observing of dosing taking or collection of study samples (blood, urine, hair) where applicable. Any offsite collection of study sample will follow the same safety standards followed at the clinic. For this pharmacological study, only cisgender women who consent for home visits will be enrolled.

3.7 Participant Withdrawal

Regardless of the participant retention methods used, participants may voluntarily withdraw from the study for any reason at any time. The Site Investigator of Record or designee also may withdraw participants from the study to protect their safety and/or if they are unwilling or unable to comply with required study procedures. Participants also may be withdrawn if the study sponsor, government or regulatory authorities, or site IRBs/ECs terminates the study overall or at a specific site prior to the last scheduled participant visit.

Every reasonable effort will be made to complete final evaluations for participants who terminate from the study prior to the last study assessment or visit; study staff will record the reason(s) for all withdrawals from the study in participants' study records.

3.8 Replacement of Study Participants

Data from people who permanently discontinue study treatment will be used in analyses. Study participants who miss two consecutive scheduled dosing or miss any pharmacological samples or permanently discontinue DOT PrEP will be replaced so that approximately 60 evaluable participants complete the study. No more than 20% of the target population (n=60) will be replaced for maximum of 72 participants. Data from subjects who discontinue prematurely will be used in analyses. In the non-pregnant cohort, cisgender women who get pregnant will be replaced but continue to be followed up to the end of their scheduled protocol study follow up. Seroconversions (unlikely though possible) will not be replaced.

4 STUDY PRODUCT

The study product is Truvada formulated as emtricitabine 200 mg (FTC) / tenofovir disoproxil (TDF) fumarate 300 mg.

4.1 Study Product Formulation

Each TDF/FTC tablet contains 200 mg of emtricitabine and 300 mg of tenofovir disoproxil fumarate. An authorized study clinician will prescribe TDF-FTC as part of this study, with dosing instructions as described in this protocol.

4.1.1 Study product storage

Prior to dispensing, TDF/FTC tablets will be stored in the pharmacy in the original container. Each bottle contains a desiccant to protect the product from humidity. Store at 68 °F to 77 °F (20 °C to 25 °C). Excursions permitted between 15° to 30°C.

4.2 Source of Study Product and Accountability

Study medication (TDF/FTC) will be purchased at wholesale in Kenya. The pharmacist of record will receive and inventory the study medications, will dispense and track prescriptions, and will provide directions for returning unused medications. The pharmacist of record will track the accountability of the study medications according to established study procedures.

4.2.1 Study product dispensation

Two supplies of study drug will be dispensed for each participant. Supplies will be dispensed in the original containers. Study personnel will be in possession of one supply, the main medication supply, and will keep it stored in a locked cabinet except for transporting it to and from the directly observed dosing visits. This main supply will be enough for the entire 8-week DOT dosing period. Study personnel will record the date that a bottle is opened on the bottle and will ensure that opened bottles are used within 8 weeks. The locked cabinet will be in a temperature-controlled building, which is maintained at approximately 21°C. A second medication supply, a take-home supply, will be given to participants for non in-person dosing (video-streaming). Participants will be reminded to keep the medication in the original container with the desiccant, and to store in a dry place with the cap tightly sealed. This take-home supply will be a maximum of one bottle of medication, additional bottles will be given to participants when the current bottle is exhausted.

4.2.2 Return and Destruction of Study Product

Unused medications will be returned to the site study pharmacy at the end of the dosing periods or after a participant permanently discontinues study drug. The site pharmacy will account for and manage the returned medications in accordance with the local policies and procedures.

4.3 Assessment of Participant Adherence with Study Product/Intervention

Adherence will be assessed with directly observed dosing. All doses will be observed in person, when possible, at an agreed-upon private room at the research clinic, or other predetermined location to maximize convenience for the participant. In circumstances when doses cannot be witnessed in person, doses will be witnessed by live audio-video communication. If video confirmation fails, this will be recorded as non-DOT dose.

4.4 Concomitant Medications

Concomitant medications will be evaluated on a case-by-case basis for interactions with the study medications. Certain medications are permitted, and other medications are prohibited as described in the sections that follow. Medications not listed in these sections will be evaluated for potential interactions with the study medications or procedures and a decision to enroll the subject will be made accordingly. Additionally, should a new concomitant medication be started during the study, the same evaluation will take place.

4.5 Prohibited Medications

Prohibited concomitant medications are: investigational agents, aminoglycosides, ganciclovir/valganciclovir, chronic high-dose acyclovir/valacyclovir, cyclosporine, amphotericin B, foscarnet, and cidofovir, and products with same or similar active ingredients as the study medications including ATRIPLA®, COMPLERA®, EMTRIVA®, VIREAD®; or drugs containing lamivudine or adefovir, which are close analogs of FTC and tenofovir, respectively.

4.6 Precautionary Medications

Any medication or herbal supplement not listed under sections prohibited medication section will be evaluated on a case-by-case basis for interactions with the study medications. Should there be potential for serious interaction, based on the opinion of the investigators, the subject will not be invited to participate. Additionally, should a new concomitant medication be started during the study, the same evaluation will take place.

4.7 Toxicity Management

Safety related laboratory testing will be performed at study entry and at week 8 and or as indicated by clinician during follow up. If enrollment occurs more 30 days after screening, entry safety labs will be repeated (HIV rapid testing, Complete blood count, HBV surface antigen, serum creatinine, urine pregnancy tests. Toxicity will be managed in consultation with a clinician on the protocol. The clinician may discontinue the study drug at any time if he/she feels that continued use would be harmful to the participant. The PSRT should be consulted when possible. The PSRT will be consulted when considering further holding or recommencing study medication.

4.8 HIV Seroconversion

Frequent testing for HIV acquisition during the study period will allow prompt cessation of study drug in an HIV-infected participant, minimizing the risk that resistant virus will emerge. Therefore, HIV testing will be performed at all monthly study visits. HIV rapid testing and specimen collection should be completed prior to administration of study medication. Participants who have any reactive or positive HIV test result after initiating PrEP will be instructed to discontinue study drug immediately and will have further testing to clarify their HIV infection status, as described in Section 5.3. Any enrolled participant who is confirmed

to have acquired HIV infection during the study will permanently discontinue study product and will be transitioned to a local HIV care clinic for appropriate follow-up and clinical management. Procedures for participants who acquire HIV infection are described in Section 5.3.

4.9 Dose Modification

Study personnel will record all events of participants who vomit just after dosing. Participants will not be asked to re-dose, unless they are confident that a recurrence of vomiting can be avoided. Additionally, re-dosing will only be allowed if an intact tablet is visible in the emesis. The same directly observed dosing rules will apply if the re-dosing will be attempted. Dose modifications of up to 3 sequential missed doses will be allowed if a participant has, for example, an acute gastrointestinal illness. Further dose modifications or other dose modification issues will be discussed with the PSRT. The PSRT may decide on further missed doses, or participant withdrawal from the study.

5 STUDY PROCEDURES

An overview of the study visits and procedures schedule is presented in Table 5. Presented below is additional information on visit-specific study procedures.

5.1 Screening Procedures

Screening COVID-19 symptoms and protection measure: We will follow the standard of care Kenya Ministry of Health, KEMRI, and WHO guidelines on the COVID-19 virus. Participants will be informed about practicing good hand hygiene, masking, good respiratory hygiene, and maintaining social distancing. Staff will adhere to social procedures including maintaining 6 –feet part, wearing masks, distance. Participants and staff will be screened for COVID-19-related symptoms when they arrive at the clinic using a checklist to check for risk of COVID-19 exposure based on symptoms or contacts. Any person that comes to the clinic with symptoms consistent with COVID-19 that include 1 or more of the following: fever, cough (dry or productive), dyspnea, myalgias will be isolated. Participants will be educated about the Ministry of Health specified measures for seeking healthcare during this time. Referral mechanisms to local public health facilities are in place. Finally, the clinical site will maintain a register of all visitors and participants who enter the study site, to support usual care contact tracing efforts where necessary.

At the screening visit a qualified study clinician will perform a history and physical exam for each participant. A complete medication history will be obtained including all drug allergies, immunization history, prescription, over the counter, and herbal treatments. Vital signs will include height, weight, blood pressure, pulse, respiratory rate and temperature.

- Informed consent
- Safety Labs: Complete blood count (CBC, specifically hematocrit and hemoglobin), HBV surface antigen, serum creatinine)
- HIV antibody (rapid test or blood)
- Urine pregnancy test
- Medical, medication, vaccine history
- Physical exam

A screening test(s) may be repeated within the 30-day screening window. A participant who is unable to schedule a study entry visit within the 30-day screening window must be re-screened prior to entry into the study. Participants who have not been immunized for HBV will be offered immunization, as this is standard of care. An HBV Antibody test may be ordered for participants who are unsure of their vaccination history.

Fetal Ultrasound

Specific for pregnant women, a fetal ultrasound should be performed during the study screening period to estimate gestational age and to document multiple gestation; multiple gestation is not an exclusionary criterion. Because ultrasound-based gestational dating is more accurate the earlier a woman is in her pregnancy, prior ultrasound results obtained from antenatal/medial sonographic results from the current pregnancy will be used to meet this requirement, provided that a report of biometric measurements is available as documentation. If sonographic results from the current pregnancy are not available from medical records at the time of screening, a fetal ultrasound scan will be performed to confirm (or used to re-calculate) gestational age at entry into the study. For pregnant women without fetal ultrasonic records one as part of their prenatal care for the current pregnancy, the study will arrange for and pay for fetal ultrasound as part of study screening procedures. Scans should be performed during the 30-day study screening period, and results must be available at the time of enrollment. Ultrasound scans may be performed at the study site or at off-site facilities. A result report that minimally documents the following must be obtained for filing in participant study charts and entry into: Date of scan, number of fetuses, biometry measures (e.g., crown-rump length, femur length, biparietal diameter, head circumference, abdominal circumference). Ultrasound biometric measurements (e.g., crown-rump length, femur length, biparietal diameter, head circumference, abdominal circumference) to document gestational age estimate and will subsequently be used to confirm (or change) the gestational age at study entry and gestational age at delivery. If ultrasound is performed during the screening period, results must be considered for purposes of eligibility determination.

5.2 Enrollment and Follow up Procedures

Up to 72 evaluable HIV uninfected participants (54 non pregnant and 18 pregnant cisgender women) will be enrolled and followed up for 16 weeks: 8 weeks with DOT PrEP, and up to 8 weeks for post drug kinetics sampling – one visit in week 9 and an additional visit within weeks 10 to 16 (i.e. 2 visits in total). Enrollment must occur within 30-day screening window. An eligible participant who is unable to schedule a study entry visit within the 30-day screening window must be re-screened prior to entry into the study. All enrolments will be scheduled on specific days to avoid confusion in implementing the proposed dosing and sampling scheme. Study entry visit (enrollment) through week 8 visit will include an adverse effect questionnaire along with medical and medication review. Safety labs and vitals can be ordered as needed at any visit.

5.2.1 Dosing schedule and assessment of compliance

The design of the study is guided by the ~17 day half-life of TFV-DP in DBS, including 8 weeks of dosing to achieve approximately 90% of steady-state, weekly sampling at convenience times (i.e., rather than troughs), and three dosing groups (2, 4, and 7 doses per week) to demonstrate dose-proportionality. All cisgender women will be followed for 8 weeks with DOT PrEP to establish steady state in DBS and WB, and up to 8 weeks for two post-DOT PrEP sampling visits. We have chosen a dosing frequency scheme for non-daily doses to directly replicate the STRAND DOT study schedule based on the ~17 day half-life of TFV-DP in DBS: The 2 doses per week will be taken on Monday and Tuesday, and the 4 doses per week on Monday, Tuesday, Thursday, and Friday. This schedule will permit simultaneous evaluation of concentrations derived from intermittent and day-by-day dosing. The operationalization of specific days for dose taking can be adjusted prior to study start while maintaining the randomized adherence patterns (frequency of dosing taking) to permit efficient study operations and to minimize the burden on study participants. This is possible due to TFV-DP long half-life and planned sampling at steady state. Therefore, any chosen days would still be appropriate.

All doses will be witnessed, as described above, in person by a study personnel in private room at the Thika clinic or other predetermined location to maximize convenience for the participant. On dosing visits, the participant will take the dose by mouth and study personnel will ensure the tablet is swallowed by

asking the participant to show that their mouth is empty. Flexible clinic scheduling will be available to promote high rates of follow-up and compliance and minimize participant's burden. Where dosing cannot be witnessed in person, dosing will be witnessed by video communication via WhatsApp, Zoom, FaceTime, Skype, or other video-streaming device that is agreed upon by the participant and study personnel will be used. Only cisgender women with an active smart phone will be enrolled in this pharmacological study to permit offsite observation of dose taking for the required DOT PrEP. Participants will be supported with commensurate data/internet bundles. A one-month supply of back-up medication will be provided to participants at study enrollment to be used if participant is unable to complete visit at the clinic. All these doses taken offsite will still be directly observed by study staff by live video streaming and participants will be asked to open their mouth afterwards to confirm swallowing. Dosing may occur at variable times within the assigned dosing day. All doses will be taken without regard to food, but meal information will be collected with each dosing event.

Study personnel will use a dosing log to document directly observed doses. Participants will be given a detailed calendar that shows scheduled dosing days. This schedule will also be reinforced when study personnel interact with participants.

5.2.2 Protocol pharmacology sampling visit schedule

On protocol-specified clinic visits (described below and Table 5), a blood sample will be collected from an arm vein for plasma/WB/RBC/DBS/PBMC isolation and safety laboratories. Hair will be cut from the scalp for comparison with other drug measures. Adverse events will be assessed. All protocol-specified clinic visits are detailed below. Each protocol-specified clinic visit will have a window of up to three days for sample collection (up to a week for hair).

Clinic visit 1: Study entry visit (must be within 30 days after screening visit)

- Medical, sexual, contraception, antenatal and vaccine history review
- Obtain study blood work: WB, DBS, PBMC, plasma, (pre-drug for endogenous molecules such as nucleosides)
- Urine βHCG
- Study urine: (baseline endogenous molecules such as nucleosides)
- STI tests (CT, GT, syphilis)
- hDNA
- Vaginal swab
- Dispense back up PrEP medication: subject home supply
- Dispense study dosing calendar
- DOT: Witness first PrEP dose

Clinic visit 2: Week 1 Dose 4 visit (for 2 doses per week group this will be 4 days since first dose:

- Study Bloodwork: WB, DBS, PBMC, plasma
- Study urine
- Finger stick for DBS
- Cervicovaginal biopsy: Random **subset (50%) of non-pregnant cohort only**
- Vaginal swab
- Medical, sexual, contraception, antenatal and vaccine history review
- AE assessment
- Concomitant Medication Review
- DOT

Clinic visit 3: Week 1 (Dose 7) visit:

- Study bloodwork: WB, DBS, PBMC, plasma
- Study urine
- Finger stick for DBS
- Vaginal biopsy: **Random subsect (50%) of non-pregnant cohort only**
- Vaginal swab
- Blood work: hDNA for those who consent to drug transport gene testing.
- Medical, sexual, contraception, antenatal and vaccine history review
- AE assessment
- Concomitant Medication Review
- DOT

Clinic visit 4: Week 2 (Dose 14) visit:

- Study bloodwork: WB, DBS, PBMC, plasma
- Study urine
- Medical, sexual, contraception, antenatal and vaccine history review
- AE assessment
- Concomitant Medication Review
- DOT

Clinic visit 5: Week 3 (Dose 21) visit:

- Study bloodwork: WB, DBS, PBMC, plasma
- Study urineMedical, sexual, contraception, antenatal and vaccine history review
- AE assessment
- Concomitant Medication Review
- DOT

Clinic visit 6: Week 4 (Dose 28) visit:

- HIV rapid test
- Urine βHCG
- Study bloodwork: WB, DBS, PBMC, plasma
- Study urine
- Vaginal biopsy: **Non-pregnant cohort only**
- Vaginal swab
- Hair collection
- Urine pregnancy test
- Medical, sexual, contraception, antenatal and vaccine history review
- AE assessment
- Concomitant Medication Review
- DOT
- Finger stick for DBS

Clinic visit 7: Week 5 (Dose 35) visit:

- Study bloodwork: WB, DBS, PBMC, plasma
- Study urine
- Medical, sexual, contraception, antenatal and vaccine history review

- AE assessment
- Concomitant Medication Review
- DOT

Clinic visit 8: Week 6 (Dose 42) visit:

- Study bloodwork: WB, DBS, PBMC, plasma
- Medical, sexual, contraception, antenatal and vaccine history review
- AE assessment
- Concomitant Medication Review
- DOT
- Study urine

Clinic visit 9: Week 7 (Dose 49) visit:

- Study bloodwork: WB, DBS, PBMC, plasma
- Study urine
- Medical, sexual, contraception, antenatal and vaccine history review
- AE assessment
- Concomitant Medication Review
- DOT

Clinic visit 10: Week 8 (Dose 56) visit:

- HIV rapid test
- Urine βHCG
- Study bloodwork: WB, DBS, PBMC, plasma
- Finger stick for DBS
- Study urine
- Vaginal biopsy: **Non-pregnant cohort only**
- Vaginal swab
- Hair collection
- Safety Labs: Creatinine
- Medical, sexual, contraception, antenatal and vaccine history review
- AE assessment
- Concomitant Medication Review
- DOT

Post DOT PrEP visits (Week 9): One visit (Clinic visit #11) within week 9, at least 48 hours after the last DOT PrEP dose.

- Study bloodwork: plasma, WB, DBS, PBMC
- Study urine

Post DOT PrEP visits (Week 10-16): One visit (Clinic visit #12) at least 7 days after week 9 visit (i.e., the visit can occur any time between 2-8 weeks after the last DOT F/TAF PrEP which is about week 10 and week 16 since enrollment)

- Study bloodwork: plasma, WB, DBS, PBMC
- Study urine
- Hair collection

5.2.3 Clinical Evaluations/Procedures

Scheduled of protocol visits and procedures are outlined in Table 5. At the screening visit a qualified study clinician will perform a history and physical for each participant. A complete medication history will be obtained including all drug allergies, immunization history, prescription, over the counter, herbal treatments, contraception use history. Vital signs will include height, weight, blood pressure, pulse, respiratory rate and temperature.

5.2.3.1 Specimen collection

All pharmacological samples must be collected as per protocol-specified schedule. Each protocol-specified pharmacological sampling visit will have a window of up to 3 days (up to week for hair) for sample collection. The following pharmacological samples will be collected as follows:

Blood collection

A blood sample will be obtained at protocol specified visits to process plasma, whole blood, DBS, and PBMCs. On a given study day blood collection, up to 21-mL draw with no planned timing relationship with dosing. The blood will be collected from an arm vein by trained clinic staff according to study site policies and procedures.

<u>The schedule of protocol visits and procedures are outlined in Table 5.</u>

Table 5: Schedule of protocol visit schedule and procedures.

	Screening	Entry	DOT PrEP Period (weeks)									Post DOT Period	
Timeline (weeks)			W1		W2	W3	W4	W5	W6	W7	W8	W9	W10-16
			Day 4	Day 7									
Clinic Visits # / Procedure		1	2	3	4	5	6	7	8	9	10	11	12
Informed consent	x												
Medical, sexual, contraception, antenatal history review, vaccine history	x	x	x	x	x	x	x	x	x	x	x		
Physical and obstetric exam [a]	x												
Fetal ultrasound for pregnant women*	x												
Dispense back up PrEP medication[e]		x											
Issue medical calendar		x											
AE assessment		x	x	x	x	x	x	x	x	x	x		
Safety Labs [a,c]	x	[x]									x		
Urine βHCG [a]	x	[x]					x				x		
HBS Ag	x												
HIV-1 rapid test [a]	x	[x]					x				x		
Hair collection							x				x		x
hDNA		x		x									
Study Bloodwork [d] (WB, plasma, DBS, PBMC)**		x	x	x	x	x	x	x	x	x	x	x	x
Vaginal tissue- non-pregnant only[f]			x	x			x				x		
Vaginal swab		x	x	x			x				x		
DBS by fingerstick			x	x			x				x		
Study Urine [d]		x	x	x	x	x	x	x	x	x	x	x	x
Concomitant Medication Review	x	x	x	x	x	x	x	x	x	x	x		
STI testing		x											
DOT[b]		x	x	x	x	x	x	x	x	x	x		

[x] Indicated if screening occurred more 30 days before planned enrollment. *The screen and entry visits must be ≤ 30 days apart.*
**If women has no record of fetal ultrasound for the current pregnancy, ultrasound must be conducted before enrollment.*
***Blood works for pregnancy women only at week 0, 1, 2, 4, 6, 8, 9*
a. Safety labs or vitals can be performed as needed at any time
b. DOT performed if applicable per randomization schedule

c. Protocol-specified safety labs include: CBC, HBAg, Creatinine clearance, in addition to monthly HIV testing and urine pregnancy tests.
d. Study Bloodwork: WB, DBS, PBMC, plasma and all available blood cell types; Study Urine: endogenous molecules such as nucleosides.
e. Study medications will be dispensed as described
f. Random subset (50%) on day 4 and the remaining subset (50%) on day 7

Vaginal swab and tissue

We will obtain vaginal swab for all cisgender women and, in non-pregnant cisgender women, vaginal tissue i.e., only non-pregnant cisgender women will have tissue biopsy. Obtaining vaginal swabs does not impose unnecessary risks in pregnancy and does not stimulate labor. Vaginal biopsies will be taken with a 2.3 x 4.2-mm Tischler gold-plated gynecological forceps. Biopsies will be immediately placed in a pre-weighed and pre-labeled cryovial, subsequently reweighed and immediately stored at -80°C until analysis[59].

Justification for vaginal tissue sampling: Vaginal mucosa is the tissue of relevance to the acquisition of HIV infection for cisgender women. The planned sampling schedule will help to define early drug kinetics and how soon drugs accumulate in vaginal tissue following PrEP initiation in first the week until steady states. To minimize risk associated with this procedure and burden on cisgender women, we will ensure each woman will have only one biopsy in a 4 week-period by sampling separate cisgender women at day 4 and day 7 (Week 1); Thus in week1, only 50% non-pregnant cisgender women will have tissue sample obtained at day 4 and the other half will be sampled day 7. Sample collection at weeks 4 and 8 will be obtained from all non-pregnant cisgender women.

Urine testing

Urine samples will be obtained at periodic intervals for future testing to help refine models for prediction of adherence in cisgender women using urine. The participant will be asked to void a small amount of urine into a sterile specimen container per standard procedures.

Hair collection

A small amount of hair (about 50-100strands) will be collected from the occipital portion of the scalp refine models for prediction of long-term adherence in cisgender women using hair. The hair is cut from as close to the scalp as possible with scissors. The hair that is cut will be taken from underneath the top layer of hair to minimize the ability to see where the hair was cut from. Measuring TFV/FTC exposure in small hair samples is feasible and acceptable in Kenya PrEP trials[55].

Blood for DNA

Blood for DNA will be obtained at the week 1 visit for those who consent for sample storage for future testing. Genomic DNA will be isolated using a commercially available kit, according to standard manufacturer protocol. The DNA will be for pharmacogenetic studies of protein drug transporters described in this protocol and not for whole genome sequencing. Only gene DNA analysis related to antiretroviral drug disposition or treatment response (drug transporters) will be evaluated. An addendum consent will be used to obtain informed consent for future testing.

5.3 Procedures for participants identified as HIV infected

This study may identify persons who become infected with HIV 1 (expected to be very rare) during follow-up of enrolled HIV negative participants. Study staff will provide participants with their HIV test results in the context of post-test counseling. A blood sample will be obtained for HIV drug resistance testing conducted through the standard national GEMS protocol for drug resistance surveillance. These results will be communicated to participants as soon as they are available. Persons identified as HIV infected during the study will be initiated on free HIV treatment and will also be referred to nearby HIV care services for follow up at completion of this trial. For participants who are pregnant and become infected

with HIV during study follow-up, every effort will be made to facilitate immediate access to programs for preventing mother-to-child HIV transmission for appropriate antiretroviral treatment to reduce the probability of HIV transmission from mother to child.

5.4 Procedures for follow up and care for pregnant cisgender women after DOT PrEP

Pregnant cisgender women will be followed closely for any adverse events and pregnant outcomes. Routine health services including counseling for prevention of maternal to child HIV transmission will be provided at Thika clinic during the study period or referred to health facility of their choice.

At each scheduled pharmacological visit, antenatal records will be reviewed, and assessment of fetal heart tones will be conducted. After 8 weeks of DOT PrEP, pregnant cisgender women will be linked to continue PrEP at the clinic of their choice, including at the Thika site clinic itself, permitting seamless access to PrEP for and continuation for HIV prevention after the study. After 8 weeks of DOT PrEP, if a pregnant cisgender woman chooses not to continue with PrEP for protection against HIV, they may have two additional visits after their last PrEP dose – one visit at week 9 and a second visit within weeks 10 to 16. In the event of pregnancy loss during study follow up, study drug procedures and follow up will be terminated and women will be linked to appropriate clinical standard care including continued access for PrEP. After completing DOT phase of study, pregnant women will have observation visits until end of the study or up to the infant's first birthday (12 months) after delivery whichever occurs first to obtain pregnancy outcomes (i.e., term live birth, preterm birth, birth defects or pregnancy loss) and infant growth outcomes (i.e., weight, head circumference and height). Post DOT maternal/infant visit will 1 (i.e., birth visit), 6, and 12 months. Medical and medication history at birth will be reviewed and will consist of information recorded on medical records at birth as well as information obtained at the Birth Visit (Month 1 post-delivery).

The following should be source documented as part of medical and medications history at the Birth Visit:
Pregnancy/birth outcomes: Term live birth, preterm birth, birth defects or pregnancy loss, Sex at birth
Birth length, weight, and head circumference at birth (obtain from medical records; these measurements will also be performed by study staff at the infant Birth Visit), Apgar scores at one and five minutes (obtained from medical records)
Infant growth outcomes: obtained at 1, 6 and 12 months will include: Length, weight, and head circumference.

6 SAFETY MONITORING AND ADVERSE EVENT REPORTING

6.1 Safety Monitoring

Close cooperation between the Protocol Chair, study site Investigator(s), DAIDS Medical Officer, protocol Biostatistician, and other study team members will be necessary in order to monitor participant safety and to respond to occurrences of toxicity in a timely manner.

The study site Investigators are responsible for continuous close monitoring and management of AEs in conjunction with IoRs. The study site is required to have detailed SOPs describing methods for AE reporting and toxicity management to ensure that AEs are reported and managed in accordance with the protocol and for alerting the Protocol Safety Review Team (PSRT) if unexpected concerns arise. The team will have regularly scheduled conference calls during the period of study implementation, and additional ad hoc calls will be convened if required.

A sub-group of the Protocol Team, including the Protocol Chair and co-Chair, DAIDS Medical Officer, site clinicians, and the Seattle-based Study safety monitor will serve as members of the PSRT. The PSRT provides support to site clinicians regarding individual participant clinical management (e.g., questions related to eligibility, toxicity management, clinical holds of study drug, permanent discontinuations, etc.).

The Seattle-based Study safety monitor will review all safety data. The Seattle-based Study safety monitor will prepare routine study conduct and safety reports for the PSRT, which will meet by conference call approximately every 6 months or as needed.

6.2 Clinical Data Review

A multi-tiered safety review process will be followed for the duration of this study. The study site investigators are responsible for the initial evaluation and reporting of safety information at the participant level, and for alerting the PSRT if unexpected concerns arise.

Participant safety data will also be monitored by the Seattle-based Study safety monitor, who review incoming safety data for completeness and consistency on an ongoing basis. Events identified as questionable, inconsistent, or unexplained will be queried for verification. AE reports submitted in an expedited manner to the DAIDS Safety Office will be forwarded to the DAIDS Medical Officer for review.

The Seattle-based Study safety monitor will prepare routine study conduct and safety reports for the PSRT which will meet by conference call approximately every 6 months and will review accrual and retention data, as well as safety data. More frequent or *ad hoc* reviews of safety data may be conducted by the PSRT as needed. A recommendation to stop the trial may be made by the PSRT at any such time that the team agrees an unacceptable type and/or frequency of AEs has been observed. If at any time a decision is made to discontinue the study product in all participants, DAIDS will notify appropriate regulatory agencies, as well as the site IoRs, who will notify the responsible IRBs expeditiously.

6.3 Adverse Event Definition and Reporting

An AE is defined as any untoward medical occurrence in a clinical research participant administered an investigational product and which does not necessarily have a causal relationship with the study product (TDF/FTC). As such, an AE can be an unfavorable or unintended sign (including an abnormal laboratory finding, for example), symptom or disease temporally associated with the use of an investigational product, whether or not considered related to the product.

Study participants will be provided a 24-hour telephone number and contact information and instructed to contact the study clinician to report any AEs they may experience. For life-threatening events, they will also be instructed to seek immediate emergency care. Where feasible and medically appropriate, participants will be encouraged to seek evaluation where the study clinician is based, and to request that the clinician be contacted upon their arrival. With appropriate permission of the participant, whenever possible, records from all non-study medical providers related to AEs will be obtained and required data elements will be recorded on study CRFs. All participants reporting an AE will be followed clinically, until the AE resolves (returns to baseline) or stabilizes.

Study site staff will document in source documents and the appropriate e-CRF AEs (Grade 1 and higher clinical AEs, as well as Grade 2 and higher laboratory AEs, and any AE (clinical or laboratory) that leads to a study product hold (temporary or permanent) will be captured on AE e-CRFs) reported by or observed in enrolled (defined as after randomization has occurred) study participants regardless of severity and presumed relationship to study product. AE severity will be graded per the DAIDS Table for Grading Adult and Pediatric Adverse Events, Version 2.1 corrected, July 2017.

6.4 Relationship to Study Product

Relatedness is an assessment made by a study clinician of whether or not the event is related to the study product. The relationship of all AEs to the study product will be assessed per the clinical judgment of the investigator based on the package insert and investigator's brochure, and as defined in Version 2.0, January 2010 (or most current version) of the DAIDS EAE Reporting Manual, which is available on the DAIDS Regulatory Support Center (RSC) website at https://rsc.niaid.nih.gov/clinical-research-

sites/manual-expedited-reporting-adverse-events-daids. ‘Not related’ will be noted for AEs among participants who do not accept PrEP.

6.5 Grading Severity of Events

The Division of AIDS Table for Grading the Severity of Adult and Pediatric Adverse Events, Corrected Version 2.1, July 2017, will be used for determining and reporting the severity of adverse events. The DAIDS grading table is available on the DAIDS RSC website at https://rsc.niaid.nih.gov/clinical-research-sites/daids-adverse-event-grading-tables.

6.6 Serious Adverse Event (SAE)

The Serious Adverse Event (SAE) Reporting Category, as defined in Version 2.0 (or latest version) of the Manual for Expedited Reporting of Adverse Events to DAIDS, will be used for this study. Only SAEs deemed related to the study product must be reported in an expedited fashion to DAIDS.

All SAEs as defined by ICH guidelines regardless of relationship to the study agent(s) will be reported and are as follows:

- Results in death,
- Is life-threatening,
- Requires inpatient hospitalization or prolongation of existing hospitalization,
- Results in persistent or significant disability/incapacity, or
- Is a congenital anomaly/birth defect

Important medical events that may not be immediately life-threatening or result in death or hospitalization but may jeopardize the patient or may require intervention to prevent one of the outcomes listed in the definition above. Such determination may be made through medical or scientific judgment. Examples include the following: intensive treatment in an emergency room or at home for allergic bronchospasm; blood dyscrasias or convulsions that do not result in hospitalization; development of drug dependency or drug abuse; etc.

6.7 Discontinuation of Study Medication

Subjects may withdraw from the study at any time. Any participant who prematurely discontinues PrEP (for toxicity or pregnancy) should be followed for study duration. Subjects who become pregnant (non-pregnant cohort) or who acquire HIV infection will have study drug discontinued but will be followed to determine the pregnancy outcome.

6.8 Discontinuation of study drug with toxicity

Grade I or II toxicity will be managed in consultation with a clinician on the protocol. The clinician may add follow up testing and evaluations, as medically indicated. Study drug can be continued except as noted below.

Grade III toxicities will be communicated to the PSRT and managed under the direction of a study physician. Follow up testing and evaluations will be added, as medically indicated. The toxicity will be evaluated for subject safety. If the adverse event may put the subject at increased risk, or jeopardizes the study’s intent, even if not related with study drug, the subject will discontinue study drug. The PSRT will decide whether study drug can be restarted. If the adverse event is deemed not to be a significant health risk, the PSRT may recommend continuing study drug with close monitoring.

Grade IV toxicities will be communicated to the PSRT and managed under the direction of a study physician. If a grade IV event is related to the study drug, the participant will permanently discontinue the study drug and will be followed as medically indicated. If the grade IV is not related to the study drug, the PSRT will recommend whether study drug can be continued with close monitoring, or permanently/temporarily discontinued.

Participants who discontinue drug permanently due to a study drug-related AE will be followed until AE resolves or stabilizes and then the subject will be discontinued from the study.

6.8.1 AE Grades 1 or 2

Grade I or II toxicity will be managed in consultation with a clinician on the protocol. The clinician may add follow up testing and evaluations, as medically indicated. Study drug can be continued except as noted below.

6.8.2 AE Grade 3

FTC/TDF may be continued at the directed by the site investigator if a Grade 3 toxicity is considered to be unrelated to the study medication. Study medication will be temporarily withheld if Grade 3 toxicity is considered to be related to the study medication. After a Grade 3 toxicity returns to Grade 1, the participant can be reintroduced to medication after consultation with the site investigator, DAIDS Medical Officer, and other members of the PSRT. If a Grade 3 toxicity recurs and is considered to be related to study medication, FTC/TDF will be permanently discontinued.

6.8.3 Grade 4

For all Grade 4 laboratory-identified or clinical toxicities, FTC/TDF will be withheld unless it is determined to be not related. If a Grade 4 laboratory toxicity is not confirmed by repeat testing, it should be managed per algorithm for the new toxicity grade. Participants with Grade 4 AEs will be followed until the event resolves to baseline or stabilizes. Study drug may be restarted in the event of the resolution of a Grade 4 AE back to Grade 1, after consultation with the site investigator, DAIDS Medical Officer, and other members of the PSRT. If the toxicity recurs to Grade 3 or higher after FTC/TDF is restarted and is considered to be related to FTC/TDF, the study medication will be permanently discontinued.

6.8.4 Creatinine Clearance

Participants who enroll with a Grade 2 CrCl will discontinue PrEP only if there is an increase in severity (to Grade 3 or 4). If the calculated creatinine clearance is <60mL/min, it should be confirmed ideally within approximately one week of the receipt of the results. Study drug should be halted temporarily awaiting confirmatory testing. The PSRT should be consulted for further guidance about restarting study drug for participants who fail to have a confirmed test within two weeks of receiving the initial result._If the calculated creatinine clearance is confirmed to be <60 mL/min, the study drug must be temporarily discontinued. The participant will be monitored as deemed clinically necessary in consultation with the PSRT until level returns to baseline (screening value) or stabilizes.

6.9 Expedited Adverse Event (EAE) Reporting to DAIDS

Requirements, definitions, and methods for expedited reporting of adverse events are outlined in Version 2.0 (or latest version) of the DAIDS EAE Manual, which is available on the DAIDS RSC website at https://rsc.niaid.nih.gov/clinical-research-sites/manual-expedited-reporting-adverse-events-daids

The DAIDS Adverse Event Reporting System (DAERS), an internet-based reporting system, must be used for EAE reporting to DAIDS. In the event of system outages or technical difficulties, EAEs may be submitted via the DAIDS EAE form. For questions about DAERS, please contact NIAID CRMS Support at CRMSSupport@niaid.nih.gov. Site queries may also be sent with the DAERS application itself.

If the site has difficulty accessing the DAERS website, sites will submit EAEs by documenting the information on the current DAIDS EAE form. This form is available on the RSC website: https://rsc.niaid.nih.gov/clinical-research-sites/manual-expedited-reporting-adverse-events-daids. For questions about EAE reporting, please contact the RSC at DAIDSRSCSafetyOffice@tech-res.com.

6.10 Reporting Requirements for this Study

The study agents for the purposes of SAE/EAE reporting are part of a fixed dose combination tablet of FTC/TDF (also outlined in Section 4).

For each study participant, the SAE/EAE reporting period begins at Enrollment (Day 0) and ends when the participant's follow-up in the study ends (at the Week 9 Visit). All reportable SAEs occurring during the study reporting period will be reported to the principal investigator and for EAEs to the DAIDS RSC Safety Office in an expedited manner, within three reporting days of site awareness of the events (see definition in Appendix D of the DAIDS EAE Manual). After the study has ended, sites must report Suspected Unexpected Serious Adverse Reaction (SUSARs) as defined in Version 2.0 of the DAIDS EAE Manual if the study site becomes aware of the event on a passive basis, i.e., from publicly available information. Site staff will also report information regarding SAEs to their IRB or other local regulatory agencies in accordance with all applicable regulations and local IRB requirements.

6.11 Social Impact Reporting

It is possible that participants' involvement in the study could become known to others, and that a social impact may result (i.e., because participants could be perceived as being HIV-infected or at risk or "high risk" for HIV infection). For example, participants could be treated unfairly, or could have problems being accepted by their families and/or communities. A social impact that is reported by the participant and judged by the IoR/designee to be serious or unexpected will be reported to the responsible site's IRBs at least annually, or according to their individual requirements. Social impacts will be collected and reported on CRFs during regular visits. In the event that a participant reports a social impact, every effort will be made by study staff to provide appropriate care and counseling to the participant as necessary, and/or referral to appropriate resources for the safety of the participant. The study site will provide such care and counseling in accordance with standardized guidance in the SSP Manual. While maintaining participant confidentiality, study site may engage their CAB in exploring the social context surrounding instances of social impacts, to minimize the potential occurrence of such an impact.

7 STATISTICAL CONSIDERATIONS

7.1 Review of Study Design

An open-label, randomized, three-arm, directly observed therapy, pharmacologic study. HIV-uninfected non-pregnant cisgender women will be randomly assigned 1:1:1 to 1 of 3 dosing frequencies of DOT TDF/FTC PrEP: 2, 4, or 7 doses/week to help differentiate poor and modest from perfect. An additional contemporaneous cohort of pregnant to receive daily dosing will also be recruited to evaluate the impact of pregnancy on blood and cellular drug levels. We will measure drug concentrations in blood, vaginal fluid, and tissue during the study.

7.2 Study endpoints

Aim 1: Mean, variance, and dose proportionality for TFV-DP in plasma, DBS, and PBMC for 2, 4, 7of DOT TDF-FTC dosing per week.

Aim 2: Prediction model of adherence rate by level of TFV-DP in DBS.

7.3 Data Analysis

7.3.1 Primary outcomes

<u>Primary outcome 1:</u> The goal is to quantify the effect of dose on steady state TFV-DP in DBS and PBMC based on an incomplete block design. Css (drug concentration at steady state) will likely require a normalizing log transform, such that geometric means and CIs will be reported. Dose proportionality will be assessed using the power model (on the natural log scale) $\ln(Y_{ijk}) = \mu + S_i + \beta\log(Dk) + \varepsilon_{ijk}$ (Eqn. 1).[73] Where Y_{ijk} is the response, Css, for the kth dose (k=2, 4, and 7 doses per week), ith subject (i=1…54); μ is the overall mean, Si is a random subject effect, and ε_{ik} is random error. Dose proportionality dictates that $\beta=1$ for dose-dependent parameters and we will assume dose proportionality if the 90% CI is contained within the limits (0.8, 1.25). We will use the observed week 8 concentrations for the dose-proportionality model. Week 8 is approximately 90% of steady state (3.3 * 17-day half-life = 8 weeks). The SAS

mixed procedure will be utilized for model (Eqn 1), again assuming a random subject effect. For the primary analysis, we assume no effect of 'non-daily dosing' regimens; although this will be examined in a secondary analysis. If dose proportionality is not shown, we will examine proportionality separately for each of the ''non-daily dosing' dosing regimens. If dose proportionality is still not demonstrated when adherence regimens are examined separately, we will consider dose as a categorical predictor. Change in objective function and/or AIC will be used to compare models. This approach will be used to further probe dose proportionality and the final model(s) will be used as a basis for simulation studies. Additionally, we will fit a model with following first-order kinetic equation to the TFV-DP data: Ct = Css * (1-e-k*t), where Ct is the TFV-DP at time t (i.e, day 4, weeks 1, 2, 3, 4, 5, 6, 7, 8), Css is the fitted concentration at the steady-state plateau and k is the fitted elimination rate constant, and half-life is ln (2)/k. The fitted Css will be used in the dose-proportionality model. These approaches use non-compartmental and a simple one-compartment model, respectively. Finally, the percentage of dose can be used as a regressor because the concentration at steady-state is directly proportional to dose frequency and TFV-DP clearance [Css=(dose rate)/CL]. Time post dose does not have to be accounted for because it does not impact the TFV-DP concentration given the 17-day half-life. Importantly, we have used these approaches to assess dose proportionality in two similarly designed studies that have been published. [https://pubmed.ncbi.nlm.nih.gov/32539288/.[74-78]

Primary outcome 2: The goal is to identify TFV-DP cut points associated with adherence levels. For the primary analyses, we will attempt to discriminate adherence for 2, 4 and 7 dosing based upon TFV-DP. As above, we will combine the "non-daily dosing'" regimens. Linear mixed models with random intercepts, 25th percentiles, and receiver operating characteristics curves will be used to generate predicted concentration thresholds for varying dosing frequency (ranging from 2 to 7 doses/week) for each matrix-analyte pair. Analyses will be adjusted for age, contraception use, study arm, BMI, eGFR, and hematocrit.

7.3.2 Secondary outcomes

1. Examine models to predict non-daily adherence based on TFV/FTC and TFV-DP in DBS.

   We assume the modeling approaches of Aim-1. We will first consider a saturated model allowing a separate mean for each dose/adherence group. The results will then be compared to a reduced model with a class variable indicating non-daily dosing groups" or 100% dosing (that is, the effect of "non-daily dosing" adherence does not differ by dose groups). Drug concentration predictors of "non-daily dosing" will focus on TFV-DP in relationship to other drug moieties such as FTC-TP and FTC and TFV. For instance, the ratio of TFV-DP to FTC-TP may be used as a predictor of "2" versus "4 dosing per week. A proportional odds logistic approach which allows for an ordinal outcome and extensions which control for correlations between repeated measurements on a subject will be utilized in SAS or R statistical software. In addition, we will employ PK models (see Aim-1) to simulate TFV-DP to FTC-TP ratios for other dose regimens and estimate cut points for predicting non-daily dosing patterns.

2. To quantify the effect of pregnancy on the benchmark concentrations of TFV, TFV-DP, FTC, and FTC-TP in pregnant cisgender women using daily DOT FTC-TDF PrEP.

   Using modeling approaches, we will compare the benchmark concentrations of TFV, FTC, TFV-DP, and FTC-TP between pregnant cisgender women using daily DOT FTC-TDF PrEP. Analyses will be stratified by age and pregnancy gestation.

3. Examine relationships among drug concentrations in plasma, RBC, DBS, and PBMC.

   We will use mixed and non-linear mixed effects models to determine if concentrations in various matrices are related.

4. Evaluate the influence of demographics, biological) on drug concentrations.

Kruskal-Wallis parametric test will be used to compare demographics and key study variables (e.g., age, pregnancy, mean corpuscular volume and HCT, contraception use, study arm, BMI, eGFR). Secondary analyses are considered hypothesis generating and will not correct for multiple comparisons.

5. Compare drug concentrations in DBS from fingerstick versus transferring blood from blood tubes.

   Since these are paired DBS samples, we will compute the ratio of TFV-DP in the two samples and a mixed model on the natural log of the ratio will be utilized to determine if CI is within 0.8 and 1.25, indicating equivalence. Bland-Altman analyses will also be evaluated.

7.4 Sample Size Considerations

The overall sample size was chosen to have up to 18 cisgender women in each relevant group (overall, n=54 non-pregnant cisgender women, 18 pregnant cisgender women) sufficient to provide new information on PrEP use and to balance feasibility, efficiency, and provide robust TDF/FTC data for cisgender women including in pregnancy. The sample size for drug concentration parameters is based on ensuring precision in the estimates to accurately describe the concentration kinetics of TDF/FTC in pregnant and non-pregnant cisgender women and is consistent with multiple DOT PrEP studies done in US populations[41, 42]. The precision desired is a mean TFV-DP in DBS at steady state within ±15% of the true population mean. The mean (± standard deviation) TFV-DP at steady-state in HIV-uninfected US men following directly observed doses of 300 mg TDF/200mg FTC once daily for 12 weeks is 1605 ± 405 fmol/punch [coefficient of variation (CV) of 25.2%] (*Anderson et al*)[41]. Based on this variability and assuming average TFV-DP results are normally distributed, to be 90% confident that the DBS TFV-DP sample mean is within 15% (=60.8 fmol/punch) of the true mean (concentrations at week 8 of DOT dosing or 1445 fmol/punch which is 90% of steady-state concentration at 12 weeks is 1605 fmol/punch), a sample size of 15 participants is required for non-pregnant cisgender women and, with conservative assumptions, 15 per group for pregnant cisgender women. With planned 18 cisgender women per group for this study, even 10% non-completion rate at 8 weeks of DOT therapy, we will have ample data to estimate steady-state means to within 10% in non-pregnant cisgender women and in pregnant cisgender women.

7.5 Randomization and Blinding Procedures

The study design for non-pregnant cohort will be an open-label, randomized study with three arms. The randomization assignments cohort will be made at enrollment if the participant is eligible and agrees to enroll. Randomization will be according to the PrEP dosing frequency and vaginal tissue collection plan (either sampling day 4 or day 7) 1:1:1 fashion programmed by the study statistician and generated from REDcap. Randomization will be done at enrollment.

The allocation of participants for the 3 study arms will be the following:

	2 doses per week	4 doses per week	7 doses per week	Total
**Total**	**18**	**18**	**18**	**54**

The study design for the pregnant cohort will be an open-label, non-randomized study with only DOT PrEP daily dosing regimen (7 doses per week).

7.6 Planned Interim Analyses

Because the study is non-therapeutic and relatively small (N<100) the study team will monitor for safety as described above, but not efficacy. Thus, drug concentration outcomes will not be part of the assessment as samples will be batched tested at the end of the primary data collection. At least one Formal Interim Analysis at 50% enrollment (overall or 50% recruitment for pregnant cohort, whichever occurs first) to review data related to patient safety, including baseline characteristics, creatinine

clearance, HIV infections, and pregnancy outcomes (preterm birth, pregnancy loss, birth defects). The study team will carefully document any adverse events in pregnant cisgender women or infants for careful review.

8 HUMAN SUBJECTS CONSIDERATIONS

8.1 Ethical Review

This protocol and the template informed consent form contained in Appendix and any subsequent modifications — will be reviewed and approved by the DAIDS Prevention Science Review Committee with respect to scientific content and compliance with applicable research and human subjects' regulations.

The protocol, site-specific informed consent form, participant education and recruitment materials, and other requested documents — and any subsequent modifications — also will be reviewed and approved by the University of Washington Human Subjects Ethical Committee and the Scientific and Ethics Review (SERU) Committee of the Kenya Medical Research Institute responsible for oversight of research conducted at the study site.

Subsequent to initial review and approval, the responsible IRBs/ECs will review the protocol at least annually. The Investigator will make safety and progress reports to the IRBs/ECs at least annually, and within three months of study termination or completion. These reports will include the total number of participants enrolled in the study, the number of participants who completed the study, all changes in the research activity, and all unanticipated problems involving risks to human subjects or others. The study site is responsible for the submission of continuing review to the DAIDS Protocol Registration Office, in accordance with the current DAIDS Protocol Registration Policy and Procedure Manual.

8.2 Informed Consent

In obtaining and documenting informed consent, the investigators will comply with all applicable local and/or domestic regulatory requirements and will adhere to Good Clinical Practices and to all ethical principles from the Declaration of Helsinki. A trained research staff person will conduct the informed consent process. Written informed consent will be obtained individually from each participant. Staff will explain the study, read the consent form, and answer any questions the participant may have. Consent information will describe the purpose of the study, the procedures to be followed, and the risks and benefits of participation, in accordance with all applicable regulations. We will translate the forms into the local language (Kiswahili) and verify the accuracy of the translation by performing an independent back-translation. The consent process will occur either in English or the local language (Kiswahili) depending on the participant's preference. We will perform paper-based and/or electronic-based consenting of participants. For the electronic version, we will program the complete consent form with signature pages onto tablets and participants will read along on the tablet during the consenting process. The study team member conducting consent will allow the participant time to ask any questions that they may have before signing the consent form. After we have ensured that participants have read and understood the consent forms, we will ask them to append their signature on the tablet. The signature will be stored electronically. In addition, any participant who wants their consent form to take home will have a paper copy printed and given to them. At each visit after enrollment, participants will have the opportunity to ask questions and the study staff will remind them their participation is voluntary. Participants will be informed that they may withdraw from any of the research procedures at any time, without it affecting the care they receive at the clinic

8.3 Voluntary Participation

The consenting process will emphasize and give information that study participation is voluntary and that the participant can withdraw from the study at any time without losing the care they would otherwise receive.

8.4 Potential Risks to Human Subjects

Safety assessment will be measured in all participants: serious adverse events (SAEs), related Grade 3 and 4 AEs and discontinuations, and a side effects. The medical risks of blood collection and vaginal tissue collection are small, with some anticipated mild discomfort from vaginal speculum insertion, and venipuncture. Participants may become embarrassed, worried, or anxious while undergoing pelvic exams for collection of samples.

Risks related to non-daily PrEP dosing

Participants will be randomized to one of 3 directly observed dosing patterns: 2, 4 or 7 TDF/FTC doses per week. Only daily PrEP is recommended by WHO and the Kenya national guidelines for protection from vaginal HIV exposure and we have carefully considered the risks associated with non-daily in planning this proposal. We have carefully considered the risk associated with using non-daily dosing for PrEP (i.e., 2 or 4 doses per week) in non-pregnant cisgender women and the potential for risk of HIV infection for those non-daily dosing. The non-daily dosing is needed to help investigate and demonstrate dose proportionality of TFV and TDF-DP concentrations. The adherence-concentration thresholds derived from this dosing frequency will be important to estimate efficacy levels derived from varying adherence patterns, including suboptimal dosing. As detailed in eligibility section, only cisgender women at low risk of HIV based on Kenya guidelines will be enrolled and cisgender women will receive full package of HIV prevention, including risk reduction counseling, condoms and STI treatment at each visit. We will also perform monthly HIV testing. We will use Kenya national guidelines to guide identification of cisgender women at elevated risk of HIV to guide recruitment into this study. Each visit will include risk-reduction counseling for all cisgender women. All participants in this study will have HIV test at baseline and then every 4 weeks and PrEP will be immediately discontinued in case of seroconversion.

Risks related to TDF/FTC PrEP

Study medication risks and side effects related to PrEP include: occurring in a minority of individuals taking PrEP - gastrointestinal intolerance, such as nausea, diarrhea or vomiting, flatulence typically during the first month after PrEP initiation; rare but serious side effects of lactic acidosis/ severe hepatomegaly with steatosis, renal impairment, including cases of acute renal failure and Fanconi's syndrome (renal tubular injury with severe hypophosphatemia), increase in bone metabolism leading to osteopenia, and hypersensitivity reaction. The risks of side effects of antiretroviral treatment (ART) are similar to those of PrEP and also rare but serious complications such as lactic acidosis.

Risks from vaginal biopsy

Vaginal tissue biopsy is an in invasive procedure and we have carefully considered the risk involved including some discomfort, pain and potential for bleeding and burden on cisgender women. Sampling vaginal tissue is important to fully characterize HIV protection in cisgender women since it is the tissue of relevance to the acquisition of HIV infection for cisgender women and will help to define early drug kinetics and how soon drugs accumulate in vaginal tissue from PrEP initiation until steady state. To minimize risks, we have carefully considered sampling schedule. We have carefully considered our sampling schedule to minimize risk associated with this procedure and burden on cisgender women: 1) Only non-pregnant cisgender women will provide samples 2) Each woman will provide only one biopsy in a 4-week period by sampling unique cisgender women (i.e. for each planned tissue sampling visits (i.e., separate cisgender women each at day 4, Week 1, 4, and 8).

Social harm and stigma risks

We have considered the risk of social harm related to both DOT PrEP use and frequent clinic visits, including risks of depression/anxiety and disclosure and stigma. Our extensive experience with successful and safe longitudinal follow-up of cisgender women at risk mitigates some of this risk, and we will measure social harms and provide/refer for services when harms are reported. Participants also may become embarrassed, worried, or anxious while completing surveys or interviews regarding sexual behavior and acceptability. Counseling will be provided by clinical study staff who have been trained in specific issues related to STI/HIV risk, PrEP, STI/HIV acquisition, and care of HIV infected cisgender women, including stigma, blame, methods to avoid transmission, and available support services.

Behavior disinhibition risks

The potential for behavior disinhibition may exist as a result of PrEP and is an important outcome, especially given the potential for some participant to receive less than daily dosing of PrEP. We will use brief standardized questionnaires, which we have used for prior studies in cisgender women to explore partner number, sexual frequency, and condom use over time.

Risks of TDF/FTC PrEP use in Pregnancy

There may be risks when taking any drug in pregnancy including for TDF/FTC PrEP. However, PrEP is safe for use in pregnant cisgender women and approved in Kenya as one option for protection against HIV acquisition. TDF and FTC have been used for a long time for treatment in HIV-uninfected pregnant cisgender women and there is no current evidence that PrEP harms babies of women who use it during pregnancy, but it recognized that additional data is needed still helpful to verify this. Pregnancy is associated with gastro-intestinal symptoms (e.g., nausea, vomiting) which are similar to PrEP-related symptoms and therefore use of PrEP in pregnancy could potentially exacerbate these symptoms.

Risk of related to vaginal swabs in pregnancy

Although there is a theoretical possibility of risk of performing vaginal swabs in pregnancy (e.g., precipitating labor), the likelihood of happening is very low with cotton swabs planned for this study and we take confidence from the experience with studies involving pregnant cisgender women at Thika site with similar procedures (Table 4 in the protocol).Importantly, these risks will be discussed with the participants and they will be asked to inform the clinical research team if any of these occur. If a patient develops serious side effects from the medication, then they will be asked to stop the study.

Privacy and confidentiality risks

Although study site will make every effort to protect participant privacy and confidentiality, it is possible that participants' involvement in the study could become known to others. For example, participants could be treated unfairly or discriminated against, or could have problems being accepted by their families and/or communities. We have extensive experience with the strategies to minimize the potential for social harms in populations participating in HIV prevention studies.

Risks from other study procedures

Blood for safety labs and study pharmacological samples will be collected from each subject during the study period. Blood will be collected from an arm vein and by fingerstick. Risks of blood draws include pain when the needle or lancet pierces the skin, bruising, and/or infection. The blood volumes are well below the maximal volumes set for blood donations and clinical research in 9 weeks. Approximately 100 strands of hair will be cut from the occipital portion of the scalp on multiple occasions. This could leave a noticeable spot where the hair was cut from.

8.5 Approaches to Minimize Risks

Protection of risks related to non-daily PrEP dosing

We have carefully considered the risk associated with using non-daily dosing for PrEP (i.e., 2 or 4 doses per week) in non-pregnant cisgender women and the potential for behavior disinhibition as a result of PrEP is an important outcome. We will make all effort to enroll only healthy cisgender women volunteers deemed at low risk for HIV. We will use Kenya national guidelines as well as published HIV risk score (Voice risk) to guide identify cisgender women who may be at elevated risk of HIV to guide recruitment into this study —those found to at elevated risk for HIV will be immediately linked for PrEP and other prevention services here at the Thika site or at the participants' clinic of choice. Each visit will include risk-reduction counseling for all cisgender women. All participants in this study will have HIV test at baseline and then every 4 weeks and PrEP will be immediately discontinued in case of HIV seroconversion. Non-pregnant cisgender women identified to be at elevated risk of acquiring HIV through screening will be immediately linked for PrEP at study clinic site itself or at participant's clinic of choice.

Protection against risks related to TDF/FTC PrEP

All participants will have HIV testing, creatinine testing, hepatitis B, and urine pregnancy testing at baseline. Only cisgender women with creatinine clearance >60 mL/min and uninfected with hepatitis B will be enrolled. We are testing volunteers for HBV-infection to protect subjects from HBV-associated hepatitis flares when the drugs are withdrawn. Breastfeeding will not be allowed to prevent risks to nursing infants. We are excluding subjects who have serious underlying illnesses such as cancer and heart disease. All clients will be counseled about potential side effects at enrollment and each follow up visit and they will be reminded that the side effects are self-limiting within 1-2 weeks. Subjects will be followed with clinical laboratories at entry and 8 weeks (serum creatinine, complete hematology, monthly pregnancy urine test) and more frequently with interviews to assess tolerability and safety over the course of the study, including during directly observed dosing. Participants will be tested for HIV infection at any point during the study as needed, and prior to and at the final visits of the dosing periods.

In summary, the most likely risks associated with TDF-FTC in HIV-negative persons appear to be reversible gastrointestinal complaints, headache, and/or weight decrease. These side effects are most likely mild and reversible.[79] There is a remote risk of serious or fatal side effects including renal failure, lactic acidosis with hepatomegaly and steatosis, bone pathology, hypophosphatemia, hepatitis, pancreatitis, and there are possible side effects not listed here, or possible unforeseen long-term complications. We believe the risk of these serious side effects is remote given the rarity of the events during long-term treatment and PrEP in previous studies. We have designed the inclusion and exclusion criteria to exclude subjects who might be at higher risk of these toxicities. We have also designed a monitoring strategy for early detection of side effects and for quick study drug discontinuation, if needed, to protect subjects.

Protection of risk related to vaginal tissue biopsy

We have also carefully considered our sampling schedule to minimize risk associated with vaginal tissue biopsy and burden on cisgender women and will take the following measures: 1) Only low HIV risk non-pregnant cisgender women will provide vaginal tissue samples 2) Each woman will provide only one biopsy in a 4-week period by sampling separate cisgender women during the first week (i.e. ~half on day 1 and the other half on day 7, ensuring sufficient time before week 4 sample. All non-pregnant cisgender women will provide week 4 and 8. Cisgender women will be counseled to avoid sex in ~7 days after biopsy.

Protection against risks in pregnancy

To ensure an ethical approach for provision of PrEP in pregnancy (i.e., only exposing PrEP to those who

want and might benefit from it), all pregnant cisgender women will be at high risk for HIV and will receive only daily PrEP dosing (7 doses per week). HIV risk and eligibility will be determined according to the Kenya guidelines for PrEP. Pregnant cisgender women will receive only daily PrEP dosing (7 doses per week). After 8 weeks of DOT PrEP, pregnant cisgender women will be linked to continue PrEP at the clinic of their choice, including at the Thika site clinic itself, permitting seamless access to PrEP and continuation after the study. For the initially non-pregnant cisgender women, if they become pregnant, study drug will be discontinued unless they have an indication for PrEP for which daily dosing will be will provided. Cisgender women will be followed to obtain pregnancy outcomes until end of study.

Reducing social harm risks

In addition to protecting the privacy of participants, as described above, we will monitor for social harms. Social harms will be recorded on separate case report forms. Severe unexpected social harms will be considered unexpected problems and reported to the IRB according to local regulations. Social harms will be included on summary reports for the PSRT, as described in this protocol.

Reducing risks behavior disinhibition

We will use brief standardized questionnaires, which we have used for prior studies in cisgender women to explore partner number, sexual frequency, and condom use over time. Each visit will include risk-reduction counseling for all cisgender women and cisgender women will be provided with condoms. Importantly, only cisgender women at low risk of HIV will be enrolled in the non-pregnant cohort which has potential for assignment for non-daily dosing.

Reducing risks from other study procedures

The blood will be collected from an arm vein, and/or by finger stick, by trained staff at the study site according to standard medical procedures. Hematology will be monitored. The hair that is cut will be taken from underneath the top layer of hair to minimize the ability to see where the hair was cut from and to avoid sun-bleached hair.

All participants in this study will have HIV test at baseline and then every 4 weeks. All data received at the University of Washington will be labeled only with participant ID number and no identifying information will be transmitted. The study team has extensive experience with counseling about HIV risk, PrEP (and ART), and strategies for HIV prevention in general. In addition to PrEP and STI treatments, condoms will be provided to all participants by the study site and will follow Kenya clinical guidelines. Counseling about safe sex and medication adherence for prevention will include messaging describing the benefits of all strategies, based on evolving available data and national policies for HIV prevention.

For data collection, standardized questionnaires will be used that will include questions on sensitive topics, including sexual behavior, and stigma. We have extensive experience with these questionnaires from our prior studies and the expertise and counseling resources required to attend to study participants. We have extensive experience with management of potential social harms, through our prevention studies. The risks from the anticipated activities will be no greater than in our previous studies; in fact, given the proven prevention benefits of PrEP and now normalized national roll-out in Kenya, risks are anticipated to be less than in some of our prior studies.

8.6 Benefits.

Participants will benefit from ongoing access to PrEP as now part of standard clinical care. However, the participants or others may benefit in the future from information learned in this study, including contribution to the understanding about HIV prevention and effective PrEP use in cisgender women in Africa, which will help others in the future. While enrolled in the study participants will get access to all services available to those not in the study such as access to oral PrEP (which is known to help prevent

HIV infection), counseling for reducing risk of getting HIV from their sexual partner, and provision of free condoms. For care and treatment that is not available at the clinic, study staff will inform and refer participants to other health facilities where care and treatment may be available.

8.7 Treatment for Injury

Participants will be asked to inform the clinic staff if they feel they have been injured because of taking part in the study. Injuries may also be identified during laboratory testing, medical histories, and physical examinations. Treatment for adverse events related to study participation will be provided by the treatment clinic. If treatment is required that is beyond the capacity of the clinic, the clinic staff will refer the participant to appropriate services or organizations that can provide care for the injury.

8.8 Study Records

Study investigators will maintain, and store in a secure manner, complete, accurate, and current study records throughout the study. Study records include administrative documentation and regulatory documentation as well as documentation related to each participant enrolled in the cohort, including informed consent forms, data forms, documentations of all contacts with the participant, and all other source documents.

8.9 Confidentiality

Every effort will be made to protect participant privacy and confidentiality to the extent possible. At the pharmacy, clients will perform HIV self-testing in private space within the pharmacy area to encourage privacy during the self-testing process. Records pertaining to clinical and programmatic provision of PrEP (i.e. not related to research) will remain at the public health clinic and maintained by MOH staff at clinic as per standard of care practices.

All research-related information will be stored securely at research clinic in Thika. Research data collection, administrative forms, laboratory specimens, and other reports will be identified only by a coded number to maintain participant confidentiality. All research records that contain names or other personal identifiers, such as informed consent forms, will be stored separately in locked cabinets at the research clinic in Thika away from study records identified by code number. All local databases will be secured with password-protected access systems and only project staff with permission will have access to them. Data collected or abstracted from clinical forms during the course of this project will be entered electronically on tablets via REDCap collection software and transmitted to aggregate web server via encrypted secure socket layer (SSL) and only accessible by authenticated users. All computers, tablets (used for data collection), and individual study databases will be encrypted, and password protected.

Participants' study information will not be released without their written permission, except as necessary for oversight by:

- Kenya Ministry of Health
- Kenya Medical Research Institute
- University of Washington
- University of Colorado
- US National Institutes of Health/ DAIDS/NIAID
- U.S., local, and international regulatory entities

8.10 Reimbursement

Participants will be reimbursed 400 KSh for their time and effort at each scheduled study visit. All visits that include collection of vaginal biopsies will be reimbursed 2000 KSh and visits that include collection of vaginal swabs only will be reimbursed 1000 KSh.

8.11 Communicable Disease Reporting Requirements

Study staff will comply with all applicable local requirements to report communicable diseases identified among study participants to local health authorities. Participants will be made aware of all reporting requirements during the study informed consent process.

8.12 Reports of Sexual Abuse

Reporting requirements and confidentiality of minor's information should be detailed in site specific consents/assents and SOPs.

8.13 Study Discontinuation

The study also may be discontinued at any time by NIAID, other government or regulatory authorities (OHRP), or site IRBs/ECs.

Subjects may withdraw from the study at any time. Depending on the time point and circumstances of a subject's withdrawal they will be asked to complete/offer an "Early Discontinuation/Exit Visit" to confirm safety and health status as the time of withdrawal. The study investigators can remove a subject at any time if they deem that the study is no longer in the subject's best interest or the subject is not adhering to the protocol. Discontinuation of a subject by the PI/Co-I(s) could be a result of but is not limited to the following:

- Subjects who become incarcerated while on the study.
- who acquire other infectious diseases (i.e. not-HIV) while in the study will be reviewed by the PI/PSRT to confirm subject is still considered low-risk for HIV infection (See Exclusion criterion) This determination will be done by the PI/study physician.
- Subjects who are deemed to be non-compliant with the study medication(s) and/or procedures (e.g. 2 consecutive missed study visits or medication doses) may be discontinued from the study by the PI.
- Permanent study drug discontinuations for toxicity were described above. These participants will be followed until the completion of scheduled protocol study duration.
- Subjects who require an exclusionary medication during the study will be discussed among the PSRT. Participant's continued study involvement will be determined the PSRT and PI's.

The PSRT will be consulted if and when these circumstances arise.

9 LABORATORY SPECIMENS AND BIOHAZARD CONTAINMENT

9.1 Laboratory evaluations and specimen collections

The following safety and monitoring laboratories will be collected at regular intervals during the study as described above: complete hematology panels, pregnancy tests (urine), and HIV (rapid or regular), serum creatinine, (estimated creatinine clearance), and HBV surface Ag test. The HIV and HBV tests will be used to screen HIV-negative volunteers for inclusion in the study. The tests are provided according to established laboratory procedures at site. These clinical laboratories/sites conform to CAP and/or CLIA-regulations, and the results will be posted to the medical record as positive or negative.

All pharmacological samples will be collected, processed, and stored according to standard operating procedures developed by the Colorado Antiviral Pharmacology Laboratory. On each pharmacological sample visit day, samples will be collected without regard to the dosing time within each day (i.e., sampling at convenience times rather than troughs). The only exception will be at the entry visit to be obtained at pre-dose. We will take a comprehensive approach to establish benchmark adherence-concentration thresholds for cisgender women and pregnant cisgender women using daily DOT TDF/FTC

PrEP by collecting samples for pharmacological measurements in multiple biologic matrices: plasma, whole blood, DBS, PBMCs, and vaginal tissue (non-pregnant cisgender women only) and archiving urine and hair samples. All cisgender women (non-pregnant and pregnant) will contribute to this comprehensive assessment during the up to 16 weeks of follow up (8 weeks of DOT PrEP and up to 8 weeks post DOT PrEP for post drug kinetics). Within each group, we will collect pharmacologic samples (plasma, whole blood, DBS, PBMCs) to measure the respective concentrations of TDF/FTC moieties at day 4 and weekly up to 8 weeks with daily DOT PrEP dosing and at week 9 and weeks 10-16 post DOT PrEP period, and in nonpregnant cisgender women, vaginal tissue at day 4, week 1, 2, 4, and 8.

9.2 Specimen preparation, handling, and shipping

All pharmacological samples will be collected, processed, stored, and shipped according to standard operating procedures developed by the Colorado Antiviral Pharmacology Laboratory. Specimens will be collected by skilled study personnel in accordance with local study site policies and procedures. All specimen collections are described in this protocol.

Samples that are shipped to outside entities are regulated by the International Air Transportation Association. Such training and certification of study personnel is handled through the University of Colorado Denver Environmental Health and Safety Division. This training is required on a biannual basis. This protocol follows IATA Guidance 48-DGR when air transport is required.

9.3 Specimen Storage and Possible Future Research Testing

We will store blood, urine, hair, and genital specimens collected in this study for future testing. Study participants will be asked to provide written informed consent for their specimens to be stored after the end of the study for possible future testing related to the study objectives. The specimens of participants who do not consent to long-term storage and additional testing will be destroyed after all protocol-related testing has been completed; sample destruction must be coordinated with the protocol leadership and PSRT.

9.4 Quantification of drug concentrations

Drug concentrations will be determined using analytical methodologies (such as liquid chromatography tandem mass spectrometry (LC/MS) methods) described previously [8, 80] and validated for each biological matrix of origin (plasma, whole blood, DBS, PBMCs) by the Colorado Antiviral Pharmacology Laboratory (CLIA 06D1094710; PI: Dr. Anderson)[81-84]. The CLIA laboratory participates in the NIH-supported Clinical Pharmacology Quality Assurance program of assay method external review and approval and periodic proficiency testing[85]. All assays were validated based on the FDA recommendations for Industry, Bioanalytical Method Validation and met all acceptability criteria. Validation metrics include precision, accuracy, stability, and matrix effects. The laboratory has a long history of innovative and lasting contributions to antiviral clinical-translational research including development and validation of methodology for intracellular TDF-DP and FTC-DP in various cell types from human subjects. Data from this assay was used to support the pharmacology measurements for the iPrEx[8] and DISCOVER studies that led to the FDA-indication of daily TDF/FTC and TAF/FTC PrEP for MSM and the pharmacologic study that developed an estimate of protective concentrations of tenofovir as a surrogate of HIV protection in MSM. We will use the same methods to support the current proposal. These assays have been implemented in a number of preclinical, clinical, and registration trials.

9.5 Biohazard Containment

All study personnel will follow universal precautions, as recommended by the Center for Disease Control and the NIH including the appropriate disposal of needles (etc) and human wastes to prevent the accidental transmission of blood borne pathogens. Training as such will be documented in study personnel's records. Study specific SOPs will outline procedures for safe procurement and handling of study specimens. The Thika Research site, the Colorado Antiviral Pharmacology Laboratory has

biosafety authorization from the University of Colorado. All dangerous goods materials, including diagnostic specimens and infectious substances, will be transported according to instructions detailed in the International Air Transport Association Dangerous Goods Regulations.

10 ADMINISTRATIVE PROCEDURES

10.1 Protocol Registration

Initial Registration of the protocol by the DAIDS Protocol Registration Office (PRO) is required prior to implementation of this protocol. As part of this process, the site must have the protocol and protocol ICF(s) approved, as appropriate, by their local institutional review board (IRB)/ethics committee (EC) and any other applicable regulatory entity (RE). Upon receiving final approval, the site will submit all required protocol registration documents to the DAIDS PRO at the Regulatory Support Center (RSC). The DAIDS PRO will review the submitted protocol registration packet to ensure that all of the required documents have been received. In the case of Initial Registration, site-specific ICFs WILL be reviewed and approved by the DAIDS PRO. The Site will receive an Initial Registration Notification from the DAIDS PRO that indicates successful completion of the protocol registration process. A copy of the Initial Registration Notification should be retained in the site's regulatory files.

Following Initial Registration, any full protocol amendments require submission of a protocol registration packet to the DAIDS PRO as described above; however, the DAIDS PRO **WILL NOT** review and approve site-specific ICFs. The site will receive a Registration Notification when the DAIDS PRO receives a complete registration packet. This study will also be registered on https://www.clinicaltrials.gov.

For additional information on the protocol registration process and specific documents required for initial and amendment registrations, refer to the current version of the DAIDS Protocol Registration Manual, which can be found at
https://www.niaid.nih.gov/sites/default/files/prmanual.pdf
.

10.2 Study activation

Pending successful protocol registration and submission of all required documents, the protocol chair will "activate" a site. Study implementation (screening and enrollment) may not be initiated until a study activation notice is provided to the site by the protocol chair. In addition, if study "activation" is determined to be necessary for any subsequent amendments, study implementation may not be initiated until a study activation notice is provided to the site by the protocol chair.

10.3 Study coordination

Study implementation will be directed by this protocol as well as the SSP manual. The SSP manual — which will contain links to the Requirements for Source Documentation in DAIDS Funded and/or Sponsored Clinical Trials, as well as the DAIDS Manual for Expedited Reporting of Adverse Events to DAIDS, Version 2.0, dated (January 2010) and the DAIDS Toxicity Tables — will outline procedures for conducting study visits; data and forms processing; AE assessment, management and reporting; dispensing study products and documenting product accountability; and other study operations.

Close coordination between protocol team members will be necessary to track study progress, respond to queries about proper study implementation, and address other issues in a timely manner. Rates of accrual, adherence, follow-up, and AE incidence will be monitored closely by the protocol team. The Protocol Co-Chairs, DAIDS Medical Officer, and Protocol Biostatistician will address issues related to study eligibility and AE management and reporting as needed to assure consistent case management, documentation, and information-sharing across sites.

10.3.1 Data storage and management responsibilities

Data will be managed by study personnel at the site and University of Washington. Clinical and baseline data collected during the course of this study will be collected electronically via RedCap data collection

software. Quantitative survey data, clinic and pharmacy records will be abstracted in real time to tablet-based case report forms and entered into an electronic database in RedCap. Automated legal range checks will be programmed to reduce data entry errors and internal quality control reports will be run monthly. Our team has experience using this data capture method in other studies of HIV prevention. Data will be uploaded daily via RedCap Survey and be transported via secure socket layer (SSL) and only accessible by authenticated users. Weekly reports will be generated to monitor study progress and troubleshoot problems. All computers, tablets (used for primary data collection), and individual study databases will be encrypted, and password protected. Participants will be assigned a non-identifiable study code upon enrollment. Study analysts will receive only coded data. The links to patient identifiers will be retained in a password protected file on an encrypted computer.

10.3.2 Source Documents and Access to Source Data/Documents

Source documents will be kept in subject study records (binders). Documentation will be sufficient such that study data can be reconstructed, evaluated, and validated for all clinical activities during the trial. The goals are to ensure that protocol, IRBs, and DAIDS requirements and standards are adhered to and that all data will be verifiable from the written source document and will create an audit trail to verify that data is present, complete and accurate. Source data consists of all information in original records and certified copies of original records. The "ALCOA" method is used to achieve and maintain data quality: Attributable, Legible, Contemporaneous, Original and Accurate.

10.3.3 Data monitoring

The study team will review project progress in the weekly operational meeting and monthly for an all-team meeting; teleconference or internet-based mechanisms (Skype, Zoom) will be used for these meetings. The Seattle-based data manager will conduct data monitoring weekly via routine operational reports to track participant accrual, retention, completion of study procedures, and a check of eligibility criteria for all enrolled participants. Data quality checks will be conducted monthly to identify missing values, inconsistent information, and unacceptable lags between the timing of study visits and data updates to the web-based data entry platform. Twice per year, the PI and a Seattle-based research manager will travel to study site in Kenya to conduct an on-site assessment of data and laboratory quality; site Co-Investigators will perform day-to-day oversight of quality for the project.

The study and updated information will be registered on ClinicalTrials.gov according to UW Office of Sponsored Program and NIH policies.

10.3.4 Quality control and Quality assurance

A program for quality assurance and quality control has been developed as a system of self- monitoring to promote the integrity and quality of the study. The program is detailed in SOP.

10.4 Study monitoring

On-site study monitoring will be performed in accordance with DAIDS policies. Study monitors will visit the site to:

- Verify compliance with human subjects and other research regulations and guidelines;
- Assess adherence to the study protocol, study-specific procedures manual, and local counseling practices; and
- Confirm the quality and accuracy of information collected at the study site and entered into the study database.

Site investigators will allow study monitors to inspect study facilities and documentation (e.g., ICFs, clinic and laboratory records, other source documents, CRFs), as well as observe the performance of study procedures. Investigators also will allow inspection of all study-related documentation by authorized representatives of the UW, NIAID, site IRBs/ECs, US regulatory authorities (OHRP), Kenya MOH, PPB,

and other U.S., local, and international regulatory entities. A site visit log will be maintained at each study site to document all visits.

The International Clinical Research Center (ICRC) at the University of Washington will provide regular study monitoring for the Thika site. The Study Monitor will be a Senior Research Manager at ICRC. The Research Manager will have a graduate degree and will be experienced in site monitoring activities. The site monitor will conduct on site reviews of source documents, participant records, regulatory files, facilities, laboratories and the dispensing pharmacy. The signed informed consent documents will be made available for review for compliance with GCP and the DAIDS policy requirements. Original source documents will be made available to verify all inclusion/exclusion criteria and for compliance with protocol requirements and DAIDS policies for source documentation. Individual participant's source documents will also be available for review and comparison to protocol requirements and the completed case report forms (CRFs). Through this detailed review, monitoring will focus on the following key processes of the study:

- Informed consent process
- Study eligibility criteria met for all participants
- Timely completion of study CRFs
- Sample collection and handling in accordance to Protocol and SOP(s)
- Review of data management procedure
- Reporting of adverse events and protocols violations according to SOP(s)
- Follow up assessments and procedures
- Product accountability
- Regulatory documents on file

Monitoring visits may be conducted on-site or remotely. Remote visits may include remote source document verification using methods specified for this purpose by NIAID. Remote monitoring visits may be performed in place of, or in addition to onsite visits to ensure the safety of study participants and data integrity[86]. The site will make available study documents for site monitors to review utilizing a secure platform that is HIPAA and 21 CFR Part 11 compliant. Potential platform options include: Veeva SiteVault, site-controlled SharePoint or cloud-based portal, direct access to Electronic Medical Record (EMR), and Medidata Rave Imaging Solution. Other secure platforms that are 21 CFR Part 11 compliant may be utilized, as allowed by the DAIDS Office of Clinical Site Oversight (OCSO).

10.5 Protocol Compliance

The study will be conducted in full compliance with the protocol. The protocol will not be amended without prior written approval by the Protocol Chair and DAIDS Medical Officer. All protocol amendments must be submitted to and approved by the relevant IRB(s)/EC(s) and the RSC prior to implementing the amendment.

10.6 Investigator's Records

The Investigator will maintain, and store in a secure manner, complete, accurate, and current study records throughout the study. Under the US Department of Health and Human Services (DHHS) regulations, the Investigator is required to retain all study records relating to research for at least three [3] years after completion of the research, or longer if needed to comply with local regulations.

Completion of a clinical research study occurs when the following activities have been completed:

- All research-related interventions or interactions with human subjects (e.g. when all subjects are off study);

- All protocol-required data collection of identifiable private information described in the IRB/EC-approved research plan;
- All analysis of identifiable private information described in the IRB/EC-approved research plan;
- Primary analysis of either identifiable private or de-identified information.

Study records include administrative documentation — including protocol registration documents and all reports and correspondence relating to the study — as well as documentation related to each participant screened and/or enrolled in the study — including ICFs, locator forms, CRFs, notations of all contacts with the participant, and all other source documents.

10.7 Use of Information and Publications

The Principal Investigator will have oversight of publications and presentations emanating from this study. The Principal Investigator will ensure that authorship and authorship order is equitable with respect to the effort contributed to the work. Contributing effort that justifies authorship includes participation in the design of the study and assays used in the study; participation in carrying out the study; participation in analyzing the data; and participation in writing the manuscript or presentation. Trainees or students who contribute work will be equally eligible for authorship. First authorship will reflect the individual who contributes the most effort and writing to the manuscript. First authorship may be split if two individuals contributed to the effort equally. Senior authorship will reflect the individual who provided the scientific oversight and direction for the manuscript or presentation.

10.8 Resource sharing and dissemination plan

10.8.1 Resource Sharing:

For all data generated during the course of this project, we will follow the prevailing standards and guidelines in documenting and depositing datasets. We will make quality-controlled raw data as well as processed data used in publications available. As described in the grant application, protocols and workflows will be implemented exactly as described and documented such that other groups will be able to precisely reproduce results from the raw data.

The study personnel assigned to the project, including KEMRI-based and University of Washington-based investigators, will disseminate results from this research through presentations at public lectures, scientific institutions and meetings, and/or publication in major journals. The institutions and Principal Investigators will adhere to the NIH Grants Policy on Sharing of Unique Research Resources including the Sharing of Biomedical Research Resources: Guidelines for Recipients of NIH Grants and Contracts on Obtaining and Disseminating Biomedical Research Resources.

10.8.2 Data Sharing Plan

Intellectual property and data generated under this project will be administered in accordance with NIH policies, including the NIH Data Sharing Policy and Implementation Guidance of March 5, 2003. Materials generated under the project will be disseminated in accordance with KEMRI, University of Washington, University of Colorado, and NIH policies. Depending on such policies, materials may be transferred to others under the terms of a material transfer agreement. Access to databases and associated software tools generated under the project will be available for educational, research, and non-profit purposes. Such access will be provided using web-based applications, as appropriate. Publication of data shall occur during the project, if appropriate, or at the end of the project, consistent with normal scientific practices. Research data that documents, supports, and validates research findings will be made available after the main findings from the final research data set have been accepted for publication. Such research data will be modified to prevent the disclosure of personal identifiers to remain in compliance with the Protection of Human Subjects guidelines.

10.8.3 Collaborative research

The proposed project is a collaborative effort between NIH/NAIDS, UW, UC, and KEMRI. The aforementioned institutions will jointly share ownership of the data. Authorship on publications, conference presentations, abstracts and other materials generated from this program will reflect contribution to design, execution and analysis of the program data.

10.8.4 Dissemination plan

Dissemination of study results will follow principles of good participatory practice. Study results will be disseminated through presentations to stakeholders and policymakers and published in conference abstracts and peer-reviewed journals.

We will disseminate the results from this research as broadly as possible. Of note, drafts of publications will be shared with the NIH program office and medical officer prior to submission. First, we will publish our results in Open Access journals, if appropriate. Second, we will post author PDFs of our manuscripts on our respective websites in accordance with the copyright rules of the journals.

10.8.5 Presentations

We expect that all the research personnel will attend national conferences periodically and present the results from this research to the scientific community. Because of the multidisciplinary nature of the work, different group members will present at various conferences, such as the Conference on Retrovirus and Opportunistic Infections, International AIDS Society, and Kenya-based conferences, which focus on the appropriate aspects of our research.

The study team for this award is committed to public dissemination of results of clinical trial, to trial participants, local stakeholders in Kenya, the global scientific community, and US, Kenyan, and global policymakers. Dissemination of study results will follow principles of good participatory practice. The clinical trial will be registered with Clinicaltrials.gov prior to initiation and results will be updated there in a timely fashion. Results will be published in conference abstracts and peer-reviewed journals. Study results will be disseminated through presentations to local stakeholders and policymakers in Kenya, including the Ministry of Health.

10.9 Intellectual property consideration

There is no commercial value for this work. The proposed project is a collaborative effort between NIH/NAID, UW, UC, and KEMRI. All data collected as part of this implementation program will be made available without cost with written request and agreement to the data sharing agreement after completion of primary analyses. The data sharing agreement will ensure commitments to:

- Using the data only for program evaluation purposes and without attempting to identify patients
- Securing the data using appropriate computer technology
- Destroying or returning the data after analyses are completed
- Restrictions on redistribution of the data to third parties
- Proper acknowledgement of the data resource.

11 TIMELINES

We have planned for an efficient time-sensitive four-year project, given the timely nature of the question (Table 7).

Table 7		Project Years	Y1				Y2				Y3			
AIM	MILESTONE	Quarter	Q1	Q2	Q3	Q4	Q1	Q2	Q3	Q4	Q1	Q2	Q3	Q4
Funding	Funding Announced		■											
Protocol Planning	Protocol Development		■	■										
	IRB Submission, SOP development			■	■	■								
Protocol Execution	Enrollment, follow up in DOT study, pharmacologic sample collection					■	■	■	■	■				
	Regulatory application from MOH and IRB to ship samples				■	■	■	■	■					
	Ship DOT pharmacologic samples							■	■	■	■	■		
	Test DOT study samples									■	■	■	■	■
	Statistical analyses and compilation of full spectrum of results													■
Dissemination	Writing and results dissemination													

12 EXPECTED APPLICATION OF RESULTS

The study will be the first to define TDF-PrEP adherence-concentration thresholds in Africa cisgender women, a population facing significant burden of incident HIV infection. Knowledge gained from the proposed study will include key concentration of PrEP medications associated with HIV protection and the required adherence to achieve those concentrations for cisgender women, and optimal dosing for PrEP in at-risk pregnant cisgender women. In addition, data from this work will help to estimate the quantitative concentrations of PrEP medication associated with protection against HIV in cisgender women and adherence required to generate those concentrations. The results of this work will have immediate implications for the global PrEP programs among cisgender women.

13 STUDY LIMITATIONS

The study team recognizes potential limitation of the proposed work which may include: 1) Part of preliminary data are not from DOT studies and that observed effects might be due to correlation between MEMS and blood levels and not related to pharmacologic differences between US male and African cisgender women blood levels. However, we take confidence in fact that our pregnancy data from the same population have been replicated by the IMPAACT 2009 study; 2) Intensive sampling for individual PK parameters is not planned as that is well defined. DBS exhibits a long half-life and is not suited for intensive PK. Nevertheless, plasma steady-state levels while it does not pinpoint the mechanism for PK differences, it will indicate whether or not recent PK exposure differs and by how much. Furthermore, population PK models will be developed to determine individual-level PK in plasma.

14 REFERENCES

1. UNAIDS. UNAIDS data 2018; 2019 [updated 2019; cited 2020 June 15]; Available from: https://www.unaids.org/sites/default/files/media_asset/2019-UNAIDS-data_en.pdf.
2. UNAIDS. THE GAP REPORT; 2015.
3. A. Kamali, M. A. Price, S. Lakhi, E. Karita, M. Inambao, E. J. Sanders, O. Anzala, M. H. Latka, L. G. Bekker, P. Kaleebu, G. Asiki, A. Ssetaala, E. Ruzagira, S. Allen, P. Farmer, E. Hunter, G. Mutua, H. Makkan, A. Tichacek, I. K. Brill, P. Fast, G. Stevens, P. Chetty, P. N. Amornkul, J. Gilmour and I. A. H. P. Partnership. Creating an African HIV clinical research and prevention trials network: HIV prevalence, incidence and transmission. PLoS One. 2015;10(1):e0116100. PMCID: PMC4300215.
4. K. M. De Cock, H. W. Jaffe and J. W. Curran. The evolving epidemiology of HIV/AIDS. AIDS. 2012;26(10):1205-13.
5. A. L. Drake, A. Wagner, B. Richardson and G. John-Stewart. Incident HIV during pregnancy and postpartum and risk of mother-to-child HIV transmission: a systematic review and meta-analysis. PLoS Med. 2014;11(2):e1001608. PMCID: 3934828.
6. K. A. Thomson, J. Hughes, J. M. Baeten, G. John-Stewart, C. Celum, C. R. Cohen, K. Ngure, J. Kiarie, N. Mugo and R. Heffron. Increased Risk of HIV Acquisition Among Women Throughout Pregnancy and During the Postpartum Period: A Prospective Per-Coital-Act Analysis Among Women With HIV-Infected Partners. J Infect Dis. 2018;218(1):16-25. PMCID: PMC5989601.
7. Evidence for Contraceptive Options HIV Outcomes Trial Consortium. HIV incidence among women using intramuscular depot medroxyprogesterone acetate, a copper intrauterine device, or a levonorgestrel implant for contraception: a randomised, multicentre, open-label trial. Lancet. 2019;394(10195):303-13. PMCID: PMC6675739.
8. R. M. Grant, J. R. Lama, P. L. Anderson, V. McMahan, A. Y. Liu, L. Vargas, P. Goicochea, M. Casapia, J. V. Guanira-Carranza, M. E. Ramirez-Cardich, O. Montoya-Herrera, T. Fernandez, V. G. Veloso, S. P. Buchbinder, S. Chariyalertsak, M. Schechter, L. G. Bekker, K. H. Mayer, E. G. Kallas, K. R. Amico, K. Mulligan, L. R. Bushman, R. J. Hance, C. Ganoza, P. Defechereux, B. Postle, F. Wang, J. J. McConnell, J. H. Zheng, J. Lee, J. F. Rooney, H. S. Jaffe, A. I. Martinez, D. N. Burns and D. V. Glidden. Preexposure chemoprophylaxis for HIV prevention in men who have sex with men. N Engl J Med. 2010;363(27):2587-99.
9. J. M. Baeten, D. Donnell, P. Ndase, N. R. Mugo, J. D. Campbell, J. Wangisi, J. W. Tappero, E. A. Bukusi, C. R. Cohen, E. Katabira, A. Ronald, E. Tumwesigye, E. Were, K. H. Fife, J. Kiarie, C. Farquhar, G. John-Stewart, A. Kakia, J. Odoyo, A. Mucunguzi, E. Nakku-Joloba, R. Twesigye, K. Ngure, C. Apaka, H. Tamooh, F. Gabona, A. Mujugira, D. Panteleeff, K. K. Thomas, L. Kidoguchi, M. Krows, J. Revall, S. Morrison, H. Haugen, M. Emmanuel-Ogier, L. Ondrejcek, R. W. Coombs, L. Frenkel, C. Hendrix, N. N. Bumpus, D. Bangsberg, J. E. Haberer, W. S. Stevens, J. R. Lingappa, C. Celum and Partners PrEP Study Team. Antiretroviral prophylaxis for HIV prevention in heterosexual men and women. N Engl J Med. 2012;367(5):399-410. PMCID: 3920826.
10. M. C. Thigpen, P. M. Kebaabetswe, L. A. Paxton, D. K. Smith, C. E. Rose, T. M. Segolodi, F. L. Henderson, S. R. Pathak, F. A. Soud, K. L. Chillag, R. Mutanhaurwa, L. I. Chirwa, M. Kasonde, D. Abebe, E. Buliva, R. J. Gvetadze, S. Johnson, T. Sukalac, V. T. Thomas, C. Hart, J. A. Johnson, C. K. Malotte, C. W. Hendrix and J. T. Brooks. Antiretroviral preexposure prophylaxis for heterosexual HIV transmission in Botswana. N Engl J Med. 2012;367(5):423-34.
11. K. Choopanya, M. Martin, P. Suntharasamai, U. Sangkum, P. A. Mock, M. Leethochawalit, S. Chiamwongpaet, P. Kitisin, P. Natrujirote, S. Kittimunkong, R. Chuachoowong, R. J. Gvetadze, J. M. McNicholl, L. A. Paxton, M. E. Curlin, C. W. Hendrix and S. Vanichseni. Antiretroviral prophylaxis for HIV infection in injecting drug users in Bangkok, Thailand (the Bangkok Tenofovir Study): a randomised, double-blind, placebo-controlled phase 3 trial. Lancet. 2013;381(9883):2083-90.
12. US Public Health Service. Preexposure Prophylaxis for the Prevention of HIV infection in the United States; 2014 [updated 2014; cited]; Available from: http://www.cdc.gov/hiv/pdf/PrEPguidelines2014.pdf.

13. World Health Organization. Guideline on when to start antiretroviral therapy and on pre-exposure prophylaxis for HIV Switzerland; 2015 [updated 2015 September 2015; cited]; Available from: http://apps.who.int/iris/bitstream/10665/186275/1/9789241509565_eng.pdf?ua=1.
14. Ministry of Health Government of Kenya. Guidelines on the use of antiretroviral drugs for treating and preventing HIV infection in Kenya. 2016 Edition Nairobi, Kenya; 2016 [updated 2016 2016; cited 2019 December 30]; Available from: http://emtct-iatt.org/wp-content/uploads/2016/09/Guidelines-on-Use-of-Antiretroviral-Drugs-for-Treating-and-Preventing-HI....pdf.
15. AVAC. Pre-Exposure Prophylaxis (PrEP) by the Numbers: Efficacy, regulatory approval and more; 2016 [updated 2016; cited]; Available from: https://www.avac.org/sites/default/files/resource-files/prep_BTN_aug2016.pdf.
16. P. M. Murnane, C. Celum, N. Mugo, J. D. Campbell, D. Donnell, E. Bukusi, A. Mujugira, J. Tappero, E. M. Kahle, K. K. Thomas, J. M. Baeten and E. P. S. T. Partners Pr. Efficacy of preexposure prophylaxis for HIV-1 prevention among high-risk heterosexuals: subgroup analyses from a randomized trial. AIDS. 2013;27(13):2155-60. PMCID: PMC3882910.
17. J. M. Marrazzo, G. Ramjee, B. A. Richardson, K. Gomez, N. Mgodi, G. Nair, T. Palanee, C. Nakabiito, A. van der Straten, L. Noguchi, C. W. Hendrix, J. Y. Dai, S. Ganesh, B. Mkhize, M. Taljaard, U. M. Parikh, J. Piper, B. Masse, C. Grossman, J. Rooney, J. L. Schwartz, H. Watts, M. A. Marzinke, S. L. Hillier, I. M. McGowan, Z. M. Chirenje and V. S. Team. Tenofovir-based preexposure prophylaxis for HIV infection among African women. N Engl J Med. 2015;372(6):509-18. PMCID: PMC4341965.
18. L. Van Damme, A. Corneli, K. Ahmed, K. Agot, J. Lombaard, S. Kapiga, M. Malahleha, F. Owino, R. Manongi, J. Onyango, L. Temu, M. C. Monedi, P. Mak'Oketch, M. Makanda, I. Reblin, S. E. Makatu, L. Saylor, H. Kiernan, S. Kirkendale, C. Wong, R. Grant, A. Kashuba, K. Nanda, J. Mandala, K. Fransen, J. Deese, T. Crucitti, T. D. Mastro, D. Taylor and F. E.-P. S. Group. Preexposure prophylaxis for HIV infection among African women. N Engl J Med. 2012;367(5):411-22. PMCID: 3687217.
19. A. van der Straten, L. Van Damme, J. E. Haberer and D. R. Bangsberg. Unraveling the divergent results of pre-exposure prophylaxis trials for HIV prevention. AIDS. 2012;26(7):F13-9. PMCID: 22333749.
20. R. Zash, S. Souda, J. Y. Chen, K. Binda, S. Dryden-Peterson, S. Lockman, M. Mmalane, J. Makhema, M. Essex and R. Shapiro. Reassuring Birth Outcomes With Tenofovir/Emtricitabine/Efavirenz Used for Prevention of Mother-to-Child Transmission of HIV in Botswana. J Acquir Immune Defic Syndr. 2016;71(4):428-36. PMCID: PMC4767604.
21. D. M. Gibb, H. Kizito, E. C. Russell, E. Chidziva, E. Zalwango, R. Nalumenya, M. Spyer, D. Tumukunde, K. Nathoo, P. Munderi, H. Kyomugisha, J. Hakim, H. Grossjurth, C. F. Gilks, A. S. Walker, P. Musoke and o. b. o. t. D. t. team. Pregnancy and Infant Outcomes among HIV-Infected Women Taking Long-Term ART with and without Tenofovir in the DART Trial. PLoS Med. 2012;9(5):e10001217.
22. B. H. Chi, M. Sinkala, F. Mbewe, R. A. Cantrell, G. Kruse, N. Chintu, G. M. Aldrovandi, E. M. Stringer, C. Kankasa, J. T. Safrit and J. S. A. Stringer. Single-dose tenofovir and emtricitabine for reduction of viral resistance to non-nucleoside reverse transcriptase inhibitor drugs in women given intrapartum nevirapine for perinatal HIV prevention: an open-label randomised trial. The Lancet. 2007;370(9600):1698-705.
23. C. E. Ransom, Y. Hou, K. Patel, G. B. Scott, D. H. Watts, P. Williams, G. K. Siberry, E. G. Livingston and P. T. o. I. M. P. A. A. C. T. Group. Infant Growth Outcomes After Maternal Tenofovir Disoproxil Fumarate Use During Pregnancy. J Acquir Immune Defic Syndr. 2013;64(1).
24. S. Flanagan, L. Barnes, J. Anderson and T. Barber. The effect of tenofovir on renal function in HIV-positive pregnant women. HIV Drug Therapy Glasgow Congress. Glasgow, Scotland: Journal of the International AIDS Society; 2014. p. 19694.
25. G. K. Siberry, P. L. Williams, H. Mendez, G. R. Seage, D. L. Jacobson, R. Hazra, K. C. Rich, R. Griner, K. Tassiopoulos, D. Kacanek, L. M. Mofenson, T. Miller, L. A. DiMeglio, D. H. Watts and P. H. A. C. S. (PHACS). Safety of Tenofovir Use During Pregnancy: Early Growth Outcomes in HIV-Exposed Uninfected Infants. AIDS. 2012;26(9):1151-9.
26. L. Wang, A. P. Kourtis, S. Ellington, J. Legardy-Williams and M. Bulterys. Safety of tenofovir during pregnancy for the mother and fetus: a systematic review. Clin Infect Dis. 2013;57(12):1773-81.

27. G. K. Siberry, D. L. Jacobson, H. J. Kalkwarf, J. W. Wu, L. A. DiMeglio, R. Yogev, K. M. Knapp, J. J. Wheeler, L. Butler, R. Hazra, T. L. Miller, G. R. Seage, 3rd, R. B. Van Dyke, E. Barr, M. Davtyan, L. M. Mofenson, K. C. Rich and H. I. V. A. C. S. Pediatric. Lower Newborn Bone Mineral Content Associated With Maternal Use of Tenofovir Disoproxil Fumarate During Pregnancy. Clin Infect Dis. 2015;61(6):996-1003. PMCID: PMC4551007.
28. N. R. Mugo, T. Hong, C. Celum, D. Donnell, E. A. Bukusi, G. John-Stewart, J. Wangisi, E. Were, R. Heffron, L. T. Matthews, S. Morrison, K. Ngure, J. M. Baeten and E. P. S. T. Partners Pr. Pregnancy incidence and outcomes among women receiving preexposure prophylaxis for HIV prevention: a randomized clinical trial. JAMA. 2014;312(4):362-71. PMCID: PMC4362516.
29. R. Beigi, L. Noguchi, T. Parsons, I. Macio, R. P. Kunjara Na Ayudhya, J. Chen, C. W. Hendrix, B. Masse, M. Valentine, J. Piper and D. H. Watts. Pharmacokinetics and placental transfer of single-dose tenofovir 1% vaginal gel in term pregnancy. J Infect Dis. 2011;204(10):1527-31. PMCID: PMC3192189.
30. R. Callahan, K. Nanda, S. Kapiga, M. Malahleda, J. Mandala, T. Ogada, L. V. Damme, D. Taylor and f. t. F.-P. S. Group. Pregnancy and Contraceptive Use Among Women Participating in the FEM-PrEP Trial. Journal of Aquired Immune Deficiency Syndromes. 2015;68(2):196-203.
31. P. L. Vernazza, I. Graf, U. Sonnenberg-Schwan, M. Geit and A. Meurer. Preexposure prophylaxis and timed intercourse for HIV-discordant couples willing to conceive a child. AIDS. 2011;25(16):2005-8.
32. J. Whetham, S. Taylor, L. Charlwood, T. Keith, R. Howell, C. McInnes, E. Payne, J. Home, D. White and Y. Gilleece. Pre-exposure prophylaxis for conception (PrEP-C) as a risk reduction strategy in HIV-positive men and HIV-negative women in the UK. AIDS Care. 2014;26(3):332-6.
33. R. Heffron, N. Mugo, T. Hong, C. Celum, M. A. Marzinke, K. Ngure, S. Asiimwe, E. Katabira, E. A. Bukusi, J. Odoyo, E. Tindimwebwa, N. Bulya, J. M. Baeten, P. Partners Demonstration and E. P. S. T. the Partners Pr. Pregnancy outcomes and infant growth among babies with in-utero exposure to tenofovir-based preexposure prophylaxis for HIV prevention. AIDS. 2018;32(12):1707-13. PMCID: PMC6086376.
34. G. Jourdain, N. Ngo-Giang-Huong, L. Harrison, L. Decker, W. Khamduang, C. Tierney, N. Salvadori, T. R. Cressey, W. Sirirungsi, J. Achalapong, P. Yuthavisuthi, P. Kanjanavikai, O. P. Na Ayudhaya, T. Siriwachirachai, S. Prommas, P. Sabsanong, A. Limtrakul, S. Varadisai, C. Putiyanun, P. Suriyachai, P. Liampongsabuddhi, S. Sangsawang, W. Matanasarawut, S. Buranabanjasatean, P. Puernngooluerm, C. Bowonwatanuwong, T. Puthanakit, V. Klinbuayaem, S. Thongsawat, S. Thanprasertsuk, G. K. Siberry, D. H. Watts, N. Chakhtoura, T. V. Murphy, N. P. Nelson, R. T. Chung, S. Pol and N. Chotivanich. Tenofovir versus Placebo to Prevent Perinatal Transmission of Hepatitis B. N Engl J Med. 2018;378(10):911-23. PMCID: PMC5895092.
35. N. Salvadori, B. Fan, W. Teeyasoontranon, N. Ngo-Giang-Huong, S. Phanomcheong, A. Luvira, A. Puangsombat, A. Suwannarat, U. Srirompotong, C. Putiyanun, T. R. Cressey, L. Decker, W. Khamduang, L. Harrison, C. Tierney, J. A. Shepherd, A. P. Kourtis, M. Bulterys, G. K. Siberry and G. Jourdain. Maternal and Infant Bone Mineral Density 1 Year After Delivery in a Randomized, Controlled Trial of Maternal Tenofovir Disoproxil Fumarate to Prevent Mother-to-child Transmission of Hepatitis B Virus. Clin Infect Dis. 2019;69(1):144-6. PMCID: PMC6579954.
36. AIDSinfo. Recommendations for Use of Antiretroviral Drugs in Pregnant HIV-1 Infected Women for Maternal Health and Interventions to Reduce Perinatal HIV Transmission in the United States. Washington, DC: National Institutes of Health; 2016.
37. S. Benaboud, A. Pruvost, P. A. Coffie, D. K. Ekouevi, S. Urien, E. Arrive, S. Blanche, F. Theodoro, D. Avit, F. Dabis, J. M. Treluyer and D. Hirt. Concentrations of tenofovir and emtricitabine in breast milk of HIV-1-infected women in Abidjan, Cote d'Ivoire, in the ANRS 12109 TEmAA Study, Step 2. Antimicrob Agents Chemother. 2011;55(3):1315-7. PMCID: PMC3067089.
38. M. Mirochnick, B. M. Best and D. F. Clarke. Antiretroviral pharmacology: special issues regarding pregnant women and neonates. Clin Perinatol. 2010;37(4):907-27, xi.
39. N. A. S. C. P. Ministry of Health. Guidelines on use of Antiretroviral Drugs for Treating and Preventing HIV INfection in Kenya 2016. Nairobi, Kenya: NASCOP; 2016 July 2016.
40. WHO. Guideline on when to start antiretroviral therapy and on pre-exposure prophylaxis for HIV. Geneva, Switzerland: World Health Organization (WHO); 2015.

41. P. L. Anderson, A. Y. Liu, J. R. Castillo-Mancilla, E. M. Gardner, S. M. Seifert, C. McHugh, T. Wagner, K. Campbell, M. Morrow, M. Ibrahim, S. Buchbinder, L. R. Bushman, J. J. Kiser and S. MaWhinney. Intracellular Tenofovir-Diphosphate and Emtricitabine-Triphosphate in Dried Blood Spots following Directly Observed Therapy. Antimicrob Agents Chemother. 2018;62(1). PMCID: PMC5740314.
42. A. Y. Liu, Q. Yang, Y. Huang, P. Bacchetti, P. L. Anderson, C. Jin, K. Goggin, K. Stojanovski, R. Grant, S. P. Buchbinder, R. M. Greenblatt and M. Gandhi. Strong relationship between oral dose and tenofovir hair levels in a randomized trial: hair as a potential adherence measure for pre-exposure prophylaxis (PrEP). PLoS One. 2014;9(1):e83736. PMCID: PMC3885443.
43. P. L. Anderson, D. V. Glidden, A. Liu, S. Buchbinder, J. R. Lama, J. V. Guanira, V. McMahan, L. R. Bushman, M. Casapia, O. Montoya-Herrera, V. G. Veloso, K. H. Mayer, S. Chariyalertsak, M. Schechter, L. G. Bekker, E. G. Kallas, R. M. Grant and T. iPrEx Study. Emtricitabine-tenofovir concentrations and pre-exposure prophylaxis efficacy in men who have sex with men. Sci Transl Med. 2012;4(151):151ra25. PMCID: PMC3721979.
44. M. R. Nicol, C. W. Emerson, H. M. Prince, J. A. Nelson, Y. Fedoriw, C. Sykes, E. J. Geller, K. B. Patterson, M. S. Cohen and A. D. Kashuba. Models for predicting effective HIV chemoprevention in women. J Acquir Immune Defic Syndr. 2015;68(4):369-76. PMCID: PMC4334725.
45. A. D. Kashuba, K. B. Patterson, J. B. Dumond and M. S. Cohen. Pre-exposure prophylaxis for HIV prevention: how to predict success. Lancet. 2012;379(9835):2409-11. PMCID: PMC3652584.
46. V. Naranbhai, S. S. Abdool Karim, M. Altfeld, N. Samsunder, R. Durgiah, S. Sibeko, Q. Abdool Karim, W. H. Carr and C. T. team. Innate immune activation enhances hiv acquisition in women, diminishing the effectiveness of tenofovir microbicide gel. J Infect Dis. 2012;206(7):993-1001. PMCID: PMC3501691.
47. E. N. Fish. The X-files in immunity: sex-based differences predispose immune responses. Nat Rev Immunol. 2008;8(9):737-44.
48. T. J. Yi, B. Shannon, J. Prodger, L. McKinnon and R. Kaul. Genital immunology and HIV susceptibility in young women. Am J Reprod Immunol. 2013;69 Suppl 1:74-9.
49. C. Celum, N. Mgodi, L. G. Bekker, S. Hosek, D. Donnell, P. L. Anderson, B. J. Dye, S. R. Pathak, Y. Agyei, J. M. Fogel, M. A. Marzinke, K. Makgamathe, S. Kassim, S. Mukaka, H. Noble, A. Adeyeye, S. Delany-Moretlwe and on behalf of the HPTN 082 Study Team. PrEP adherence and effect of drug level feedback among young African women in HPTN 082. International AIDS Society; 21-24 July 2019; Mexico City, Mexico 2019.
50. J. M. Baeten, R. Heffron, L. Kidoguchi, N. R. Mugo, E. Katabira, E. A. Bukusi, S. Asiimwe, J. E. Haberer, J. Morton, K. Ngure, N. Bulya, J. Odoyo, E. Tindimwebwa, C. Hendrix, M. A. Marzinke, N. C. Ware, M. A. Wyatt, S. Morrison, H. Haugen, A. Mujugira, D. Donnell and C. Celum. Integrated delivery of antiretroviral treatment and pre-exposure prophylaxis to HIV-1-serodiscordant couples: a prospective implementation study in Kenya and Uganda. PLoS Med. 2016;13(8):e1002099.
51. M. Pyra, P. Anderson, J. E. Haberer, R. Heffron, C. Celum, S. Asiimwe, E. Katabira, N. R. Mugo, E. A. Bukusi and J. M. Baeten. Tenofovir-Diphosphate as a Marker of HIV Pre-exposure Prophylaxis Use Among East African Men and Women. Front Pharmacol. 2019;10:401. PMCID: PMC6478885.
52. M. Pyra, P. L. Anderson, C. W. Hendrix, R. Heffron, K. Mugwanya, J. E. Haberer, K. K. Thomas, C. Celum, D. Donnell, M. A. Marzinke, E. A. Bukusi, N. R. Mugo, S. Asiimwe, E. Katabira, J. M. Baeten and T. Partners Demonstration Study. Tenofovir and tenofovir-diphosphate concentrations during pregnancy among HIV-uninfected women using oral preexposure prophylaxis. AIDS. 2018;32(13):1891-8. PMCID: PMC6061961.
53. Y. Lu, C. Celum, A. Wald, J. M. Baeten, F. Cowan, S. Delany-Moretlwe, S. E. Reid, J. P. Hughes, E. Wilcox, L. Corey and C. W. Hendrix. Acyclovir achieves a lower concentration in African HIV-seronegative, herpes simplex virus 2-seropositive women than in non-African populations. Antimicrob Agents Chemother. 2012;56(5):2777-9. PMCID: PMC3346629.
54. B. M. Best, S. Burchett, H. Li, A. Stek, C. Hu, J. Wang, E. Hawkins, M. Byroads, D. H. Watts, E. Smith, C. V. Fletcher, E. V. Capparelli, M. Mirochnick, P. International Maternal and A. C. T. P. T. Adolescent. Pharmacokinetics of tenofovir during pregnancy and postpartum. HIV Med. 2015;16(8):502-11. PMCID: PMC4862736.
55. S. M. Baxi, A. Liu, P. Bacchetti, G. Mutua, E. J. Sanders, F. M. Kibengo, J. E. Haberer, J. Rooney, C. W. Hendrix, P. L. Anderson, Y. Huang, F. Priddy and M. Gandhi. Comparing the novel method of assessing PrEP

adherence/exposure using hair samples to other pharmacologic and traditional measures. J Acquir Immune Defic Syndr. 2015;68(1):13-20. PMCID: PMC4262724.
56. Peter L. Anderson, Lynda Stranix-Chibanda, S. H. Sharon Huang, Deborah Kacanek, Teacler Nematadzira, Frank Taulo, Violet Korutaro, Clemensia Nakabiito, Masebola Masenya, Kathryn Lypen, Nahida Chakhtoura, Hans M. Spiegel, Benjamin H. Chi and the IMPAACT 2009 team. TFV-DP IN DBS FOR PREGNANT/POSTPARTUM ADOLESCENT AND YOUNG WOMEN ON PrEP IN AFRICA. Conference on Retroviruses and Opportunistic Infections. Boston, MA, March 8-11, 2020.
57. P. L. Anderson, D. V. Glidden, A. Liu, S. Buchbinder, J. R. Lama, J. V. Guanira, V. McMahan, L. R. Bushman, M. Casapia, O. Montoya-Herrera, V. G. Veloso, K. H. Mayer, S. Chariyalertsak, M. Schechter, L. G. Bekker, E. G. Kallas and R. M. Grant. Emtricitabine-tenofovir concentrations and pre-exposure prophylaxis efficacy in men who have sex with men. Sci Transl Med. 2012;4(151):151ra25. PMCID: 3721979.
58. K. K. Mugwanya, C. W. Hendrix, N. R. Mugo, M. Marzinke, E. T. Katabira, K. Ngure, N. B. Semiyaga, G. John-Stewart, T. R. Muwonge, G. Muthuri, A. Stergachis, C. L. Celum and J. M. Baeten. Pre-exposure prophylaxis use by breastfeeding HIV-uninfected women: A prospective short-term study of antiretroviral excretion in breast milk and infant absorption. PLoS Med. 2016;13(9):e1002132. doi: 10.1371/journal.pmed. PMCID: PMC5038971.
59. R. P. Bakshi, J. Breakey, M. Manohar, B. Jois, E. J. Fuchs and M. A. Marzinke. Short Communication: Specimen Processing Impacts Tissue Tenofovir Pharmacokinetic Measurements. AIDS Res Hum Retroviruses. 2018;34(4):354-6. PMCID: PMC5899295.
60. C. Celum, A. Wald, J. R. Lingappa, A. S. Magaret, R. S. Wang, N. Mugo, A. Mujugira, J. M. Baeten, J. I. Mullins, J. P. Hughes, E. A. Bukusi, C. R. Cohen, E. Katabira, A. Ronald, J. Kiarie, C. Farquhar, G. J. Stewart, J. Makhema, M. Essex, E. Were, K. H. Fife, G. de Bruyn, G. E. Gray, J. A. McIntyre, R. Manongi, S. Kapiga, D. Coetzee, S. Allen, M. Inambao, K. Kayitenkore, E. Karita, W. Kanweka, S. Delany, H. Rees, B. Vwalika, W. Stevens, M. S. Campbell, K. K. Thomas, R. W. Coombs, R. Morrow, W. L. Whittington, M. J. McElrath, L. Barnes, R. Ridzon, L. Corey and Partners in Prevention HSV/HIV Transmission Study Team. Acyclovir and transmission of HIV-1 from persons infected with HIV-1 and HSV-2. N Engl J Med. 2010;362(5):427-39. PMCID: PMC2838503.
61. K. H. Fife, K. Mugwanya, K. K. Thomas, J. M. Baeten, C. Celum, E. Bukusi, G. de Bruyn, A. Mujugira, B. Vwalika, A. Wald, J. R. Lingappa and H. S. V. H. I. V. T. S. T. Partners in Prevention. Transient Increase in Herpes Simplex Virus Type 2 (HSV-2)-Associated Genital Ulcers Following Initiation of Antiretroviral Therapy in HIV/HSV-2-Coinfected Individuals. J Infect Dis. 2016;213(10):1573-8. PMCID: PMC6281349.
62. K. Mugwanya, J. M. Baeten, N. R. Mugo, E. Irungu, K. Ngure and C. Celum. High-dose valacyclovir HSV-2 suppression results in greater reduction in plasma HIV-1 levels compared with standard dose acyclovir among HIV-1/HSV-2 coinfected persons: a randomized, crossover trial. J Infect Dis. 2011;204(12):1912-7. PMCID: PMC3247811.
63. World Health Organization. WHO Technical brief: Preventing HIV during pregnancy and breastfeeding in the context of pre-exposure prophylaxis (PrEP) Geneva; 2017 [updated 2017; cited]; Available from: http://apps.who.int/iris/bitstream/10665/255866/1/WHO-HIV-2017.09-eng.pdf.
64. N. Davies and R. Heffron. Global and national guidance for the use of pre-exposure prophylaxis during peri-conception, pregnancy and breastfeeding. Sex Health. 2018;15(6):501-12.
65. K. A. Sullivan and A. D. Lyerly. Ethical considerations in developing an evidence base for pre-exposure prophylaxis in pregnant women. Reprod Health. 2017;14(Suppl 3):171. PMCID: PMC5751520.
66. R. Heffron, D. Donnell, H. Rees, C. Celum, N. Mugo, E. Were, G. de Bruyn, E. Nakku-Joloba, K. Ngure, J. Kiarie, R. W. Coombs, J. M. Baeten and Partners in Prevention HSV/HIV Transmission Study Team. Use of hormonal contraceptives and risk of HIV-1 transmission: a prospective cohort study. Lancet Infect Dis. 2012;12(1):19-26. PMCID: 3266951.
67. R. Heffron, N. Mugo, E. Were, J. Kiarie, E. A. Bukusi, A. Mujugira, L. M. Frenkel, D. Donnell, A. Ronald, C. Celum, J. M. Baeten and Partners PrEP Study Team. Preexposure prophylaxis is efficacious for HIV-1 prevention among women using depot medroxyprogesterone acetate for contraception. AIDS. 2014;28(18):2771-6. doi: 10.1097/QAD.0000000000000493. PMCID: 4266161.
68. N. R. Mugo, T. Hong, C. Celum, D. Donnell, E. A. Bukusi, G. John-Stewart, J. Wangisi, E. Were, R. Heffron, L. T. Matthews, S. Morrison, K. Ngure, J. M. Baeten and Partners PrEP Study Team. Pregnancy incidence and

outcomes among women receiving preexposure prophylaxis for HIV prevention: a randomized clinical trial. JAMA. 2014;312(4):362-71. doi: 10.1001/jama.2014.8735. PMCID: 4362516.
69. N. R. Mugo, K. Ngure, M. Kiragu, E. Irungu and N. Kilonzo. The preexposure prophylaxis revolution; from clinical trials to programmatic implementation. Curr Opin HIV AIDS. 2016;11(1):80-6.
70. K. K. Mugwanya, D. Donnell, C. Celum, K. K. Thomas, P. Ndase, N. Mugo, E. Katabira, K. Ngure, J. M. Baeten and Partners PrEP Study Team. Sexual behaviour of heterosexual men and women receiving antiretroviral pre-exposure prophylaxis for HIV prevention: A longitudinal analysis. Lancet Infect Dis. 2013;13(12):1021-8. PMCID: 3920826.
71. K. Ngure, R. Heffron, N. Mugo, E. Irungu, C. Celum and J. M. Baeten. Successful increase in contraceptive uptake among Kenyan HIV-1-serodiscordant couples enrolled in an HIV-1 prevention trial. AIDS. 2009;23 Suppl 1(1):S89-95.
72. K. Ngure, R. Heffron, N. Mugo, K. A. Thomson, E. Irungu, N. Njuguna, L. Mwaniki, C. Celum and J. M. Baeten. Feasibility and acceptability of HIV self-testing among pre-exposure prophylaxis users in Kenya. J Int AIDS Soc. 2017;20(1):1-8.
73. V. S. Sethuraman, S. Leonov, L. Squassante, T. R. Mitchell and M. D. Hale. Sample size calculation for the Power Model for dose proportionality studies. Pharm Stat. 2007;6(1):35-41.
74. P. L. Anderson, J. Lamba, C. L. Aquilante, E. Schuetz and C. V. Fletcher. Pharmacogenetic characteristics of indinavir, zidovudine, and lamivudine therapy in HIV-infected adults: a pilot study. J Acquir Immune Defic Syndr. 2006;42(4):441-9.
75. D. A. Kile, S. Mawhinney, C. L. Aquilante, J. E. Rower, J. R. Castillo-Mancilla and P. L. Anderson. A Population Pharmacokinetic-Pharmacogenetic Analysis of Atazanavir. AIDS Res Hum Retroviruses. 2012.
76. J. J. Kiser, C. L. Aquilante, P. L. Anderson, T. M. King, M. L. Carten and C. V. Fletcher. Clinical and genetic determinants of intracellular tenofovir diphosphate concentrations in HIV-infected patients. J Acquir Immune Defic Syndr. 2008;47(3):298-303.
77. J. Rower, Y. Ghodke, B. Klein, J. H. Zheng, L. Bushman, J. Predhomme, J. Lamba and P. L. Anderson. Associations among genetic variants in the cellular pharmacology pathway for zidovudine and lamivudine with intracellular phosphate levels in vivo 19th Conference on Retroviruses and Opportunistic Infections; Mar 5-8; Seattle, WA: 2012.
78. H. Akaike. A new look at the statistical model identification. IEEE Trans. on Automatic Control. 1974;AC-19:716-23.
79. G. Sciences. TRUVADA® (EMTRICITABINE/TENOFOVIR DISOPROXIL FUMARATE) FOR PRE-EXPOSURE PROPHYLAXIS OF HIV-1; 2012 [updated 2012 May 10; cited]; Available from: http://www.fda.gov/downloads/AdvisoryCommittees/CommitteesMeetingMaterials/Drugs/AntiviralDrugsAdvisoryCommittee/UCM303216.pdf.
80. J. H. Zheng, C. Rower, K. McAllister, J. Castillo-Mancilla, B. Klein, A. Meditz, L. A. Guida, J. J. Kiser, L. R. Bushman and P. L. Anderson. Application of an intracellular assay for determination of tenofovir-diphosphate and emtricitabine-triphosphate from erythrocytes using dried blood spots. J Pharm Biomed Anal. 2016;122:16-20. PMCID: 4764437.
81. L. R. Bushman, J. J. Kiser, J. E. Rower, B. Klein, J. H. Zheng, M. L. Ray and P. L. Anderson. Determination of nucleoside analog mono-, di-, and tri-phosphates in cellular matrix by solid phase extraction and ultra-sensitive LC-MS/MS detection. J Pharm Biomed Anal. 2011;56(2):390-401. PMCID: PMC3153375.
82. C. W. Hendrix, B. A. Chen, V. Guddera, C. Hoesley, J. Justman, C. Nakabiito, R. Salata, L. Soto-Torres, K. Patterson, A. M. Minnis, S. Gandham, K. Gomez, B. A. Richardson and N. N. Bumpus. MTN-001: randomized pharmacokinetic cross-over study comparing tenofovir vaginal gel and oral tablets in vaginal tissue and other compartments. PLoS One. 2013;8(1):e55013. PMCID: PMC3559346.
83. M. J. Keller, R. P. Madan, N. M. Torres, M. J. Fazzari, S. Cho, S. Kalyoussef, G. Shust, P. M. Mesquita, N. Louissaint, J. Chen, H. W. Cohen, E. C. Diament, A. C. Lee, L. Soto-Torres, C. W. Hendrix and B. C. Herold. A randomized trial to assess anti-HIV activity in female genital tract secretions and soluble mucosal immunity following application of 1% tenofovir gel. PLoS One. 2011;6(1):e16475. PMCID: PMC3026837.

84. J. H. Zheng, L. A. Guida, C. Rower, J. Castillo-Mancilla, A. Meditz, B. Klein, B. J. Kerr, J. Langness, L. Bushman, J. Kiser and P. L. Anderson. Quantitation of tenofovir and emtricitabine in dried blood spots (DBS) with LC-MS/MS. J Pharm Biomed Anal. 2014;88:144-51. PMCID: PMC3842403.
85. R. DiFrancesco, C. R. Taylor, S. L. Rosenkranz, K. M. Tooley, P. G. Pande, S. M. Siminski, R. W. Jenny and G. D. Morse. Adding value to antiretroviral proficiency testing. Bioanalysis. 2014;6(20):2721-32. PMCID: PMC4296581.
86. FDA Guidance on Conduct of Clinical Trials of Medical Products During the COVID-19 Public. Health Emergency: Guidance for Industry, Investigators, and Institutional Review Boards, March 2020, Updated on January 27, 2021.
; [cited 2021 May]; Available from: Accessed at: https://www.fda.gov/media/136238/download.

15 APPENDIXES— STUDY CONSENT FORM TEMPLATES

Study Screening Informed Consent Form

Version 1.0, May 20, 2021

INVESTIGATORS

Investigator	Title	Institution	Telephone
Kenneth Mugwanya	MBChB, MS, PhD	University of Washington	001 206-520-3886
Nelly Mugo	MBChB, MMed, MPH	Kenya Medical Research Institute	067 2222561
Peter Anderson	PharmD	University of Colorado	001 303-724-6128
Elizabeth Irungu	MBChB, MPH	Kenya Medical Research Institute	067 2222561
Catherine Kiptinness	B. Pharm, MPH	Kenya Medical Research Institute	067 2222561

STUDY LOCATION: The study will be conducted at Thika.

You are being asked to take part in a research study. The box below tells you important things you should think about before deciding to join the study. We will provide more detailed information below the box. Please ask questions about any of the information before you decide whether to participate. You may also wish to talk to others (for example, your family, friends, or your doctor) about this study, before agreeing to join.

Key Information for You to Consider
• **Voluntary Consent**. You are being asked to volunteer for a research study. It is up to you whether you choose to participate or not. There are no penalties and you will not lose anything if you decide not to joint or if after you join, you decide to quit.<br>• **Purpose**. We are doing this research to find out more about the medication called Tenofovir/Emtricitabine (TDF/FTC), which can lower the chances for HIV-uninfected men and women to get the HIV virus. This is also called preexposure prophylaxis or PrEP. We want to learn more about the levels of PrEP in blood and other body parts when used by women.<br>• **Duration.** Your part of the study will last up to 4 months.<br>• **Procedures and Activities.** We will ask you questions about yourself including your medical and sexual history, and some blood samples will be collected. Should you be eligible and agree to participate, we will collect additional samples including blood, hair and fluids from your genital area.<br>• **Risks.** Most studies have some possible harms that could happen to you if you join. In this study, we expect that the primary risks will be some discomfort from sample collection, stress or discomfort from answering survey questions.<br>• **Benefits**. We expect some benefits from this study, as well. For you, we don't think that you may get direct benefit from being in this study. However, we will inform you about any test results that are done that could be useful to your health care.<br>• **Alternatives.** Participation is voluntary and the only alternative is to not participate.

INFORMED CONSENT

We are asking you to volunteer to have screening tests to find out if you are eligible for a research study. This study is open to women who do not have HIV. Before you decide whether to take part in the study, we would like to explain the purpose of the study, the risks and benefits, and what would be expected of you if you agree to be in the study. This study is being coordinated through the Kenya Medical Research Institute in Kenya and the University of Washington located in Seattle, Washington, USA. The study is being funded through the US National Institutes of Health in Washington, DC, USA. If you decide to participate in the research study, you will be asked to sign this consent form or make your mark in front of a witness. We will give you a copy of this form if you choose to have. This consent form might contain some words that are unfamiliar to you. Please ask us to explain anything you may not understand.

PURPOSE OF THE STUDY

Medication called Tenofovir/Emtricitabine (TDF/FTC) can lower the chances for HIV-uninfected men and women to get the HIV virus. This is called preexposure prophylaxis or PrEP. PrEP medication is safe to use, does not lead to serious problems during pregnancy and there is no current evidence that PrEP harms babies of women who use it during pregnancy. PrEP is approved by the World Health Organization and the Kenya Ministry of Health as one option to prevent getting HIV. Research studies have also shown that PrEP works only when taken as prescribed. Other research studies have shown that the levels of drugs in blood after taking PrEP may be different in men and women, especially during pregnancy. The purpose of this study is to find out more about the levels of PrEP in blood and other body parts when used by women.

YOUR PARTICIPATION IS VOLUNTARY

This consent form gives information about the study that we will discuss with you. Before you learn about the study procedures, it is important that you know the following:

- You do not have to be in this study if you do not want to join.
- You may decide not to take part in the study, or to withdraw from the study at any time.
- If you decide not to take part in the study, you can still join another research study later, if one is available and you qualify.

SCREENING PROCEDURES

Screening procedures will begin today, after you read, discuss, and sign or make your mark on this form. The screening procedures will include the following:

- The study staff will check your temperature and ask you questions about COVID-19 symptoms and people you been in physical contact with, to see if you have been exposed to COVID-19. Should the staff determine that you have been exposed, they will follow the recommended Ministry of Health guidelines in referring you for healthcare. We will reschedule your visit for another time based on these guidelines.
- The study staff will ask you where you live and other questions about your sexual practices, medical history and, pregnancy and contraceptive use intentions.
- A clinician will perform a physical and obstetric exam, urine pregnancy test and review your medication and vaccine history.
- If you are pregnant, we will review your records for any recent ultrasounds that you have done. If you have not done an ultrasound, we will do one for you free of charge.

- We will counsel you about HIV and other infections passed during sex, and how to avoid these infections.
- Even if you have recently been tested for HIV, we will need to repeat the HIV test today as part of screening for the study. The study staff will talk with you about the HIV test, what it may mean to know your HIV test results. You must receive your HIV test results to be in the research study.
- If you do not have HIV infection:
  - We will ask you questions about your medical and sexual health history, and pregnancy intentions.
  - You will be asked to give us permission to obtain a blood sample for laboratory tests. No more than 21 ml (about one and quarter tablespoons) will be collected for all the screening tests, including the HIV test. Some of the blood sample will be used to test for hepatitis B infection and some will be used to test the function of your kidneys.

The study staff will fully explain the study to you and answer any questions you have. You must have at least one other visit here at the clinic to learn about the study. If you decide to take part in the research study and found to meet all the requirements for the study, you will be asked to sign another consent form at another visit.

RISKS AND/OR DISCOMFORTS

You may feel discomfort or pain when your blood is drawn. You may feel dizzy or faint. You may have a bruise where the sterile instrument (needle) goes into your arm. On rare occasions, infection may occur at the site of blood draw or fingerstick. All efforts will be made to ensure this doesn't happen by using standard clean and sterile procedures for drawing blood. In the unlikely event that the infection occurs at the site of blood draw, trained clinicians will help you get the necessary treatment or refer you the appropriate health facility, at no cost to you.

You may become embarrassed, worried, or anxious when talking about your sexual practices, ways to protect against HIV and other infections passed during sex, and your test results. You may become worried or anxious while waiting for your test results. Having the screening tests, talking about HIV, and finding out your results could cause problems between you and your partner. The counseling that you will receive through the study staff will help you understand the results. Trained counselors are available through the study who will help you deal with any feelings or questions you may have.

The study staff will make every effort to protect your privacy and confidentiality while you are having the screening tests. However, it is possible that others may learn of your participation here and, because of this, may treat you unfairly or discriminate against you. For example, you could have problems getting or keeping a job, or being accepted by your family or community.

BENEFITS

You may get no direct benefit from the screening tests. However, you will get counseling and testing for HIV. You will get information on how to protect against HIV and other infections passed during sex. You will get free condoms. For other health problems that cannot be treated at this clinic, the study staff will tell you about other places you can go for treatment. The study staff will also tell you about other places that provide HIV prevention and care services. You may also contribute to understanding HIV prevention in women in Africa, which will help others in the future.

COSTS TO YOU

There is no cost to you for being in this study. Treatments available to you by the study will be free of charge.

REASONS WHY YOU MAY BE WITHDRAWN FROM THE STUDY
You may be removed from the study without your consent for the following reasons:
- The research study is stopped or canceled.
- You are not willing to find out your HIV test result.
- You are put in prison
- You are not able to attend clinic visits or complete the screening tests.
- The researchers may determine that this study may not be a good fit for you or may not be safe for you or your baby.
- The study staff feels that having the screening tests would be harmful to you.

ALTERNATIVES TO PARTICIPATION
There may be other studies going on here or in the community that you or your partner may be eligible for. You may also be able to access PrEP through other research studies going on here or in other clinics in the community. If you wish, the study staff will tell you about other studies or healthcare facilities which offer PrEP that we know about. There also may be other places you can go for HIV counseling and testing. We will tell you about those places if you wish.

REIMBURSEMENT
You will receive reimbursement for your transport for visiting the clinic, and 400 KSh for your time and effort at each scheduled study visit.

CONFIDENTIALITY
All study staff will make every effort to protect the privacy of your personal information but cannot completely guarantee this. Any sample from you or information about you will be identified only by code and not by name. The link between your name and code will be kept in a secure location at the clinic only. The U.S. Food and Drug Administration (FDA) reserves the right to review study data that may contain identifying information. Any publication of this study will not use your name or identify you personally. Certain people and organizations will need to see, copy, and use your health data so that they can do their part in the study. They are called 'authorized users'. The records of your tests may be reviewed by study staff and representatives of the following authorized users:
- Kenya Medical Research Institute (KEMRI) Scientific and Ethics Review Unit (SERU)
- Kenya Pharmacy and Poisons Board
- University of Washington, including study monitors
- US National Institutes of Health
- Kenya Ministry of Health
- University of Colorado
- Other local, U.S. and international regulatory entities

The review of records may be done on-site or remotely through secured authorized computer programs. Remote monitoring visits may be performed in place of, or in addition to onsite visits to ensure the safety of study participants and data integrity.

We have a Certificate of Confidentiality from the NIH. These protections only apply to data held in the United States. This helps us protect your privacy. The certificate means that we do not have to give out information, documents, or samples that could identify you even if we are asked to by

a court of law in the United States. We will use the Certificate to resist any demands for identifying information. We can't use the Certificate to withhold your research information if you give your written consent to give it to an insurer, employer, or other person. Also, you or a member of your family can share information about yourself or your part in this research if you wish. There are some limits to this protection. We will voluntarily provide the information to:

- a member of the United States government who needs it in order to audit or evaluate the research.
- individuals at the institution(s) conducting the research, the funding agency, and other groups involved in the research, if they need the information to make sure the research is being done correctly.
- individuals who want to conduct secondary research if allowed by federal regulations and according to your consent for future research use as described in this form.
- to relevant authorities as required by other Federal, State, or local laws.

The Certificate expires when the NIH funding for this study ends. Currently this is 31st January 2025. Any data collected after expiration is not protected as described above. Data collected prior to expiration will continue to be protected.

FUTURE USE OF DATA

The information that we obtain from you for this study might be used for future studies. We may remove anything that might identify you from the information. If we do so, that information may then be used for future research studies or given to another investigator without getting additional permission from you. It is also possible that in the future we may want to use or share study information that might identify you. If we do, the Scientific and Ethics Review Unit (SERU) and the researchers' appropriate ethical committee or institutional review board will decide whether or not we need to get additional permission from you.

RETURN OF RESEARCH RESULTS

You will be made aware of the results of rapid HIV, hepatitis B, creatinine, blood count, and urine pregnancy tests done as part of screening procedures for research study. If any results are abnormality and you will be referred to health facility where can get it the treatment or to a health facility of your choice.

RESEARCH-RELATED INJURY

We do not anticipate any research related injury. However, if you feel you are injured from participating in this study, you will be offered care at the study clinic, free of charge. For research related injury, please call the 24-hour emergency number: 0736464299.There is not a program of monetary compensation through this institution. The NIH also does not provide direct compensation for research related injuries. If you need medical care that we cannot provide, we will refer you to the appropriate services or organizations that can provide care for the injury. You do not give up any legal rights by signing this consent form. You do not waive any right to seek payment or give up any legal rights by signing this consent form.

PROBLEMS OR QUESTIONS
If you ever have any questions about this study, or if you have a research-related injury, you should contact Catherine Kiptinness at the Thika Partners in Prevention Study clinic at Tel. 067 2222561/0725 641110. For research related injury, please call the 24-hour emergency number: 0736464299. If you have questions about your rights as a research participant, you should contact: The Head, Scientific and Ethics Review Unit (SERU) Kenya Medical Research Institute (KEMRI), P.O Box 54840-00202 Nairobi Phone No. 0717719477, Email address: seru@kemri.org. A description of this study will also be available on https://www.clinicaltrials.gov as required by U.S. Law. This Web site will not include information that can identify you. At most, the Web site will include a summary of the results. You can search this Web site at any time.

STATEMENT OF CONSENT AND SIGNATURES
I have read the study enrollment consent for this study, or had it read to me. I have discussed the information with study staff. My questions have been answered. I understand that my decision whether or not to take part in the study is voluntary. I understand that if I decide to join the study I may withdraw at any time. By signing this form, I do not give up any rights that I have as a research participant.

____________________ Participant Name (print)	_________________________ Participant Signature/Thumbprint	______________ Date
____________________ Study Staff Name	________________________ Staff Signature	_____________ Date
____________________ Witness Name (print)	________________________ Witness Signature	______________ Date

<u>Participating in future research studies</u>
We would like to contact you in the future to see if you would be interested in participating in future studies. Please indicate below if you are willing to be contacted about any future research studies.

☐ Yes: I agree to be contacted about future research studies

☐ No: I do not want to be contacted about future research studies

____________________ Participant Name (print)	_________________________ Participant Signature/Thumbprint	______________ Date
____________________ Study Staff Name	________________________ Staff Signature	_____________ Date
____________________ Witness Name (print)	________________________ Witness Signature	______________ Date

Study Enrollment Informed Consent Form — Non-Pregnant Cisgender Women

Version 1.0, May 20, 2021

INVESTIGATORS

Investigator	Title	Institution	Telephone
Kenneth Mugwanya	MBChB, MS, PhD	University of Washington	001 206-520-3886
Nelly Mugo	MBChB, MMed, MPH	Kenya Medical Research Institute	067 2222561
Peter Anderson	PharmD	University of Colorado	001 303-724-6128
Elizabeth Irungu	MBChB, MPH	Kenya Medical Research Institute	067 2222561
Catherine Kiptinness	B. Pharm, MPH	Kenya Medical Research Institute	067 2222561

STUDY LOCATION: The study will be conducted at Thika.

You are being asked to take part in a research study. The box below tells you important things you should think about before deciding to join the study. We will provide more detailed information below the box. Please ask questions about any of the information before you decide whether to participate. You may also wish to talk to others (for example, your family, friends, or your doctor) about this study, before agreeing to join.

Key Information for You to Consider
• **Voluntary Consent**. You are being asked to volunteer for a research study. It is up to you whether you choose to participate or not. There are no penalties and you will not lose anything if you decide not to joint or if after you join, you decide to quit.<br>• **Purpose**. We are doing this research to find out more about the medication called Tenofovir/Emtricitabine (TDF/FTC), which can lower the chances for HIV-uninfected men and women to get the HIV virus. This is also called preexposure prophylaxis or PrEP. We want to learn more about the levels of PrEP medication in blood and other body parts when used by women.<br>• **Duration.** Your part of the study will last up to 4 months.<br>• **Procedures and Activities.** We will ask you questions about yourself including your medical and sexual history. At study specified visits you will take a tablet of PrEP in the presence of study staff and study specified samples including hair, blood, tissue and fluids from your genital area will be collected.<br>• **Risks.** Most studies have some possible harms that could happen to you if you join. In this study, we expect that the primary risks will be some pain and discomfort from sample collection, stress or discomfort from answering survey questions, and breach in confidentiality<br>• **Benefits**. We expect some benefits from this study, as well. For you, we don't think that you may get direct benefit from being in this study. However, we will inform you about any test results that are done that could be useful to your health care.<br>• **Alternatives.** Participation is voluntary and the only alternative is to not participate.

INFORMED CONSENT

We are asking you to volunteer for a research study about medication used to prevent HIV. This study is open to women who do not have HIV and are not pregnant. Before you decide whether to take part in the study, we would like to explain the purpose of the study, the risks and benefits,

and what would be expected of you if you agree to be in the study. This study is being coordinated through the Kenya Medical Research Institute in Kenya and the University of Washington located in Seattle, Washington, USA. The study is being funded through the US National Institutes of Health in Washington, DC, USA. If you decide to participate in the research study, you will be asked to sign this consent form or make your mark in front of a witness. We will give you a copy of this form if you choose to have. This consent form might contain some words that are unfamiliar to you. Please ask us to explain anything you may not understand.

PURPOSE OF THE STUDY

A medication called Tenofovir/emtricitabine (TDF/FTC) can lower the chances for HIV-uninfected men and women to get the HIV virus. This is called preexposure prophylaxis or PrEP. PrEP medication is safe to use, does not lead to serious problems during pregnancy and babies of mothers who use PrEP do not have serious birth problems. PrEP is approved by the World Health Organization and the Kenya Ministry of Health as one option to prevent getting HIV. Research studies have also shown that PrEP works only when taken as prescribed. Other research studies have shown that the levels of drugs in blood after taking PrEP may be different in men and women, especially during pregnancy. The purpose of this study is to find out more about the levels of PrEP in blood and other body part samples when used by women. Overall, approximately 72 women, all from Kenya, will be in the study. The samples and data that we collect in this study may help us learn more how these medication work to prevent HIV infection in women**.** All participants who agree to be in this study will be asked to provide data and samples for up to 16 weeks.

YOUR PARTICIPATION IS VOLUNTARY

This consent form gives information about the study that we will discuss with you. Before you learn about the study procedures, it is important that you know the following:

- You do not have to be in this study if you do not want to join.
- You may decide not to take part in the study, or to withdraw from the study at any time.
- If you decide not to take part in the study, you can still join another research study later, if one is available and you qualify.

STUDY PROCEDURES

If you decide to join the study, you will have study visits for up to 16 weeks. At these visits, the study staff will confirm where you live and other questions about your sexual practices, medical history and, pregnancy and birth control use intentions. A clinician will also review your medication and vaccine history. If you are eligible to participate in the study, then the study team will randomly assign you by chance (like flipping a coin) to one of three study groups. The staff do not know ahead of time which group you will fall into.

At each clinic visit, the study staff will check your temperature and ask you questions about COVID-19 symptoms and people you been in physical contact with, to see if you have been exposed to COVID-19. Should the staff determine that you have been exposed, they will follow the recommended Ministry of Health guidelines in referring you for healthcare. We will reschedule your visit for another time based on these guidelines.

At study specified visits you will take a tablet of PrEP in the presence of study staff and study specified samples will be collected.

At enrollment you:

- Will be told the number of visits and numbers of doses of PrEP you will take every week. There are three groups of dosing of PrEP you may be assigned to. You may either be in a group that will take **two doses every week** or **four doses in a week** or **seven doses every week**. Only chance will determine which group you will be in.
- If you are assigned to **two (2) doses every week**, you will come to the clinic to take your medication on **<u>Mondays and Tuesdays</u>**.
- If you are assigned to **four (4) doses every week**, you will be asked to come to the clinic to take your medication on **<u>Mondays, Tuesdays, Thursdays, and Fridays</u>** of every week.
- If you are assigned to **seven (7) doses every week**, you will come to the clinic **<u>every day of the week Monday to Sunday</u>** to take your medication.
- Will be given a study follow-up calendar to help you remember study visits.
- Will be provided with a month supply (a bottle) of back-up medication to be used if you are unable to complete the visit in person at the clinic.

At enrollment and each study visit you:

- Will be given PrEP tablet medication to take in their presence (called directly observed therapy) at the clinic. All doses taken away from the clinic will still be directly observed by study staff via live video streaming on a smart cell phone or computer via WhatsApp. For doses taken via video streaming, you will be asked to open your mouth afterwards to confirm swallowing. You will be supported with reasonable internet bundles consistent with the SERU recommendation.
- Will be counseled about possible drug side effects.
- Will be asked questions about your health and medical history, sexual behaviors, and your feelings about taking medication for HIV prevention.
- Will talk with study staff about ways to avoid HIV and other infections passed during sex. You will be offered condoms.
- Will get medical care or referrals for medical care and other services if you need them.
- Will be asked to give updated information on where you live and how to keep in contact with you. The study staff will use this information to remind you of scheduled visits. If you don't return to the clinic on schedule, the study staff may try to contact you by phone or by visiting your home. They may try to reach you through the contact people that you list. If they talk to these people, they will not tell them why they are trying to reach you. If a home visit is done, study staff may ask you take study drug in their presence or provide some scheduled study samples.
- Obtain blood samples [up to 21 mL / about one and quarter tablespoon] at each of these visits.
- Provide a urine sample in a container [up to 10 mL / a little more than half tablespoon] at each of these visits

At enrollment, day 4, week 1, week 2, week 4, week 6, and week 8 visits:
We will ask your permission to obtain study samples at these visits. Study samples may include blood, urine, hair, and vaginal tissue. The samples will be used by study researchers for tests to determine the amount of study drugs in the samples. We will ask for your permission to:

- Obtain hair samples at week 4 and week 8. The hair sample will be about 50-100 strands (which is what we need for this test) and is less than you naturally lose from your head each day. We will test for PrEP drug levels in the hair sample at the end of the study. Your hair will not be stored in any way but destroyed after testing for the PrEP drug levels

- Obtain vaginal swab and biopsy at day 4, week 1, week 4 and week 8. Women who provide vaginal biopsy at day 4 will not have another biopsy at week 1 but will provide the remaining samples at week 4 and week 8. **When you provide a vaginal biopsy, you must abstain from sex for at least seven days.**
- Obtain a blood sample by finger prick [**week 1 (days 4 and 7), week 4 and week 8**].
- Provide urine for a pregnancy test at entry, week 4 and week 8 visits. If you become pregnant, you will stop taking the study drug and we will continue to follow you till the end of your pregnancy.
- Conduct and HIV-1 rapid test at week 4 and week 8 visits.
- If you become infected with HIV, you will stop taking the study drug and we will then refer you to a nearby HIV care services or at a clinic of your choice for follow up at completion of this trial.
- If you withdraw or are withdrawn before the end of the study, we may ask you to complete a separate early exit visit, so that we can check you, to make sure you are healthy before your exit.

After 8 weeks of taking PrEP medication:
We will ask you to come in for two additional visits, where we will ask for your permission to obtain the following study samples:

- Blood samples [up to 21 mL / about one and quarter tablespoon] at each of these visits.
- Urine sample in a container [up to 10 mL / a little more than half tablespoon] at each of these visits
- Obtain hair samples at your final visit. The hair sample will be about 50-100 strands (which is what we need for this test) and is less than you naturally lose from your head each day. We will test for PrEP drug levels in the hair sample at the end of the study. Your hair will not be stored in any way but destroyed after testing for the PrEP drug levels

BIOMETRIC FINGERPRINT SCAN
To help us keep track of who is enrolled in our clinic, and who is coming for follow up visits, we will ask you to put either your right or left index finger on a small machine that will scan your fingerprint. This scan will be linked to a unique identification number and will be accessible only to study staff. We will take your fingerprint at each study visit. You are free to refuse to have your fingerprint taken and this will not affect your participation in the study. The fingerprint database will be destroyed after completion of active follow up in the study.

SPECIMEN STORAGE AND USE OF SAMPLES AND DATA FOR FUTURE STUDIES. We would like to save data from this study and samples of your blood, urine, tissue, and hair at the Thika clinic and the University of Washington for future research by us and by other researchers. Some samples will be shipped to our laboratories in Mombasa and Nairobi, Kenya for testing. For specialized tests that cannot be done in Kenya, your samples will be shipped to the University of Washington and University of Colorado in the USA. **You will not receive any results that come from samples that are tested in the USA.** We will use these samples only for research related to HIV, sexually transmitted infections and reproductive health. This will include studies on HIV and STI prevention, how people respond to the medications used in this study or how the drugs

interact with contraception, organism in genital areas or studies of variation in gene DNA of drug transporter. Gene studies will only be for studies related to proteins which transport drugs in the body (drug transporters) not for full genome wide sequencing.
This research is experimental, and these tests are not useful for your clinical care. Before your samples leave the clinic, they will be assigned a code and your name will not be on them. Your name will be linked to the code only at this clinic. If you do not want to have your samples saved for future research, you can still be in this study and your samples will be destroyed once testing for this study is completed. If you agree to store your samples now, but change your mind before the study ends, let the study staff know and we will make sure your samples do not get stored for future research. There will be no consequences for changing your mind. However, after the link between your samples and your name is destroyed, we will not be able to identify your samples. We will not sell your data or samples. Tests done on your samples may lead to a new invention or discovery. We have no plans to share money or other benefits resulting from such invention or discovery with you.

FUTURE USE OF DATA AND SAMPLES

The information and/or specimens that we obtain from you for this study might be used for future studies. We may remove anything that might identify you from the information and specimens. If we do so, that information and specimens may then be used for future research studies or given to another investigator without getting additional permission from you. It is also possible that in the future we may want to use or share study information that might identify you. If we do, SERU and the researchers' appropriate ethical committee or institutional review board will decide whether or not we need to get additional permission from you.

RETURN OF RESEARCH RESULTS

You will be made aware of the results of rapid HIV, hepatitis B, creatinine, blood count, and urine pregnancy tests done as part of research. Trained research staff will share the results with you in person during visits or through a phone call. You will also be made aware of results from batched baseline STI testing. If any results are abnormal, you will receive standard of care treatment here at the clinic in a timely manner. For treatment not available at the clinic, you will be referred to clinics where you can get it. Testing on blood drug levels or future studies will not be shared with you. This research is experimental, and these tests are not useful for your clinical care.

RISKS

You may become embarrassed, worried, or anxious when talking about your sexual practices, ways to protect against HIV and other infections passed during sex. If you are not now infected with HIV but become infected with HIV, this could make you worried or anxious. Talking about HIV and finding out your test results could cause problems between you and your partner. Trained counselors will help you and your partner deal with any feelings or questions you may have about these issues. If your partner finds out that you are on PrEP, this may lead to disagreements or physical/verbal abuse. It may also lead to economic risks such as loss of income or financial support. We anticipate that these risks will be rare. If threats of violence arise, avoid confrontation and consider seeking help from family, friends, your other social networks or study staff.

The study staff will make every effort to protect your privacy and confidentiality while you are

having the study procedures. However, it is possible that others may learn of your participation here and, because of this, may treat you unfairly or discriminate against you. For example, you could have problems getting or keeping a job, or being accepted by your family or community.
Risks related to collection of study samples: This study requires the use of your blood. There will be some pain associated with the needle stick, but this will be only for a short period of time. There may be some bruising around the needle site and, although we will sterilize the site to minimize infection, there is a very minimal risk of infection at the site. You may feel discomfort or pain when your blood is drawn. You may feel dizzy or faint. You may also have slight discomfort or pain during the prick of your finger, and there is a small risk of infection, bleeding or bruising from the fingerstick.

Some people may be embarrassed about providing urine, hair, and tissue samples. There is also some discomfort associated with taking hair or biopsy specimens. The biopsy may feel like a pinch each time a sample is taken and may cause some cramping with it. Any pain or cramping occurring during the biopsy may be helped by relaxing and taking a few slow deep breaths. Some vaginal bleeding and a small amount of dark brown discharge are normal after the sampling. Abstaining from sex for at least 7 days after the biopsy, and taking the HIV-prevention drug, TDF/FTC, will help reduce your risk of HIV infection after having biopsies collected. **If you become pregnant during the study, you will not have a vaginal biopsy**. We will still perform vaginal swab, secretion collection, which are not a risk for the pregnancy.

Risks potentially related to the TDF/FTC PrEP medication: You may have symptoms or adverse effects while participating in the study. The adverse effects that can occur in a small proportion of people taking PrEP are well known because the medication has been used by many people. PrEP medication is very safe but like any medication, some mild adverse effects are expected to occur in up to 1 in 10 persons who take PrEP. These include headache, stomach upset, vomiting or loss of appetite. Occasional adverse effects may include mild problems of kidney function that are only detected by laboratory tests. Small changes in the mineral density of bones were observed in studies of people who were given PrEP, but the changes in the mineral density of the bones did not cause any fractures, or other problems that bothered the patients.
When used together with other drugs for treatment, in some people with advanced HIV infection, symptoms from other infections or certain diseases may occur soon after starting combination anti-HIV treatment but can also occur later. The use of potent antiretroviral drug combinations may be associated with an abnormal placement of body fat and wasting. Some of the body changes include:

- Increase in fat around the waist and stomach area
- Increase in fat on the back of the neck
- Thinning of the face, legs and arms
- Breast enlargement
- Gas, loose or watery stools
- Generalized weakness
- Dizziness
- Depression
- Rash
- Allergic reaction: symptoms may include fever, rash, upset stomach, vomiting, loose or

watery stools, abdominal pain, achiness, shortness of breath, a general feeling of illness or a potentially serious swelling of the face, lips, and/or tongue
- Muscle pain and muscle weakness
- Sleeping problems

Lactic acidosis has occurred in some HIV-infected persons taking PrEP, in combination with other drugs. Lactic acidosis is a condition that can produce shortness of breath, nausea, and liver failure. This is a serious adverse effect of some medications used for HIV infection. Other potential serious but rare risks include kidney failures and potential injuries your kidneys such as Fanconi's syndrome (rental tubular injury with severe hypophosphatemia), and hypersensitivity reactions. **If you have these symptoms, or any other symptoms that concern you, the study staff will evaluate your symptoms and determine whether you should stop your PrEP pills.** You will be given a telephone number where the study doctors will be available 24 hours a day, 7 days a week.

Risk of acquiring HIV infection: Only daily PrEP is recommended for use to prevent HIV in women in Kenya. In this study you may receive non-daily dosing of PrEP which doesn't provide sufficient protection. You may be exposed to get HIV from sexual partners you may have. It is very important to use all the known strategies to prevent getting HIV, like using condoms for all sexual relations and keeping your number of sexual partners low. Each visit you will getting risk-reduction counseling for ways to protect. You will have an HIV test every 4 weeks. If we find that you have HIV, PrEP will be immediately discontinued. You will be started on free HIV treatment immediately as per the Kenya national guidelines and we will then refer you to a nearby HIV care services or at a clinic of your choice for follow up at completion of this trial.

New findings

We will provide you with any new information that we learn during the study, which might affect your willingness to participate.

BENEFITS

You may get no direct benefit from being in this study. We will inform you about any test results that are done that could be useful your health care. You or others may benefit in the future from information learned in this study. You also may get some personal satisfaction from being part of research on HIV. While enrolled in the study you will get all services available to those not in the study such as access to oral PrEP (which is known to help prevent HIV infection), counseling for reducing your risk of giving HIV to or getting HIV from your sexual partner, and free condoms. For care and treatment that is not available at the clinic, study staff will tell you about other places where care and treatment may be available. Your participation may also contribute to understanding about HIV prevention in women in Africa, which will help others in the future.

COSTS TO YOU

There is no cost to you for being in this study. Treatments available to you by the study will be free of charge.

REASONS WHY YOU MAY BE WITHDRAWN FROM THE STUDY

You may be removed from the study without your consent for the following reasons:

- The study is stopped or canceled.

- You are put in prison.
- Your intentions about pregnancy and birth control use change.
- The study staff feel that staying in the study would be harmful to you.
- You are not able to attend study visits or complete the study procedures.

ALTERNATIVES TO PARTICIPATION

There may be other studies going on here or in the community that you or your partner may be eligible for. You may also be able to access PrEP through other research studies going on here or in other clinics in the community. If you wish, the study staff will tell you about other studies or healthcare facilities which offer PrEP that we know about. There also may be other places you can go for HIV counseling and testing. We will tell you about those places if you wish.

REIMBURSEMENT

You will receive reimbursement for your transport for visiting the clinic, and 400 KSh for your time and effort at each scheduled study visit. All visits that include collection of biopsies will be reimbursed 2000 KSh. All visits that include collection of vaginal swabs alone will be reimbursed 1000 KSh.

CONFIDENTIALITY

Government or university staff sometimes review studies such as this one to make sure they are being done safely and legally. If a review of this study takes place, your records may be examined. The reviewers will protect your privacy. The study records will not be used to put you at risk of legal harm. All study staff will make every effort to protect the privacy of your personal information but cannot completely guarantee this. Any sample from you or information about you will be identified only by code and not by name. The link between your name and code will be kept in a secure location at the clinic only. The U.S. Food and Drug Administration (FDA) reserves the right to review study data that may contain identifying information. Any publication of this study will not use your name or identify you personally. Certain people and organizations will need to see, copy, and use your health data so that they can do their part in the study. They are called 'authorized users'. The records of your tests may be reviewed by study staff and representatives of the following authorized users:

- Kenya Medical Research Institute (KEMRI) Scientific and Ethics Review Unit (SERU)
- Kenya Pharmacy and Poisons Board
- University of Washington, including study monitors
- US National Institutes of Health
- Kenya Ministry of Health
- University of Colorado
- Other local, U.S. and international regulatory entities

The review of records may be done on-site or remotely through secured authorized computer programs. Remote monitoring visits may be performed in place of, or in addition to onsite visits to ensure the safety of study participants and data integrity.

We have a Certificate of Confidentiality from the NIH. These protections only apply to data held in the United States. This helps us protect your privacy. The certificate means that we do not have to give out information, documents, or samples that could identify you even if we are asked to by

a court of law in the United States. We will use the Certificate to resist any demands for identifying information. We can't use the Certificate to withhold your research information if you give your written consent to give it to an insurer, employer, or other person. Also, you or a member of your family can share information about yourself or your part in this research if you wish. There are some limits to this protection. We will voluntarily provide the information to:

- a member of the United States government who needs it in order to audit or evaluate the research.
- individuals at the institution(s) conducting the research, the funding agency, and other groups involved in the research, if they need the information to make sure the research is being done correctly.
- individuals who want to conduct secondary research if allowed by federal regulations and according to your consent for future research use as described in this form.
- to relevant authorities as required by other Federal, State, or local laws.

The Certificate expires when the NIH funding for this study ends. Currently this is 31st January 2025. Any data collected after expiration is not protected as described above. Data collected prior to expiration will continue to be protected.

RESEARCH-RELATED INJURY

We do not anticipate any research related injury. However, if you feel you are injured from participating in this study, you will be offered care at the study clinic, free of charge. For research related injury, please call the 24-hour emergency number: 0736464299.There is not a program of monetary compensation through this institution. The NIH also does not provide direct compensation for research related injuries. If you need medical care that we cannot provide, we will refer you to the appropriate services or organizations that can provide care for the injury. You do not give up any legal rights by signing this consent form. You do not waive any right to seek payment or give up any legal rights by signing this consent form.

PROBLEMS OR QUESTIONS

If you ever have any questions about this study, or if you have a research-related injury, you should contact Catherine Kiptiness at the Thika Partners in Prevention Study clinic at Tel. 067 2222561/0725 641110. For research related injury, please call the 24-hour emergency number: 0736464299. If you have questions about your rights as a research participant, you should contact: The Head, Scientific and Ethics Review Unit (SERU) Kenya Medical Research Institute (KEMRI), P.O Box 54840-00202 Nairobi Phone No. 0717719477, Email address: seru@kemri.org. A description of this study will also be available on https://www.clinicaltrials.gov. as required by U.S. Law. This Web site will not include information that can identify you. At most, the Web site will include a summary of the results. You can search this Web site at any time.

STATEMENT OF CONSENT AND SIGNATURES

I have read the study enrollment consent for this study, or had it read to me. I have discussed the information with study staff. My questions have been answered. I understand that my decision whether or not to take part in the study is voluntary. I understand that if I decide to join the study I may withdraw at any time. If I refuse to be in the study or decide to stop being in the study I will have no loss of benefits to which I am already entitled. By signing this form, I do not give up any rights that I have as a research participant.

Participant Name (print)	Participant Signature/Thumbprint	Date
Study Staff Name (print)	Study Staff Signature	Date
Witness Name (print)	Witness Signature	Date

SPECIMEN STORAGE AND USE OF YOUR DATA AND SAMPLES FOR FUTURE STUDIES:
Please initial and date one option:

___________ I agree for my specimen/information to be used for future research and shared with other researchers without my additional consent without identifiers

___________ I DO NOT agree for my specimen/information to be used for future research or shared with other researchers with or without identifiers

STATEMENT OF CONSENT: BIOMETRIC FINGERPRINT
Please initial and date one option:

___________ I agree to have my fingerprint taken

____________I DO NOT agree to have my fingerprint taken

Participant Name (print)	Participant Signature/Thumbprint	Date
Study Staff Name (print)	Study Staff Signature	Date
Witness Name (print)	Witness Signature	Date

Copies to;
1. Investigators
2. Study participant

Enrollment Informed Consent Form — Pregnant Cisgender Women

Version 1.0, May 20, 2021

INVESTIGATORS

Investigator	Title	Institution	Telephone
Kenneth Mugwanya	MBChB, MS, PhD	University of Washington	001 206-520-3886
Nelly Mugo	MBChB, MMed, MPH	Kenya Medical Research Institute	067 2222561
Peter Anderson	PharmD	University of Colorado	001 303-724-6128
Elizabeth Irungu	MBChB, MPH	Kenya Medical Research Institute	067 2222561
Catherine Kiptinness	B. Pharm, MPH	Kenya Medical Research Institute	067 2222561

STUDY LOCATION: The study will be conducted at Thika.

You are being asked to take part in a research study. The box below tells you important things you should think about before deciding to join the study. We will provide more detailed information below the box. Please ask questions about any of the information before you decide whether to participate. You may also wish to talk to others (for example, your family, friends, or your doctor) about this study, before agreeing to join.

Key Information for You to Consider

- **Voluntary Consent**. You are being asked to volunteer for a research study. It is up to you whether you choose to participate or not. There are no penalties and you will not lose anything if you decide not to joint or if after you join, you decide to quit.
- **Purpose**. We are doing this research to find out more about the medication called Tenofovir/Emtricitabine (TDF/FTC), which can lower the chances for HIV-uninfected men and women to get the HIV virus. This is also called preexposure prophylaxis or PrEP. We want to learn more about the levels of PrEP medications in blood and other body parts when used by women.
- **Duration.** Your part of the study will last up to 4 months.
- **Procedures and Activities.** We will ask you questions about yourself including your medical and sexual history. At study specified visits you will take a tablet of PrEP in the presence of study staff and study specified samples including hair, blood and, flesh and fluids from your genital area will be collected.
- **Risks.** Most studies have some possible harms that could happen to you if you join. In this study, we expect that the primary risks will be some pain and discomfort from sample collection, stress or discomfort from answering survey questions, and breach in confidentiality
- **Benefits**. We expect some benefits from this study, as well. For you, we don’t think that you may get direct benefit from being in this study. However, we will inform you about any test results that are done that could be useful to your health care.
- **Alternatives.** Participation is voluntary and the only alternative is to not participate.

INFORMED CONSENT
We are asking you to volunteer for a research study about medication used to prevent HIV. This study is open to women who do not have HIV and are pregnant. Before you decide whether to take part in the study, we would like to explain the purpose of the study, the risks and benefits, and what would be expected of you if you agree to be in the study. This study is being coordinated through the Kenya Medical Research Institute (KEMRI) in Kenya and the University of Washington located in Seattle, Washington, USA. The study is being funded through the US National Institutes of Health in Washington, DC, USA. If you decide to participate in the research study, you will be asked to sign this consent form or make your mark in front of a witness. We will give you a copy of this form if you choose to have. This consent form might contain some words that are unfamiliar to you. Please ask us to explain anything you may not understand.

PURPOSE OF THE STUDY
A medication called Tenofovir/Emtricitabine (also called TDF/FTC) can lower the chances for HIV-uninfected men and women to get the HIV virus. This is called preexposure prophylaxis or PrEP. PrEP medication is safe to use and does not lead to serious problems during pregnancy and there is no current evidence that PrEP harms babies of women who use it during pregnancy.. PrEP is approved by the World Health Organization and the Kenya ministry of health as one option to prevent getting HIV. Research studies have also shown that PrEP works only when taken as prescribed. Other research studies have shown that the levels of drugs in blood after taking PrEP may be different in men and women, especially during pregnancy. The purpose of this study is to find out more about the levels of PrEP in blood and other body samples when used by women. Overall, approximately 72 women, all from Kenya, will be in the study. The samples and data that we collect in this study may help us learn more how these medication work to prevent HIV infection in women**.** All participants who agree to be in this study will be asked to provide data and samples for up to 16 weeks.

YOUR PARTICIPATION IS VOLUNTARY
This consent form gives information about the study that we will discuss with you. Before you learn about the study procedures, it is important that you know the following:

- You do not have to be in this study if you do not want to join.
- You may decide not to take part in the study, or to withdraw from the study at any time.
- If you decide not to take part in the study, you can still join another research study later, if one is available and you qualify.

STUDY PROCEDURES
If you decide to join the study, you will have study visits up to 16 weeks. At these visits, the study staff will confirm where you live and other questions about your sexual practices, medical history and review your medication and vaccine history. At each visit, we will also review your antenatal care records and check the heartbeat of your unborn baby. Every day you will take a tablet of PrEP in the presence of study staff and study samples will be collected on specified visit days. If you withdraw or are withdrawn before the end of the study, we may ask you to complete a separate early exit visit, so that we can check you, to make sure you are healthy before your exit.

At each clinic visit, the study staff will check your temperature and ask you questions about COVID-19 symptoms and people you been in physical contact with, to see if you have been

exposed to COVID-19. Should the staff determine that you have been exposed, they will follow the recommended Ministry of Health guidelines in referring you for healthcare. We will reschedule your visit for another time based on these guidelines.

At enrollment you:

- Will be instructed to **come to clinic every day of the week Monday to Sunday to take your medication.**
- Will be given a study follow-up calendar to help you remember study visits.
- Will be provided with a month supply (a bottle) of back-up medication to be used if you are unable to complete the visit in person at the clinic.

At enrollment and each study visit you:

- Provide blood samples [up to 21 mL / about one and quarter tablespoon] at each of these visits.
- Provide a urine sample in a container [up to 10 mL / a little more than half tablespoon] at each of these visits
- Will be given PrEP tablet medication to take in their presence (called directly observed therapy) at the clinic. All doses taken away from the clinic will still be directly observed by study staff via live video streaming on a smart cell phone or computer via WhatsApp. For doses taken via video streaming, you will be asked to open your mouth afterwards to confirm swallowing. You will be supported with reasonable internet bundles consistent with the SERU recommendation.
- Will be counseled about possible drug side effects.
- Will be asked questions about your health and medical history, sexual behaviors, and your feelings about taking medication for HIV prevention.
- Will talk with study staff about ways to avoid HIV and other infections passed during sex. You will be offered condoms.
- Will get medical care or referrals for medical care and other services if you need them.
- Will be asked to give updated information on where you live and how to keep in contact with you. The study staff will use this information to remind you of scheduled visits. If you don't return to the clinic on schedule, the study staff may try to contact you by phone or by visiting your home. They may try to reach you through the contact people that you list. If they talk to these people, they will not tell them why they are trying to reach you. If a home visit is done, study staff may ask you take study drug in their presence or provide some scheduled study samples.

At enrollment, day 4, week 1, week 2, week 4, week 6, and week 8 visits:
We will ask your permission to obtain study samples at these visits. Study samples may include blood, urine, hair, and vaginal tissue. The samples will be used by study researchers for tests to determine the amount of study drugs in the samples. We will ask for your permission to:

- Obtain hair samples at week 4 and week 8. The hair sample will be about 50-100 strands (which is what we need for this test) and is less than you naturally lose from your head each day. We will test for PrEP drug levels in the hair sample at the end of the study. Your hair will not be stored in any way but destroyed after testing for the PrEP drug levels
- Obtain a blood sample by finger prick [**week 1 (days 4 and 7), week 4 and week 8**].
- Conduct an HIV-1 rapid test at week 4 and week 8 visits.
- Obtain a vaginal swab [**at enrollment, day 4, week 1, week 4 and week 8**].

After 8 weeks of taking PrEP medication:
We will ask you to come in for two additional visits, where we will ask for your permission to obtain the following study samples:

- Blood samples [up to 21 mL / about one and quarter tablespoon] at each of these visits.
- Urine sample in a container [up to 10 mL / a little more than half tablespoon] at each of these visits
- Obtain hair samples at your final visit. The hair sample will be about 50-100 strands (which is what we need for this test) and is less than you naturally lose from your head each day. We will test for PrEP drug levels in the hair sample at the end of the study. Your hair will not be stored in any way but destroyed after testing for the PrEP drug levels

During pregnancy and after your baby is born:
We will contact you at the end of your pregnancy to ask about your health and the health of your baby. The study staff will continue to ask about your baby's health until their first birthday. We will collect information such as your baby's sex, weight, head size and height. With your permission, we will review your baby's medical records to collect this information or record them during your scheduled visits. We may also collect the information about your baby either through a phone call or during a scheduled visit. The scheduled visits for you and your baby after delivery, will be at 1 month, 6 months, and 12 months after the birth of your baby.

BIOMETRIC FINGERPRINT SCAN
To help us keep track of who is enrolled in our clinic, and who is coming for follow up visits, we will ask you to put either your right or left index finger on a small machine that will scan your fingerprint. This scan will be linked to a unique identification number and will be accessible only to study staff. We will take your fingerprint at each study visit. You are free to refuse to have your fingerprint taken and this will not affect your participation in the study. The fingerprint database will be destroyed after completion of active follow up in the study.

SPECIMEN STORAGE AND USE OF SAMPLES FOR FUTURE STUDIES
We would like to save data from this study and samples of your blood, urine, vaginal swab, and hair at the Thika clinic and the University of Washington for future research by us and by other researchers. Some samples will be shipped to our laboratories in Mombasa and Nairobi, Kenya for testing. For specialized tests that cannot be done in Kenya, your samples will be shipped to the University of Washington and University of Colorado in the USA. **You will not receive any results that come from samples that are tested in the USA.** We will use these samples only for research related to HIV and sexually-transmitted infections. This will include studies on HIV and STI prevention, how people respond to the medications used in this study or how the drugs interact with contraception, organism in genital areas or studies of variation in gene DNA of drug transporter. Gene studies will only be for studies related to proteins which transport drugs in the body (drug transporters) not for full genome wide sequencing.

This research is experimental and these tests are not useful for your clinical care. Before your samples leave the clinic, they will be assigned a code and your name will not be on them. Your name will be linked to the code only at this clinic. If you do not want to have your samples saved for future research, you can still be in this study and your samples will be destroyed once testing for this study is completed. If you agree to store your samples now, but change your mind before

the study ends, let the study staff know and we will make sure your samples do not get stored for future research. There will be no consequences for changing your mind. However, after the link between your samples and your name is destroyed, we will not be able to identify your samples. We will not sell your data or samples. Tests done on your samples may lead to a new invention or discovery. We have no plans to share any other benefits resulting from such invention or discovery with you.

FUTURE USE OF DATA AND SAMPLES
The information and/or specimens that we obtain from you for this study might be used for future studies. We may remove anything that might identify you from the information and specimens. If we do so, that information and specimens may then be used for future research studies or given to another investigator without getting additional permission from you. It is also possible that in the future we may want to use or share study information that might identify you. If we do, SERU and the researchers' appropriate ethical committee or institutional review board will decide whether or not we need to get additional permission from you.

RETURN OF RESEARCH RESULTS
You will be made aware of the results of rapid HIV, hepatitis B, creatinine, and blood count done as part of research. Trained research staff will share the results with you in person during visits or through a phone call. You will also be made aware of results from batched baseline STI testing. If any results are abnormal, you will receive standard of care treatment here at the clinic in a timely manner. For treatment not available at the clinic, you will be referred to clinics where you can get it. Testing on blood drug levels or future studies will not be shared with you. This research is experimental, and these tests are not useful for your clinical care.

RISKS
You may become embarrassed, worried, or anxious when talking about your sexual practices, ways to protect against HIV and other infections passed during sex. If you are not now infected with HIV but become infected with HIV, this could make you worried or anxious. Talking about HIV and finding out your test results could cause problems between you and your partner. Trained counselors will help you and your partner deal with any feelings or questions you may have about these issues. If your partner finds out that you are on PrEP, this may lead to disagreements or physical/verbal abuse. It may also lead to economic risks such as loss of income or financial support. We anticipate that these risks will be rare. If threats of violence arise, avoid confrontation and consider seeking help from family, friends, your other social networks or study staff.

The study staff will make every effort to protect your privacy and confidentiality while you are having the study procedures. However, it is possible that others may learn of your participation here and, because of this, may treat you unfairly or discriminate against you. For example, you could have problems getting or keeping a job, or being accepted by your family or community.

Risks related to collection of study samples: This study requires the use of your blood. There will be some pain associated with the needle stick, but this will be only for a short period of time. There may be some bruising around the needle site and, although we will sterilize the site to minimize infection, there is a very minimal risk of infection at the site. You may feel discomfort or

pain when your blood is drawn. You may feel dizzy or faint. You may also have slight discomfort or pain during the prick of your finger, and there is a small risk of infection, bleeding or bruising from the fingerstick. Some people may be embarrassed about providing urine and hair samples. There is also some discomfort associated with taking hair or vaginal swab specimens. Vaginal swab and secretion collection are collected using a piece of cotton which does not pose risks to the pregnancy.

Risks potentially related to the TDF/FTC PrEP medication: You may have symptoms or adverse effects while participating in the study. The adverse effects that can occur in a small proportion of people taking PrEP are well known because the medication has been used by many people. PrEP medication is very safe but like any medication, some mild adverse effects are expected to occur in up to 1 in 10 persons who take PrEP. These include headache, stomach upset, vomiting or loss of appetite. Occasional adverse effects may include mild problems of kidney function that are only detected by laboratory tests. Small changes in the mineral density of bones were observed in studies of people who were given PrEP, but the changes in the mineral density of the bones did not cause any fractures, or other problems that bothered the patients.
When used together with other drugs for treatment, in some people with advanced HIV infection, symptoms from other infections or certain diseases may occur soon after starting combination anti-HIV treatment but can also occur later. The use of potent antiretroviral drug combinations may be associated with an abnormal placement of body fat and wasting. Some of the body changes include:

- Increase in fat around the waist and stomach area
- Increase in fat on the back of the neck
- Thinning of the face, legs and arms
- Breast enlargement
- Gas, loose or watery stools
- Generalized weakness
- Dizziness
- Depression
- Rash
- Allergic reaction: symptoms may include fever, rash, upset stomach, vomiting, loose or watery stools, abdominal pain, achiness, shortness of breath, a general feeling of illness or a potentially serious swelling of the face, lips, and/or tongue
- Muscle pain and muscle weakness
- Sleeping problems

Lactic acidosis has occurred in some HIV-infected persons taking PrEP, in combination with other drugs. Lactic acidosis is a condition that can produce shortness of breath, nausea, and liver failure. This is a serious adverse effect of some medications used for HIV infection. Other potential serious but rare risks include kidney failures and potential injuries to your kidneys such as Fanconi's syndrome (rental tubular injury with severe hypophosphatemia), and hypersensitivity reactions.

Pregnancy is associated with gastro-intestinal symptoms (e.g., nausea, vomiting) which are similar to PrEP-related symptoms and therefore use of PrEP in pregnancy could potentially exacerbate these symptoms. **If you have these symptoms, or any other symptoms that**

concern you, the study staff will evaluate your symptoms and determine whether you should stop your PrEP pills. You will be given a telephone number where the study doctors will be available 24 hours a day, 7 days a week.

Risks to infants associated with exposure to TDF/FTC through breastfeeding are unknown but are likely to be very minimal. A study of TDF/FTC use in breastfeeding mothers and their infants in Uganda and Kenya showed that very low amounts of tenofovir medications are transferred into breast milk and no quantities were observed in the blood of infants breastfed by women using TDF/FTC for HIV prevention. Other studies in women during pregnancy and after birth also suggest that the frequency of adverse effects may not be different between babies exposed to TDF/FTC during pregnancy or breastfeeding compared to babies not exposed. While the evidence on safety is generally reassuring, there is need to get additional information on pregnancy outcomes and infant growth among those exposed to TDF/FTC PrEP.

Risk of acquiring HIV infection: Only daily PrEP is recommended for use to prevent HIV in women in Kenya. In this study, you will receive daily dosing of PrEP which provides enough protection against HIV if you take it well as prescribed. Although it is very unlikely to happen if you use PrEP as prescribed, there some small chance of getting HIV from sexual partners you may have. It is very important to use all the known strategies to prevent getting HIV, like using condoms for all sexual relations and keeping your number of sexual partners low. In addition to the PrEP, each visit you will receive condoms if you want them and get risk-reduction counseling on ways to protect yourself from HIV. You will have an HIV test every 4 weeks. After the study, you will be referred to continue PrEP at the clinic of your choice, including here at the Thika site clinic itself. If we find that you have HIV, PrEP will be immediately discontinued. You will be started on free HIV treatment immediately as per the Kenya national guidelines and will then refer you to a nearby HIV care services or at a clinic of your choice for follow up at completion of this trial. You will also receive immediate counseling about programs to reduce the chance of passing HIV to the baby (PMTCT).

New findings: We will provide you with any new information that we learn during the study, which might affect your willingness to participate

BENEFITS

You may get no direct benefit from being in this study. We will inform you about any test results that are done that could be useful for your health care. You or others may benefit in the future from information learned in this study. You also may get some personal satisfaction from being part of research on HIV. While enrolled in the study you will get all services available to those not in the study such as access to oral PrEP (which is known to help prevent HIV infection), counseling for reducing your risk of giving HIV to or getting HIV from your sexual partner, and free condoms. For care and treatment that is not available at the clinic, study staff will tell you about other places where care and treatment may be available. Your participation may also contribute to understanding HIV prevention in women in Africa, which will help others in the future.

COSTS TO YOU

There is no cost to you for being in this study. Treatments available to you by the study will be free of charge.

REASONS WHY YOU MAY BE WITHDRAWN FROM THE STUDY
You may be removed from the study without your consent for the following reasons:

- The study is stopped or canceled.
- You are not willing to find out your HIV test result.
- You are put in prison
- The study staff feel that staying in the study would be harmful to you or your baby.
- You are not able to attend study visits or complete the study procedures.

ALTERNATIVES TO PARTICIPATION
There may be other studies going on here or in the community that you or your partner may be eligible for. You may also be able to access PrEP through other research studies going on here or in other clinics in the community. If you wish, the study staff will tell you about other studies or healthcare facilities which offer PrEP that we know about. There also may be other places you can go for HIV counseling and testing. We will tell you about those places if you wish.

REIMBURSEMENT
You will receive reimbursement for your transport for visiting the clinic, and 400 KSh for your time and effort at each scheduled study visit. All visits that include collection of swabs will be reimbursed 1000 KSh.

CONFIDENTIALITY
Government or university staff sometimes review studies such as this one to make sure they are being done safely and legally. If a review of this study takes place, your records may be examined. The reviewers will protect your privacy. The study records will not be used to put you at risk of legal harm. All study staff will make every effort to protect the privacy of your personal information but cannot completely guarantee this. Any sample from you or information about you will be identified only by code and not by name. The link between your name and code will be kept in a secure location at the clinic only. The U.S. Food and Drug Administration (FDA) reserves the right to review study data that may contain identifying information. Any publication of this study will not use your name or identify you personally. Certain people and organizations will need to see, copy, and use your health data so that they can do their part in the study. They are called 'authorized users'. The records of your tests may be reviewed by study staff and representatives of the following authorized users:

- Kenya Medical Research Institute (KEMRI) Scientific and Ethics Review Unit (SERU)
- Kenya Pharmacy and Poisons Board
- University of Washington, including study monitors
- US National Institutes of Health
- Kenya Ministry of Health
- University of Colorado
- Other local, U.S. and international regulatory entities

The review of records may be done on-site or remotely through secured authorized computer programs. Remote monitoring visits may be performed in place of, or in addition to onsite visits to ensure the safety of study participants and data integrity.

We have a Certificate of Confidentiality from the NIH. These protections only apply to data held in the United States. This helps us protect your privacy. The certificate means that we do not have to give out information, documents, or samples that could identify you even if we are asked to by a court of law in the United States. We will use the Certificate to resist any demands for identifying information. We can't use the Certificate to withhold your research information if you give your written consent to give it to an insurer, employer, or other person. Also, you or a member of your family can share information about yourself or your part in this research if you wish. There are some limits to this protection. We will voluntarily provide the information to:

- a member of the United States government who needs it in order to audit or evaluate the research.
- individuals at the institution(s) conducting the research, the funding agency, and other groups involved in the research, if they need the information to make sure the research is being done correctly.
- individuals who want to conduct secondary research if allowed by federal regulations and according to your consent for future research use as described in this form.
- to relevant authorities as required by other Federal, State, or local laws.

The Certificate expires when the NIH funding for this study ends. Currently this is 31st January 2025. Any data collected after expiration is not protected as described above. Data collected prior to expiration will continue to be protected.

RESEARCH-RELATED INJURY

We do not anticipate any research related injury. However, if you feel you are injured from participating in this study, you will be offered care at the study clinic, free of charge. For research related injury, please call the 24-hour emergency number: 0736464299.There is not a program of monetary compensation through this institution. The NIH also does not provide direct compensation for research related injuries. If you need medical care that we cannot provide, we will refer you to the appropriate services or organizations that can provide care for the injury. You do not give up any legal rights by signing this consent form. You do not waive any right to seek payment or give up any legal rights by signing this consent form.

PROBLEMS OR QUESTIONS

If you ever have any questions about this study, or if you have a research-related injury, you should contact Catherine Kiptinness at the Thika Partners in Prevention Study clinic at Tel. 067 2222561/0725 641110. For research related injury, please call the 24-hour emergency number: 0736464299. If you have questions about your rights as a research participant, you should contact: The Head, Scientific and Ethics Review Unit (SERU) Kenya Medical Research Institute (KEMRI), P.O Box 54840-00202 Nairobi Phone No. 0717719477, Email address: seru@kemri.org. A description of this study will also be available on https://www.clinicaltrials.gov as required by U.S. Law. This Web site will not include information that can identify you. At most, the Web site will include a summary of the results. You can search this Web site at any time.

STATEMENT OF CONSENT AND SIGNATURES

I have read the study enrollment consent for this study, or had it read to me. I have discussed the information with study staff. My questions have been answered. I understand that my decision whether or not to take part in the study is voluntary. I understand that if I decide to join the study

I may withdraw at any time. If I refuse to be in the study or decide to stop being in the study I will have no loss of benefits to which I am already entitled. By signing this form, I do not give up any rights that I have as a research participant.

______________________	__________________________	________________
Participant Name (print)	Participant Signature/Thumbprint	Date
____________________	__________________________	________________
Study Staff Name (print)	Study Staff Signature	Date
__________________	__________________________	________________
Witness Name (print)	Witness Signature	Date

SPECIMEN STORAGE AND USE OF YOUR DATA AND SAMPLES FOR FUTURE STUDIES:
Please initial and date one option:

___________ I agree for my specimen/information to be used for future research and shared with other researchers without my additional consent without identifiers

___________ I DO NOT agree for my specimen/information to be used for future research or shared with other researchers with or without identifiers

STATEMENT OF CONSENT: BIOMETRIC FINGERPRINT
Please initial and date one option:

___________ I agree to have my fingerprint taken

____________I DO NOT agree to have my fingerprint taken

______________________	__________________________	________________
Participant Name (print)	Participant Signature/Thumbprint	Date
____________________	__________________________	________________
Study Staff Name (print)	Study Staff Signature	Date
__________________	__________________________	________________
Witness Name (print)	Witness Signature	Date

Copies to;
1. Investigators
2. Study participant
