# Supplementary material for: Adherence thresholds for emtricitabine-tenofovir disoproxil fumarate preexposure prophylaxis against HIV acquisition in cisgender women: A randomized directly observed dosing study
Source: PLoS Med. 2025 Sep 9;22(9):e1004732. doi: 10.1371/journal.pmed.1004732 (PMC12435667; doi:10.1371/journal.pmed.1004732)
Supplement: S3 File — (PDF) [file pmed.1004732.s006.pdf]

Pharmacology of TDF-FTC Pre-exposure Prophylaxis in Kenyan Cisgender women

The Women TDF-FTC Benchmark Study

Statistical Analysis Plan

Version 1.0

Based on Protocol Version 1.0 May 20, 2021

Authors:

Samantha MaWhinney, ScD

Kenneth K. Mugwanya, MBChB, MS, PhD

Peter L. Anderson, PharmD

Mary Morrow, MS

Analysis plan

We wish to describe the concentration kinetics of TFV-DP in African cisgender women. Analyses will utilize R software using reproducible practices.

1. **Dose proportionality in DBS**
   - Dose proportionality will be assessed using the power model
     - ln(TFV-DP) = μ + beta*ln(dosing study arm) + error
   - Week 8 TFV-DP concentrations will be used for the primary analysis
   - Dose proportionality is assumed if the log(dose) coefficient is within (0.8, 1.25).

2. **Estimation of Steady state concentrations and Half-life**

- Observed TFV-DP concentrations in DBS and PBMCs will be reported for each:
  - Dosing frequency
  - Study visit:
    - DBS: Use all available concentrations at week 8
    - PBMCs: Use all available concentrations at week 2, 3, 4, 5, 6, 7, 8
  - Summary of observed TFV-DP concentrations includes:
    - Median
    - Range and Interquartile range (IQR)
    - Mean
    - Standard deviation (SD)

- Steady-state TFV-DP concentrations in DBS will be estimated for each:
  - Dosing frequency
  - Half-lives for TFV-DP in DBS
  - Estimation method:
    - One-compartment non-linear mixed effects population pharmacokinetic models
    - Uses all available concentrations across all time points
  - Summary of TFV-DP concentrations at steady-state includes:
    - Median
    - Interquartile range (IQR)

        - Mean
        - Standard deviation (SD)
        - 95% confidence interval (CI) by study arms

    - <u>TFV-DP half-life in DBS estimated using:</u>
        - First-order kinetics
        - Exponential decay fit applied to post-DOT washout period

- Predictors of TFV-DP levels in DBS will be estimated using:
    - Mixed-effects model with a b-spline transformation of study day and dosing regimen using all available data.
    - The following clinical and biological factors: creatinine clearance, hematocrit, age, and weight will be assessed.

3. <u>Updated Benchmarks for 50:50 extraction</u>

- Comparison of 70:30 vs. 50:50 DBS extraction process:
    - Linear regression on the log scale to compare quantitative concentration yield
    - Paired 3-mm punch samples from the same DBS spot analyzed
- Application of model fold difference in concentration:
    - Adjust the original 70:30 TFV-DP adherence interpretations from the DOT-DBS study
    - Original adherence thresholds for 70:30 extraction process:
        - <350 fmol/punch: <2 doses/week
        - 350-699 fmol/punch: 2-3 doses/week
        - 700-1249 fmol/punch: 4-6 doses/week
        - ≥1250 fmol/punch: 7 doses/week
    - Updated adherence thresholds will be generated for the 50:50 extraction process

4. Safety assessment:

- Frequency of grade 3 or higher adverse events
- Division of AIDS toxicity tables will be used for classification
- Analysis performed overall and by study arm
